# Genome-Scale Analysis of Programmed DNA Elimination Sites in *Tetrahymena thermophila*

**DOI:** 10.1534/g3.111.000927

**Published:** 2011-11-01

**Authors:** Joseph N. Fass, Nikhil A. Joshi, Mary T. Couvillion, Josephine Bowen, Martin A. Gorovsky, Eileen P. Hamilton, Eduardo Orias, Kyungah Hong, Robert S. Coyne, Jonathan A. Eisen, Douglas L. Chalker, Dawei Lin, Kathleen Collins

**Affiliations:** * Bioinformatics Core and; †† Genome Center, University of California, Davis, California 95616; † Department of Molecular and Cell Biology, University of California, Berkeley, California 94720; § Department of Molecular, Cellular, and Developmental Biology, University of California, Santa Barbara, California 93106; ‡ Department of Biology, University of Rochester, Rochester, New York 14627; ** J. Craig Venter Institute, Rockville, Maryland 20850; ‡‡ Department of Biology, Washington University, St. Louis, Missouri 63130

**Keywords:** Tetrahymena, ciliate nuclear dualism, community sequencing project, genome rearrangement, DNA breakage and joining

## Abstract

Genetically programmed DNA rearrangements can regulate mRNA expression at an individual locus or, for some organisms, on a genome-wide scale. Ciliates rely on a remarkable process of whole-genome remodeling by DNA elimination to differentiate an expressed macronucleus (MAC) from a copy of the germline micronucleus (MIC) in each cycle of sexual reproduction. Here we describe results from the first high-throughput sequencing effort to investigate ciliate genome restructuring, comparing Sanger long-read sequences from a *Tetrahymena thermophila* MIC genome library to the MAC genome assembly. With almost 25% coverage of the unique-sequence MAC genome by MIC genome sequence reads, we created a resource for positional analysis of MIC-specific DNA removal that pinpoints MAC genome sites of DNA elimination at nucleotide resolution. The widespread distribution of internal eliminated sequences (IES) in promoter regions and introns suggests that MAC genome restructuring is essential not only for what it removes (for example, active transposons) but also for what it creates (for example, splicing-competent introns). Consistent with the heterogeneous boundaries and epigenetically modulated efficiency of individual IES deletions studied to date, we find that IES sites are dramatically under-represented in the ∼25% of the MAC genome encoding exons. As an exception to this general rule, we discovered a previously unknown class of small (<500 bp) IES with precise elimination boundaries that can contribute the 3′ exon of an mRNA expressed during genome restructuring, providing a new mechanism for expanding mRNA complexity in a developmentally regulated manner.

Regulated genome rearrangements are an evolutionarily widespread mechanism for affecting changes in gene expression, for example, switching cell mating type, alternating surface protein presentation, or expanding the repertoire of antibody production (Rusche and Rine 2010). More extreme cases of genome remodeling by large-scale chromatin diminution have been demonstrated in diverse eukaryotes, including ciliated protozoa, parasitic nematodes, and hagfish (Kloc and Zagrodzinska 2001). In the entire phylum Ciliata, a transcriptionally active MAC is differentiated from a copy of the germline MIC by elimination of repetitive DNA (Karrer 2000; Duharcourt *et al.* 2009). This process is proposed to accomplish a defense of the phenotypically expressed genome from the influence of foreign DNA. Consistent with this hypothesis, repetitive DNA elimination in ciliates involves the same process of RNA-guided heterochromatin formation required for transposon silencing in other eukaryotes (Mochizuki and Gorovsky 2004; Yao and Chao 2005; Chalker 2008). Knowledge of how the MAC and MIC differ is fundamental to understanding the evolutionarily success of ciliates as well as for enabling studies of the chromosome structures that support meiosis and mitosis (MIC chromosomes) or chromosome segregation without classic heterochromatin (MAC chromosomes).

Among the ciliates, *Tetrahymena thermophila* has been a favorable model organism for discoveries of fundamental eukaryotic biology (Collins and Gorovsky 2005). The ∼104 Mbp MAC genome of *T. thermophila* has been sequenced and annotated, revealing a complexity of gene families comparable to that in multicellular organisms (Eisen *et al.* 2006; Coyne *et al.* 2008). Genome-scale analysis of a ciliate MIC has not yet been described. Reassociation kinetics and quantitative DNA staining methods estimate *T. thermophila* MIC genome complexity as 10–20% greater than that of the MAC (Yao and Gorovsky 1974; Gorovsky 1980), but only a handful of MIC-specific elements, known as IES, have been characterized. IES are removed from the genome of the developing MAC *en masse* in a period of only a few hours during the sexual process of conjugation (Yao and Chao 2005; Chalker 2008). Extrapolation from the frequency of IES detection by differential restriction fragment mobility of MIC *vs.* MAC DNA, based on Southern blots of a few randomly selected genome regions (Yao *et al.* 1984), suggests a number of ∼6,000 MAC genome sites of IES removal. Many sequenced IES appear to be noncoding, whereas others carry ORFs related to transposon-encoded genes (Yao and Chao 2005; Chalker 2008). No known *T. thermophila* IES interrupts a protein-coding open reading frame (ORF), although the much shorter IES of *Paramecium tetraurelia* that lack epigenetic modulation of excision frequently do (Duret *et al.* 2008).

Enabled by a Joint Genome Institute (JGI) Community Sequencing Project, we used high-throughput *T. thermophila* MIC genome sequencing to initiate the genome-scale investigation of nuclear differentiation from MIC to MAC. By aligning MIC genome Sanger sequence reads to the set of assembled MAC contigs in a manner refined for pinpointing positions of DNA elimination, we created a community resource for IES investigation. Although IES are dramatically depleted in the ∼25% of the MAC genome predicted to contain exons with ORF, as an exception, we show that one member of a new class of short IES provides an exon that changes the mRNA 3′ end of a protein expressed during genome restructuring. The demonstration that an IES can provide an exon cassette establishes a new mechanism for increasing ciliate mRNA complexity in a developmentally regulated manner.

## Materials and Methods

### Nucleic acid purification and analysis

Nuclei were purified from the inbred *T. thermophila* strain SB210 used previously in the MAC genome project, with homozygous MIC allele content. MIC isolation from MAC was performed by differential centrifugation (White *et al.* 1988). MAC contamination of the MIC preparation was estimated by nuclei counts as ∼0.1%, which adjusting for differential DNA content corresponds to a mass contamination of 1–2%. Total cellular DNA was used for PCR assays and total cellular RNA was used for Northern blots. Primers are listed in supporting information, Table S1. Northern blots used hexamer-primed radiolabeled probes synthesized from double-stranded DNA templates.

### Library sequencing and read alignments

Paired-end (b1/g1) reads from an ∼8 kbp MIC DNA fragment library in pMCL are available from the NCBI trace archives (Project ID 32845). After vector sequence removal, reads were retrimmed for average qualities at least Q20 and aligned using BWA-SW default settings (bio-bwa.sourceforge.net). Read segments were designated by alignment order as aln1, aln2, etc. If more than 10 bp did not align from one side of the read only, the alignment was segregated to a separate browser track as a candidate indicator of IES position. Fully mapping and one-sided-mapping reads that could align at more than one MAC genome location were segregated to a separate set of multimapper browser tracks.

### Browser reference genome design and annotations

MIC library reads were aligned to a concatenation of MAC genome contigs ordered by decreasing size. This reference genome contains 103,002,206 bp of *T. thermophila* MAC genome project sequence with 10 kbp blocks of N added between contigs, for a total of 114,482,206 bp. Browser track annotation of candidate IES sites allowed no more than 9 bp of overlap between the MAC-matching portions of convergent L and R reads (described below). The mapped sequence reads, IES annotations, previously predicted *T. thermophila* genes (indicated by TTHERM number), and their individual predicted exons (Coyne *et al.* 2008) are aligned in an open-access community resource with the UCSC genome browser format (gb.genomecenter.ucdavis.edu, clade Alveolata, genome *T. thermophila*, Sep 2009 assembly).

## Results and Discussion

### Library construction and read alignments

Previous shotgun sequencing and assembly of the *T. thermophila* MAC used paired-end Sanger reads from 2–6 kbp DNA fragment libraries (Eisen *et al.* 2006). In pilot Sanger sequencing trials of MIC genome libraries, an ∼8 kbp insert DNA library was the maximal insert size to give a high yield of validated reads. However, high-throughput sequencing of this MIC genome library did not yield the expected genome coverage of high-quality sequence reads, suggesting some destabilization of the 8 kbp insert library from the ∼75% AT bias of the *T. thermophila* genomes (Yao and Gorovsky 1974).

**Figure 1:**
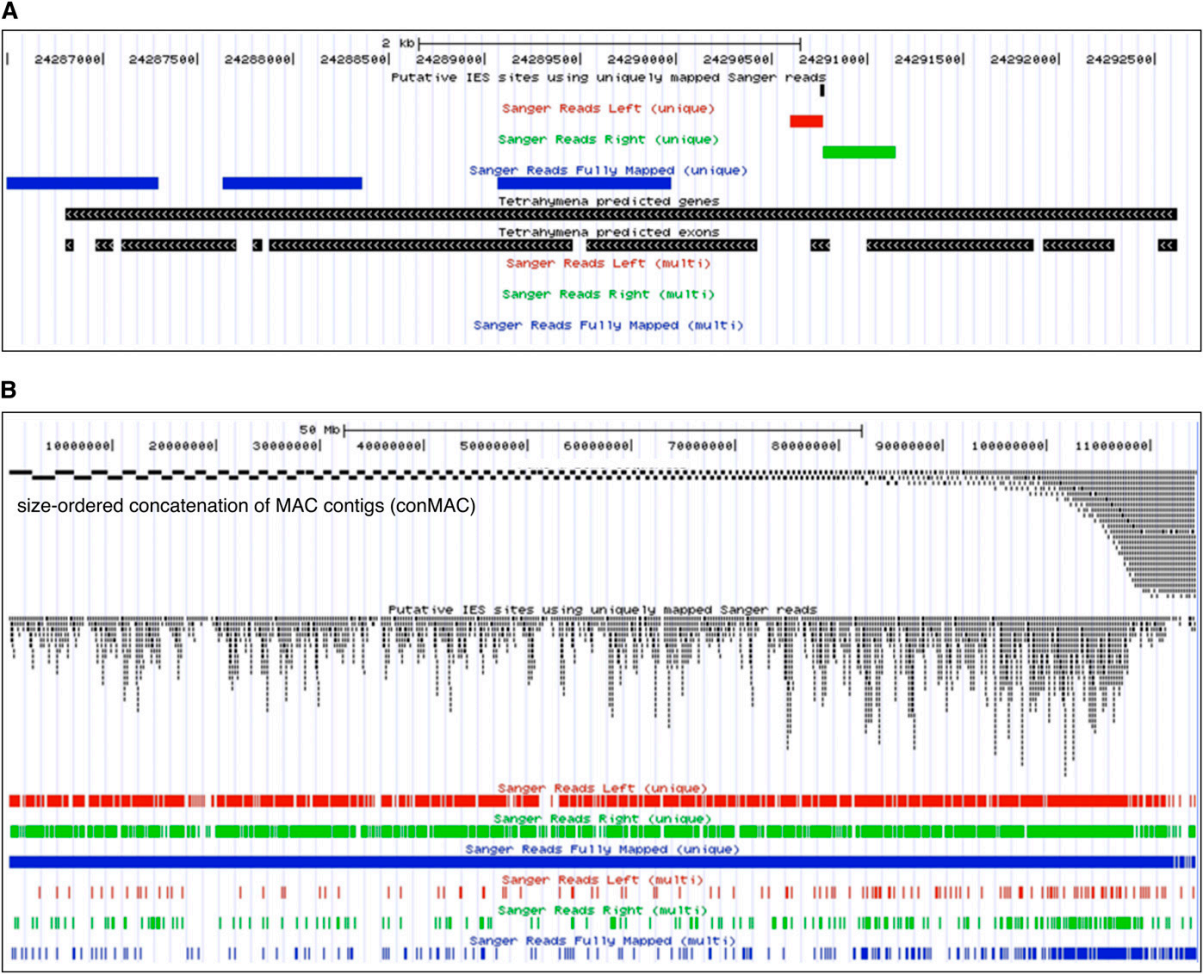
Annotation of MAC positions of IES excision. (A) A representative high-confidence win1 IES site prediction in the MAC genome. The candidate IES site (IES D) falls within an internal exon of the gene model for TTHERM_00198180, which is displayed in its entirety (see scale bar at top). The genome browser track “Putative IES sites using uniquely mapped Sanger reads” indicates positions of the win1, win2, and win3 IES predictions described in the text. The track “Sanger Reads Left (unique)” shows the extent of MAC-matching sequence within a MIC genome read that maps to the MAC with its left end but has a nonmapping sequence on its right end; the read matched a unique MAC sequence, in contrast to reads segregated to the track “Sanger Reads Left (multi).” The track “Sanger Reads Right (unique)” shows the extent of MAC-matching sequence within a MIC genome read that maps to the MAC with its right end but has a nonmapping sequence on its left end; the read matched a unique MAC sequence, in contrast to reads segregated to the track “Sanger Reads Right (multi).” The tracks “Sanger Reads Fully Mapped” show read alignments that matched the MAC without a left-end-only or right-end-only extension of nonmapping sequence. (B) Widespread MAC chromosome distribution of IES sites predicted from Sanger L and/or R read alignments. Note that the conMAC region containing the smallest MAC genome contigs at right is also overrepresented in the 10 kbp spacer blocks of N that were added between contigs; therefore, the bp amount of MAC assembly in this region is exaggerated as a proportion of conMAC length. The upper track shows the entire conMAC assembly of MAC genome contigs joined in order of decreasing length; in the browser, this track is designated as the fakeasome.

Extensively quality-filtered sequence reads were aligned to a concatenated assembly of MAC genome contigs ordered from the largest to smallest in size, termed the conMAC. The smallest MAC chromosome, encoding only ribosomal RNA, was omitted, as was the mitochondrial chromosome. The conMAC arrangement of the reference genome has the benefit of segregating the expected MIC contamination of the MAC genome assemblies, present in small scaffolds of generally low-sequence coverage (Coyne *et al.* 2008), to the right edge of the reference genome representation (gb.genomecenter.ucdavis.edu, clade Alveolata, genome *T. thermophila*, Sep 2009 assembly; see Figure 1 for genome browser track displays and annotations). The MAC contains some repetitive sequences, including RNA-coding, protein-coding, and noncoding genome segments (Eisen *et al.* 2006). Therefore, we segregated MIC genome sequence reads that aligned at multiple MAC locations to browser tracks separate from the uniquely aligning reads. The uniquely mapping MIC genome reads aligned to 22,339,139 bp of the MAC genome sequence, approaching 25% coverage of the unique-sequence fraction of the MAC genome.

### Defining MAC locations of IES

We exploited the relatively long Sanger read lengths for an alignment-based strategy of defining MAC genome positions of IES excision. Because known *T. thermophila* IES are longer than a Sanger read length, we expected some MIC library reads to align to the MAC genome at one end only, with the remaining nonmapping read extension continuing into an IES. Therefore, reads that aligned to the MAC were sorted into three categories of mapping specificity: reads fully aligned to the MAC (50,057), reads with some internal discontinuity or mismatch that could have resulted from cloned DNA rearrangement (27,002), and reads aligned to the MAC from one end only with a nonmapping >10 bp terminal extension at the other end (12,037; File S1). Reads that mapped with one end only but were at scaffold edges (File S2) were excluded from the one-end-mapper reads used for IES annotation. Nonmapping reads that did not align over any of the read length were also obtained, potentially representing MIC-specific DNA content (File S3).

We used the Sanger reads that aligned to MAC genome sequence with one end only to define candidate MAC genome positions of IES. Of these reads, 5736 aligned on the browser left side of a putative IES (L alignments), and 6301 aligned on the browser right side of a putative IES (R alignments). Because reads capable of alignment with repetitive MAC sequences can falsely suggest IES by aligning elsewhere from their locus of origin, we segregated the reads that did not map uniquely to separate browser tracks. Considering only the uniquely mapping L and R reads, we identified 3251 MAC genome locations as candidate IES positions based on the criteria of (1) departure of an L and/or R read from MAC-mapping sequence, (2) no more than 9 bp of overlap between the MAC-mapping portions of convergent L and R read sequences (allowing for up to 9 bp of IES-flanking microhomology), and (3) no read mapping to the MAC across the putative IES location (which would be evidence against IES interruption of the MAC genome). We binned these putative IES positions as one of three window (win) designations: (1) IES positions implicated by converging L and R alignments with terminal MAC-matching positions separated by no more than 9 bp (win1 category, 404 sites), (2) IES positions implicated by L alignment(s) only (win2 category, 1378 sites), or (3) R alignment(s) only (win3 category, 1469 sites). Figure 1A shows a win1 category IES site prediction (shown for IES candidate D, described below).

Some win2 and win3 category IES site predictions could be artifacts of sequence rearrangement within the library of cloned MIC genomic DNA fragments, but the convergent L and R alignments of the win1 category should pinpoint IES positions with high confidence and at nucleotide resolution. Assuming that the ∼25% coverage of the MAC genome by MIC genome sequence reads is randomly distributed, any given MAC genome position of IES excision would have a slightly less than 25% probability of coverage by an L read and the same probability of coverage by an R read. Therefore, as a rough calculation, we would expect to have defined somewhat less than 1/16^th^ of the total number of IES positions with the high-confidence win1 predictions. Given the total of 404 win1 category annotations, our extrapolation for a genome-wide tally of IES excision sites modestly exceeds the ∼6,000 sites predicted from a small sampling of loci for differential MAC and MIC restriction fragment sizes (Yao *et al.* 1984).

### Genome-wide distribution of IES positions

We first examined the distribution of predicted IES positions across MAC chromosomes. On a genome-wide scale (Figure 1B), IES positions are widely interspersed across the MAC genome. We next considered whether there was a bias to IES positions at a more local level, for example, with respect to protein-coding regions of the genome. Considering the inventory of ∼25,000 predicted MAC protein-coding genes and an average of ∼1000 nt per mRNA (Calzone *et al.* 1983a; Coyne *et al.* 2008), segments of protein-coding ORF would account for almost 25% of the MAC genome sequence. *T. thermophila* mRNAs include an average of 3.6 introns per gene (Coyne *et al.* 2008), with typically short intron and untranslated region lengths of ∼150 nt, bringing the total sequence transcribed by RNA polymerase II to ∼50% of the MAC genome. Hybridization measurements of transcript complexity support the conclusion that more than half of the MAC genome is transcribed and that more than half of the nuclear RNA complexity is represented in translated mRNA (Calzone *et al.* 1983b). Therefore, if IES positions are random with respect to MAC genome context, almost 25% of IES should interrupt a protein-coding ORF.

We sought to identify candidate exon-interrupting IES using two approaches. First, all of the high-confidence win1 category predictions of IES positions that were informatically determined to overlap predicted exons were examined manually for the validity of the gene and exon models. Candidates were excluded from further evaluation if they fell within exons of improbable gene models; for example, genes composed of a tiny exon and/or with the atypically long introns that were initially overpredicted by automated gene annotation (Coyne *et al.* 2008). Second, within the first 16,026,800 bp (>10%) of the conMAC containing the largest, telomere-to-telomere assembled MAC chromosomes, we manually surveyed candidate IES positions implicated by L-only or R-only alignments for overlap with an exon of a predicted gene. The overwhelming majority of candidate IES positions were intergenic or within an intron, consistent with the MAC genome context of the *T. thermophila* IES characterized to date. For predicted exon-interrupting IES positions, close examination usually revealed that the putative exon was part of an improbable gene model. The limited number of exceptions was investigated as described below.

### Validation of IES located in predicted exons

Following the analysis above, we retained 10 potentially reasonable predictions for putative IES that would excise from the MAC genome context of an exon (Table 1). The top three candidates (IES 1–3) met all of the selection criteria: high-confidence IES prediction by convergent L and R reads, expressed sequence tag (Coyne *et al.* 2008) and microarray-based (Miao *et al.* 2009) evidence for locus transcription (either indicated in Table 1 as “e” for Gene “expression”), and Tetrahymena Genome Database annotation of at least one functional domain in the predicted gene product (indicated in Table 1 as “a” for Gene “annotation”). Seven additional candidates (IES A–G) met a subset of these criteria (Table 1). Importantly, these IES were also supported by the presence of the same sequence of nonmapping extensions adjacent to the MAC-aligned read segment of each L or R read; this extension provided some or all of the IES sequence.

**Table 1:** Exon-interrupting IES candidates

IES	conMAC*^a^*	TTHERM*^b^*	Gene*^c^*	Reads*^d^*	MAC Junction*^e^*	Exon*^f^*	IES Length*^g^*
1	18231806	00142380	e, a	1L+1R	ttaTTAAtgg	3′	194
2	41846824	00348490	e, a	1L+2R	ttaTTAAtta	5′	453
3	62203355	00586680	e, a	2L+3R	tttTTAATTttt	Single	n.d.
A	94944570	01101620	e	1L+1R	tacATAatc	Single	483
B	15671490	00569290	e, a	1R	tcaTTAAatt	3′	337
C	64246345	00617820	e	3L+3R	cccAAtgt	3′	∼1,500
D	24290765	00198180	e, a	1L+1R	atat/cctg	Mid	∼1,200
E	95586240	01119380	e	1L+1R	aaaGAttg	Mid	∼2,000
F	42109185	00359230	e, a	1L+3R	tccTtta	Jxn 3′	n.d.
G	99551500	01259660	a	3L+1R	gtcAAata	5′	n.d.

a ConMAC, approximate browser coordinate of IES.

b TTHERM, gene model number.

c Gene, evidence for gene function based on putative mRNA expression (EST and/or microarray detection) and/or predicted protein properties (protein domain annotation) indicated by “e” and/or “a” respectively.

d Reads, number of Sanger L and/or R reads.

e MAC Junction, MAC sequence following IES removal: sequence present on both sides of the IES before elimination and retained as single-copy in the MAC is indicated in upper case; a slash separates flanking sequences joined without microhomology.

f Exon, predicted position of the IES-containing exon within the gene model: single indicates a single-exon gene model, Mid indicates an internal exon, Jxn 3′ is the intron/exon boundary of the 3′ exon.

g IES Length, actual or minimum length of IES in bp: n.d. indicates size not determined. Note that it is possible that IES length is longer than detected by PCR if the IES contains internal repeat(s).

Remarkably, for four of the candidate IES (1-2 and A-B), a complete IES sequence was possible to determine by comparison of the full-length Sanger sequence reads with each other and the MAC genome (File S4). Each of these IES was shorter than all previously characterized *T. thermophila* IES (<500 bp; Table 1, under “IES Length”), with 3–4 bp of microhomology flanking the IES that was retained in single copy in the MAC genome (Table 1, under “MAC Junction”). All four IES include a TA dinucleotide within the microhomology, and three of the four have the complete TTAA motif that is efficiently cleaved by a domesticated *piggyBac* transposase-like protein essential for conjugation in *T. thermophila* and *P. tetraurelia* (Baudry *et al.* 2009; Cheng *et al.* 2010). For the other candidate IES, a comparison of the MIC genome sequence reads with the assembled MAC revealed no or variable sequences of IES-flanking microhomology (Table 1). Lack of a specific IES-flanking sequence motif is thought to be typical in *T. thermophila*, as is the heterogeneity of MAC junctions created by IES excision (Yao *et al.* 2003; Howard-Till and Yao 2007). We suggest that the excision of different IES types could proceed by the action of different enzymes or involve different enzyme-associated factors, accounting for conservation of the TTAA motif at the boundaries of the new class of short IES but not at the boundaries of other, potentially more epigenetically regulated IES.

**Figure 2:**
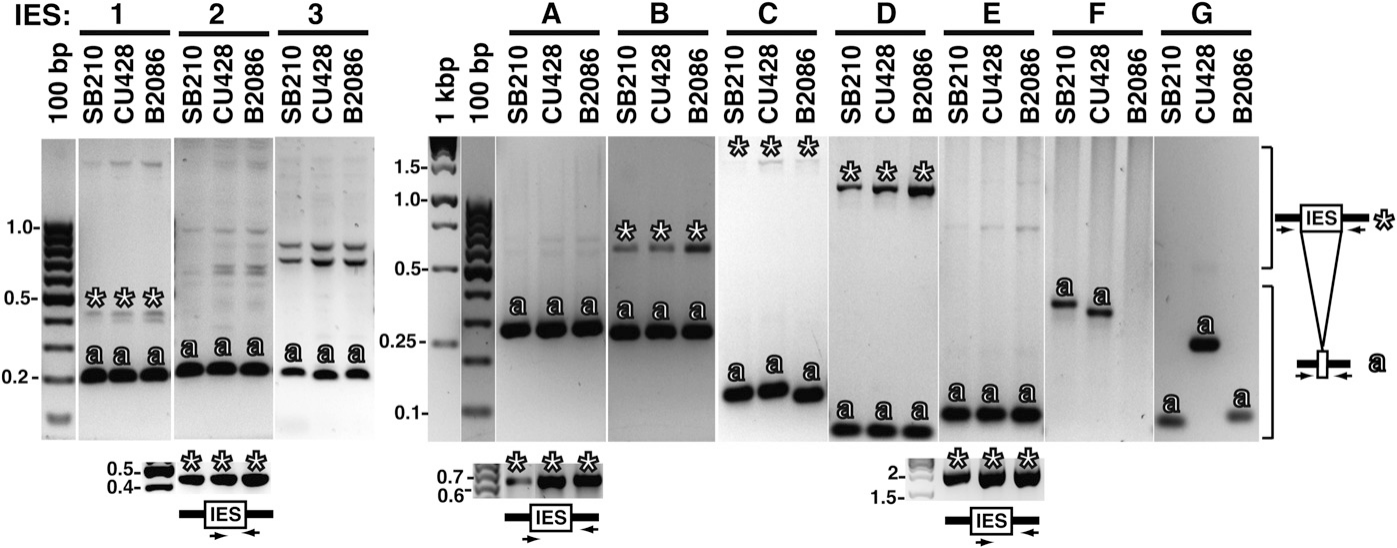
IES validation by PCR. Genomic DNA isolated from strain SB210, CU428, or B2086 was amplified by PCR using primers flanking the putative IES site in MAC-destined DNA, as schematized at right. PCR products are visualized here as the negative image of an agarose gel stained with ethidium bromide. The smaller panels below the main panels for IES 2, A, and E show IES-specific PCR amplification using primer(s) that overlap or are internal to the IES, as also schematized. Relevant DNA standards are indicated. Expected MAC genome amplification products are labeled with “
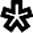
”; IES-containing PCR products are labeled with “
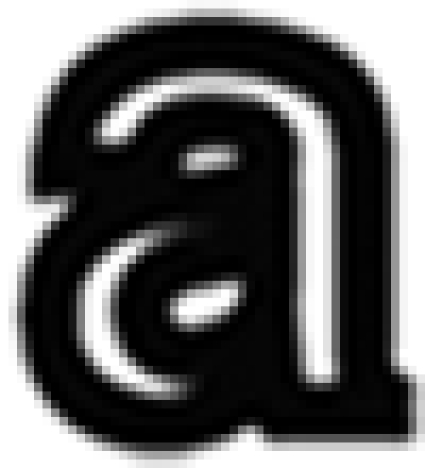
”; note that IES size could be underestimated for IES C, D, and E if the IES contains internal repeat(s).

To confirm the presence of the predicted IES with an independent approach, we used IES-flanking primers for PCR. As PCR templates, we prepared total DNA (MAC+MIC) from the sequenced inbred *T. thermophila* strain SB210 and two other MIC-homozygous inbred strains, CU428 and B2086. Initial PCR reactions were performed with primers containing MAC-destined sequences flanking the site of IES removal, which would amplify both the MAC genome junction following IES removal and, if suitably limited in length, the MIC genome IES-containing DNA as well (Figure 2, main panels and schematic at right). Additional PCR reactions were performed using one primer that spanned from MAC-destined to MIC-specific DNA and/or was entirely within the IES, designed using the nonmapping sequence extensions of the reads giving L and R alignments (Figure 2, small panels and schematics below the main panels). Because many IES are repetitive elements within the MIC genome, PCR using two entirely IES-internal primers was avoided; such reactions have the potential to amplify products from IES other than the intended MIC genome locus.

The inbred strains SB210, CU428, and B2086 were generated from independent sexual progeny and thus could differ in the MAC genome junctions created by IES removal. For most of the candidate IES, including all four of the entirely sequenced small IES (1-2 and A-B), PCR using MAC-destined sequence primers flanking the IES produced the same size of predominant amplification product from the MAC genomes of all three strains (Figure 2, designated by “
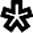
”). However, the MAC junction product for IES F was shorter in length in CU428 than in SB210 and not detectable in B2086, whereas the MAC junction product for IES G was longer in length in CU428 than in SB210 or B2086. The MAC junction product for IES C also appeared heterogeneous. Importantly, for some amplification reactions, a unique lower-abundance product of substantially longer length than the expected MAC genome amplification product could also be detected (Figure 2, designated by “
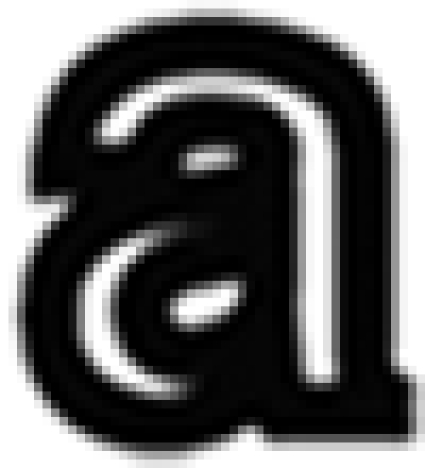
”). For cases in which a longer product was amplified with questionable specificity, we used one primer that included the MAC-destined sequence and a second primer that included the IES sequence to amplify a MIC genome product without competition from the MAC genome product (Figure 2, lower panels). Many PCR primer sequences and combinations were used to confirm a reliable IES length for the new class of <500 bp IES (IES 1-2 and A-B) and for other IES of modest length up to ∼2 kbp (IES C–E); however, repeated attempts to determine the length of IES 3, F, and G were unsuccessful (Table 1).

**Figure 3:**
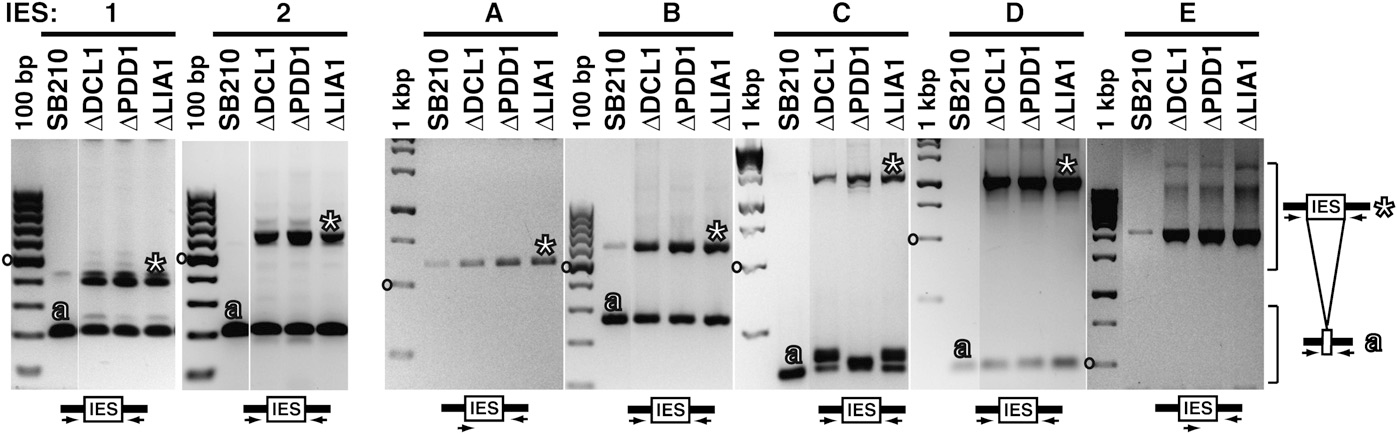
IES excision requirements. For each IES, PCR was done with the schematized primer pairs using genomic DNA isolated from the strain SB210 in vegetative growth or from the polyclonal pool of cells arrested 28 hr after initiation of conjugation for gene knockout strains lacking *DCL1*, *PDD1*, or *LIA1*. PCR products are visualized as the negative image of an agarose gel stained with ethidium bromide. DNA standard lanes have the 500 bp marker denoted with “o”; expected MAC genome amplification products are labeled with “
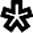
”; and IES-containing PCR products are labeled with “
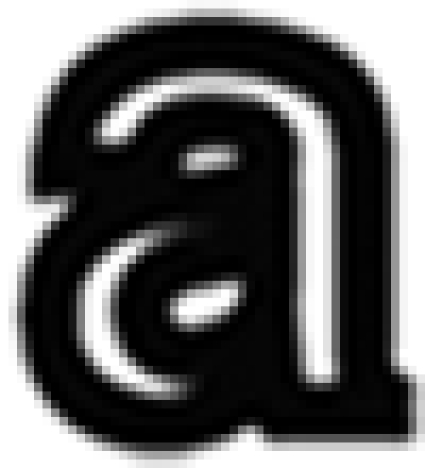
” (these labels are included in a subset of adjacent gel lanes for clarity).

**Figure 4:**
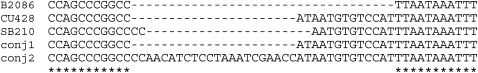
Sequence alignment of MAC junctions resulting from removal of IES C. A multiple sequence alignment is shown for cloned MAC junctions resulting from the removal of IES C, which together have three lengths and four distinct sequences. Clones were sequenced for junctions amplified from DNA of the inbred strains B2086, CU428, and SB210 (lines 1–3) or amplified from the DNA of noninbred strain crosses used in Figure 3 (lines 4–5; conj1 and conj2 indicate the two distinct sequences obtained from many cloned DNA fragment sequences). Below the alignment, “
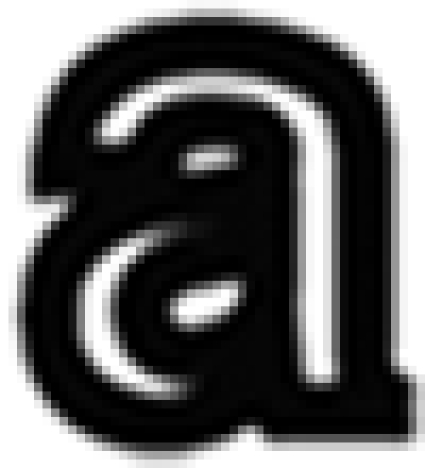
” indicates the region of consensus sequence.

Next, for the IES that could be amplified by PCR, we tested the genetic requirements for IES excision. Homokaryon gene knockout strains were mated that lacked the conjugation-essential enzyme Dicer-like 1 (*DCL1*), an IES-associated chromodomain protein (*PDD1*), or an additional factor (*LIA1*) required for IES excision (Coyne *et al.* 1999; Malone *et al.* 2005; Mochizuki and Gorovsky 2005; Rexer and Chalker 2007). Total DNA was purified from polyclonal populations of cells arrested without completing conjugation and compared with total DNA from strain SB210 in asexual vegetative growth. All of the IES appeared more abundant in conjugation-arrested cells, detected as an increase in the amplification of the IES-containing PCR product (Figure 3, designated by “
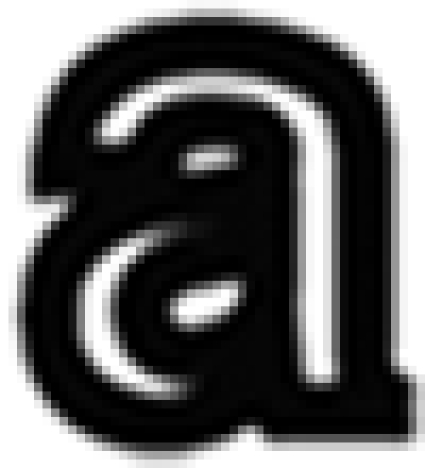
”). We conclude that removal of even the smallest known *T. thermophila* IES is directly or indirectly dependent on RNA-guided heterochromatin formation. Heterogeneous boundaries for removal of IES C were evident by PCR (Figures 2 and 3) and verified by sequencing of cloned junction amplification products (Figure 4). Thus, sequencing of MAC genomes from inbred strains other than SB210 could allow the discovery of IES sites through sequence heterogeneity at the MAC junctions of IES removal.

### IES inclusion in mRNA

**Figure 5:**
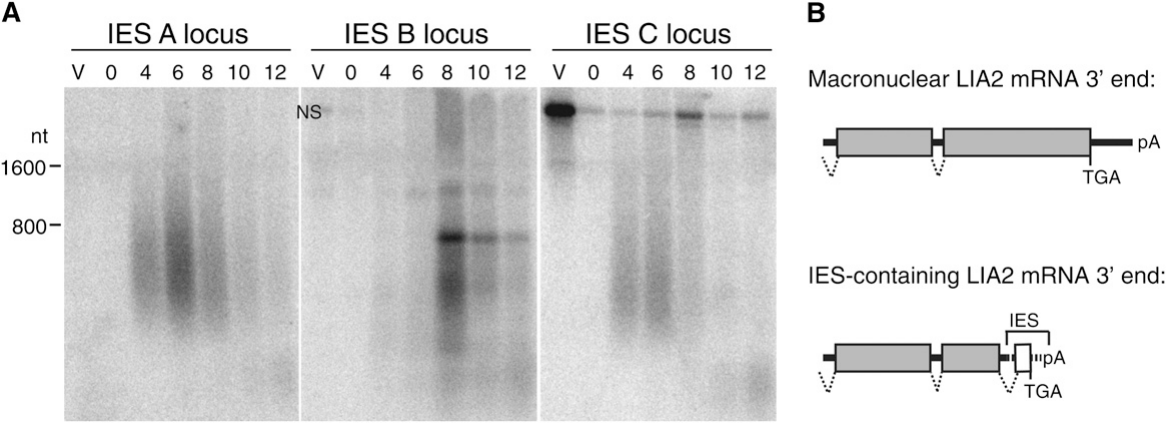
IES host gene expression. (A) Total RNA was isolated from strain SB210 in asexual vegetative growth (V) or at the indicated times after initiation of conjugation using strains SB210 and CU428 (in hours). Northern blot hybridization was performed to detect the putative mRNA region adjacent to the indicated IES. Blots are shown in size register; nonspecific signal left from a previous probing of the same membrane is indicated by “NS.” (B) Transcripts from the region of the *LIA2* locus hosting IES B were isolated by RT-PCR and sequenced. Boxes denote exons, and thick lines denote introns or untranslated regions. Gray boxes and solid lines indicate MAC-destined sequence, and the open box and dashed line indicate IES. Translation termination codons (TGA) and positions of mRNA polyadenylation (pA) are shown.

*T. thermophila* gene predictions include divergent, nonfunctional paralogs of productive genes, and as a likely consequence, some predicted genes are not detectably expressed in any lifecycle stage evaluated by EST identification or microarray hybridization (Coyne *et al.* 2008; Miao *et al.* 2009). Conversely, microarray or even EST evidence for locus expression is not certain evidence of a productive mRNA, particularly for data collected from conjugating cells with nongenic transcription. Therefore, we used Northern blot hybridization of total RNA to investigate whether putative exons flanking the six shortest, confirmed IES (IES 1-2 and A-D) were incorporated into discrete, biologically stable mRNAs. We analyzed total RNA from vegetatively growing cells and a conjugation time course extending through the interval of IES excision in the newly differentiating MAC genome (0–12 hr). No specific Northern blot hybridization signal was detected using probes containing the predicted mRNA region 5′ of IES 1, 2, or D, but probes for the regions flanking IES A, B, and C detected at least a heterogeneous smear of conjugation-specific nongenic RNA, predominantly less than 500 nucleotides (nt) in length (Figure 5A). This nongenic transcription promotes IES assembly into heterochromatin (Chalker and Yao 2001). At least for IES A, B, and C, nongenic transcription must extend far enough into the IES-flanking region to be detectable by hybridization with a probe containing an entirely MAC-destined sequence. No discrete mRNA was detected for the putative host gene of IES A.

The host gene of IES C differs from the other nine putative IES host genes in having a microarray expression profile peak in vegetatively growing cells, albeit with low signal intensity, and no detectable expression in conjugating cells. Correspondingly, Northern blot hybridization revealed a discrete >2,000 nt transcript that was most abundant in vegetatively growing cells (Figure 5A, IES C locus). Some of this transcript was also detectable in the starved cells that were mixed to initiate conjugation (time zero of the conjugation time course) and that remained as a fraction of the cells collected at all conjugation time points. Despite readily detectable mRNA production from the IES C host gene, RT-PCR attempts failed to detect the inclusion of the IES C sequence in a polyadenylated mRNA transcript.

At least two discrete forms of mRNA were detected for the host gene of IES B amid the extensive background of nongenic transcript hybridization (Figure 5A, IES B locus). The host gene of IES B encodes the helicase-domain protein Lia2, which a previous study (Yao *et al.* 2007) and our Northern blot analysis (Figure 5A) concur in detecting with conjugation-specific expression. Curiously, the peaks of mRNA and nongenic RNA expression were largely coincident, and the expression timing of nongenic transcription was shifted slightly later compared with nongenic transcription from the loci of IES A and IES C (Figure 5A). RT-PCR product sequences revealed that IES B was incorporated into polyadenylated mRNA (Figure 5B; see File S5 and GenBank EF219412.1). Remarkably, the primary transcript containing the IES was spliced to remove a portion of the predicted 3′ exon of the macronuclear *LIA2* gene and part of the IES, creating an ORF that directed both translation termination and polyadenylation within the IES (Figure 5B). As a consequence, at least one form of *LIA2* mRNA encodes a protein C-terminus translated from within IES B. Whether this mRNA is translated to produce a stably accumulated protein product remains to be established by direct analysis of Lia2 polypeptide(s).

The IES-containing *LIA2* mRNA would be transcribed in the developing MAC. Thus, it is surprising that nongenic RNA expression from the developing MAC is also readily detected, contemporary with *LIA2* mRNA. We suggest that the location of the IES in the 3′ terminal exon of the host gene (Table 1) may not be coincidental: this location provides maximum physical spacing between the initiation sites of mRNA transcription and nongenic IES transcription, which may reduce their competition. In addition, as a requisite for or result of mRNA expression, nongenic IES transcription may be delayed for the *LIA2* locus relative to loci that are not producing an mRNA product in conjugating cells. In conclusion, although IES are clearly depleted from exons on a genome-wide scale, expression of an IES-containing mRNA provides a previously unrecognized mechanism for increasing mRNA complexity in a developmentally regulated manner.

### Concluding perspectives

By positional identification of MAC genome sites of IES removal, we have created a community resource for IES discovery and characterization. Based on the *T. thermophila* content of MIC-specific *vs.* MAC-destined DNA and the estimated number of IES in the MAC genome, an average IES should be on the order of ∼2 kbp. However, we suggest that there is not an “average” IES. Instead, a true complexity of IES types seems likely to exist varying in size, sequence complexity, excision boundary precision, sensitivity to epigenetic modulation, and mechanism of elimination. Although the bulk of the MIC-specific sequence should be repetitive elements (Gorovsky 1980; Yao and Chao 2005), the new category of <500 bp IES characterized here could contribute a large number of limited-length unique sequences. IES containing repetitive DNA have functions in germline genome maintenance, for example, providing the specialized chromatin regions necessary for chromosome segregation during mitosis and meiosis (Cui and Gorovsky 2006). We suggest that other types of IES, for example, the <500 bp IES with precise elimination boundaries established by TTAA motifs, may at least infrequently gain regulatory function by altering the level or sequence of a specific mRNA. This form of regulation would be restricted to the interval of conjugation subsequent to transcriptional onset and prior to the completion of IES excision in a differentiating MAC.

On an individual level, experimentally induced failures of IES excision are not necessarily deleterious for cell survival (Chalker and Yao 1996; Yao and Chao 2005). In contrast, on genomic scale, halting the process of DNA elimination at any of a large number of intermediate steps has precluded the generation of viable sexual progeny (Chalker 2008). Our IES positional analysis suggests that few if any growth-expressed *T. thermophila* genes have exons interrupted by an IES, but the widespread distribution of IES in promoter regions and introns can account for why global failures of IES excision are incompatible with MAC function. Introns in *T. thermophila* are of limited length (even less than the length of the new class of short IES characterized in this work), and splice sites and polyadenylation signals have low sequence complexity (Eisen *et al.* 2006; Coyne *et al.* 2008). As demonstrated by the example of the *LIA2* IES above, inclusion of IES sequence in an mRNA primary transcript would therefore likely trigger additional mRNA splicing and/or premature mRNA polyadenylation. Also, due to high gene density in the *T. thermophila* MAC (Eisen *et al.* 2006; Coyne *et al.* 2008), many intergenic IES are likely to interrupt coordination between the regulatory elements of a transcriptional promoter. Thus, although IES occupy safe-haven locations in the MAC genome in the sense of tolerance for imprecise deletion boundaries, the bulk of IES length would need to be removed to produce a functional organization of many *T. thermophila* genes.

## Supplementary Material

Supporting Information
