# Supplementary material for: Genome-Scale Analysis of Programmed DNA Elimination Sites in Tetrahymena thermophila
Source: G3 (Bethesda). 2011 Nov 1;1(6):515–22. doi: 10.1534/g3.111.000927 (PMC3276166; doi:10.1534/g3.111.000927)
Supplement: Supporting Information [file supp_1_6_515__index.html]

Supporting Information 

# Genome-Scale Analysis of Programmed DNA Elimination Sites in *Tetrahymena thermophila*

## Supporting Infomation for Fass *et al.*, 2011

**Files in this Data Supplement:**

- Supporting Information - Files S1-S5 and Table S1 (PDF, 2. 6 MB)
- Table S1 - Primers (PDF, 676 KB)
- File S4 - Short IES sequences (PDF, 1.3 MB)
- File S5 - RT‐PCR product sequences for the IES‐containing *LIA2* mRNA 3' end (PDF, 716 KB)
- File S2 - Reads aligned near MAC contig edges. Also available at http://bioshare.bioinformatics.ucdavis.edu/Data/k6pd8efvnv/windows\_at\_edges\_of\_contigs.html (.html, 8 KB)
- File S1 - Reads over putative junctions of MAC‐destined and MIC‐specific sequence, maximally inclusive (not restricted for multi‐mappers). Also available at http://bioshare.bioinformatics.ucdavis.edu/Data/k6pd8efvnv/all\_windows.html (.html, 644 KB)
- File S3 - Non‐mapper reads. Also available at http://bioshare.bioinformatics.ucdavis.edu/Data/k6pd8efvnv/putative\_ies\_seqs\_unmapped.fasta (.fasta, 3 MB)
