# Supplementary material for: Genome-Scale Analysis of Programmed DNA Elimination Sites in Tetrahymena thermophila
Source: G3 (Bethesda). 2011 Nov 1;1(6):515–22. doi: 10.1534/g3.111.000927 (PMC3276166; doi:10.1534/g3.111.000927)
Supplement: Supporting Information [file supp_1.6.515_000927SI.pdf]

Files S1-S3

Sequence Files

Files S1-S3 are available at http://www.g3journal.org/lookup/suppl/doi:10.1534/g3.111.000927/-/DC1 or the links listed below.

Sequence File S1: reads over putative junctions of MAC-destined and MIC-specific sequence, maximally inclusive (not restricted for multi-mappers) http://bioshare.bioinformatics.ucdavis.edu/Data/k6pd8efvnv/all_windows.html

Sequence File S2: reads aligned near MAC contig edges
http://bioshare.bioinformatics.ucdavis.edu/Data/k6pd8efvnv/windows_at_edges_of_contigs.html

Sequence File S3: non-mapper reads
http://bioshare.bioinformatics.ucdavis.edu/Data/k6pd8efvnv/putative_ies_seqs_unmapped.fasta

Sequence File S4: short IES sequences

Sequence included in the SB210 MAC genome is in black, IES sequence is in blue, and directly repeated IES-flanking sequence retained in single copy at the MAC junction is in red and underlined.

IES 1

```
TTGTTACTGTGATTGTAAATTAGGAATATTTTATTAAACATTTTGCTATCCTAAATTTTAAGATTAGAATTCGTTTA
ATTAGTAATCTTAATTCACATTTTAGTTTATATCATATCCTTAATTTGCCTCATAATTATAATTAGTTTAACTATAA
TTATTATCTGCATTATTAACTCTTTATTTTATTTTTAATTAAAAAAAATCACTTTTTTTATTTTACAAAAAATTTTT
ATATAAAATTTACATATAACGAAAAGATAAATACAAATACAAAAGCAAAATACAAAATTTTTTTGAAAAATTTATTA
AAAAATTCATTTAATTTTAAAATTTCTTAAACTTCTCCTTATTGTCGTGAAAGTGTTAATGGGTTCGTATAACTAAG
AATTATAATTCAAATCTTAATTAATCTAGCTCTGTTGATTATTAAAGTATTAGTTGCTAGGTTCGTTTATTAAACCA
GTAACTGCATAAGGTTAACCATTTTAATTAGCATTTTGTTGGTTAAAATAAGTATCATTACTGTTAGAGTTATTTTG
```

IES 2

```
AAAGGCGGTCGCGACATCGATGTGTTTTTATTAATTGTTGTTATTTTTATTTGTTTAGTTTTATATTTTAGAAGTCT
CTTTGTTCTCTTCTTCACACATTACGGTTTTCTTGCTTTTAGTAATAATATTTTTAGAAGATTAATTTGAAGATTAA
TCTTTATCTTTATTAACACTTCAATTAATTTCTTTTTTTTATTATTTTTATTGATTCTCATCATCTGAACTATTAAA
TTAAATAAAAATATTAAATAATAACTCAATAAATAATAATTGCGCAAATAAATGAATAGGTTGGTTGGTTACCTATC
TGGTTCTATTAATGGAAGTTTCTGTAATGTGAAACCATTTACTGATTTCGTAATCTGTATCGTGCTTACATTTTAGA
CAATTTGATTACAACTAGTAGCATCTTTTTGTTTTTTCTGTTTCTTATTTTATACTAGCTGGTTAGCTAAATTTTAT
TTTTTCACTTAATCTTTTTTCATCATCTTTGTCCTTGCTTTTTTGCTATCTGAATTTTTCTATTAATTTTTTGAAAA
TGAAATTTTCAATTTTGAAATTTTTTTAAAAAATTTTTAAAAAAAATTACCCCCTGTTTTTTTAAATTGGTTTTGGAA
GTGTTAATTAGGGTAATCGTTTAATTAATATTTTATGAATTTACAGTATTTAACCCAGCTGTATTGGATGTCTAGTG
AGAGTTGGCGTTCTGTAAATTTTTGTAATAAACTCCATTTAAGCTAGCAGCCTGATTCAGGTTCATGAGATTAGTAT
TTTAATGGATACTTATGCCAGATCCATTCTGGACGTTTGCAGGAAAATTATTTTAAGGATATTTAAATGG
```

IES A

```
GAGATCGATAGCTATAAGAAAATGATATATGAAAGACTGGCAGTGACAAATCCGCTGAAGGTAGAAAATATGGCCAT
GATGCAATCAATGTTCAAATGGTTAATTAATTATCCATTGATTATTTTAATAGAGTTAGATTATTTCTAATACATAT
AGCTATTTTCAGAAAAATATTTCTCGAGTTAGAAATATAGACATAGTAAAATAAGAAATATTATTAAATATAATATT
AAAAAAAATAAAATAAATTAAAAAAAGGTTTTGAAACAGGTTTCGAACCTGATAGAAGAAAAAAATAAAAAATGTTT
AATCAAATATTTAATTTTTTGATGAAGAAATTTAAATTTTTACATCAAACATTTAATCTTGAATTAATTATTTTTTT
TACATTCAAAATGAATGAAAAAAAGGCTTAACTCGAAAAATTAAATGTTTGATTAAAAATTTTGATTTCCTCATCAA
AATATTAAATCTTTGATTAAACATTTTTTATTTTTTTTGTTAGTTCAGGTTCGAAACCTCTTTCGATACCTTTTTTT
ATTATTTTAAATATACATTAAATTTTTAAAATAATATTTATTATTCTACTAAATATTTATTTCCTACTACAGAAATA
ATTATCCGAGAATAGCAATAATCTTACTCTCTGTATGAAGTAGTAATGAAGAAGAAATTATGAAACTATCTGCTATG
CTTAAATAAATTAAGCAATCTAACTAATTACTCTTAATGTATTAGCTAGGTTTGA
```

IES B

```
AGCAGAGAATTTACTAGATGGTTTACTCATTAACACTATCTAATCTTGATTTAAATTTTTTTGAAAAAATATTCAAA
TTTTTTAAAAAAACTCTTCAAAAACTAAAAACTTTTTAAAATTTTATTTAAGTAAAAAATAAATAAACTTTAAAATA
TTTTGCTTATAAAATCTGTTAAGTAAACTGACTACATTATGTATAAAGAAGATGATTAACACAAAATAATTTTATTA
GTTTAATTGGGATTTCATTCAGAATATTCAATTTTTGATCATTCAGTCATACCAATATTATTGAGTCTCTCGCTATT
TTTGATTAAAATATAAAAAATAATTAAAATTTGAAATTAATTAAAGTGTTAAATTGGATGATTAATTATTCTATCCA
TAGGAAGAAGATGATGATGTGGAGGATTAATTCTTTGAATATGAACTTGAATTATTATTAGAATTTGAAGAATACTT
GTTAACGTTGTTATAATGAGAAGATCCATTAGAACTGCGGTATTCTGGGCTTTTTGTGCGATTATATTTATCCCATC
```

Sequence File S5: RT-PCR product sequences for the IES-containing *LIA2* mRNA 3' end

Unspliced mRNA:

GAGGTTTGGATGTTAAGGATGTCTCCCACGTATTTAATTACGATTTCCCAAAGGTTATGGAAGACTATGTCCATAGAATCGGTAGA
ACAGGTAGAGCTGGAGCATATGGTTGTGCAGTATCTTTCCTTACTTTTGAAGATGATAAAAAGATATCAAGGGAATATGTCCAAAT
GCTTCATGACGCTAAGTAAGAAATTCCCATTGATCTTCTTGATCTTGCTAGTATTAATCCCAGATACAGAACTTAATATAAAACTG
TTTCCTCTTCATACTATGATATTAAGAAATTTAACAGTGCTGATACTTCAAAGCCTTCAGAAGATCAGAATGTTTCAAATACTGCA
AATAGCAGCGACAAGTACACCAGCAGTAGCAGCTACAATTAATATAAAAGCAAAAAAGATGAAGATGACAAGAGAAGTAGAAGTAG
AAGTCCATATAAATCTGAAAATAACAACAGATGGGATAAATATAATCGCACAAAAAGCCCAGAATACCGCAGTTCTAATGGATCTT
CTCATTATAACAACGTTAACAAGTATTCTTCAAATTCTAATAATAATTCAAGTTCATATTCAAAGAATTAATCCTCCACATCATCA
TCTTCTTCCTATGGATAGAATAATTAATCATCCAATTTAACACTTTAATTAATTTCNNATTTTAATTATTTTTTATATTTTAATCA
AAAATAGCGAGAGACTCAATAATATTGGTATGACTGAATGATCAAAAATTGAATATTCTGAATGAAATCCCAATTAAACTAATAAA
ATTATTTTGTGTTAATCATCTTCTTTATACATAATGTAGTCAGTTTACTTAACAGATTTTATAAGCAAAAAAAAAAAAAAAA

Spliced mRNA:

GAGGTTTGGATGTTAAGGATGTCTCCCACGTATTTAATTACGATTTCCCAAAGGTTATGGAAGACTATGTCCATAGAATCGGTAGA
ACAGGTAGAGCTGGAGCATATGGTTGTGCAGTATCTTTCCTTACTTTTGAAGATGATAAAAAGATATCAAGGGAATATGTCCAAAT
GCTTCATGACGCTAAGTAAGAAATTCCCATTGATCTTCTTGATCTTGCTAGTATTAATCCCAGATACAGAACTTAATATAAAACTG
TTTCCTCTTCATACTATGATATTAAGAAATTTAACAGTGCTGATACTTCAAAGCCTTCAGAAAATCAGAATGTTTCAAATACTGCA
AATAGCAGCGACAAGTACACCAGCAGTAGCAGCTACAATTAATATAAAAGCAAAAAAGATGAAGATGACAAGAGAAGTAGAAGTAG
AAGTCCATATAAATCTGAAAATAACAACAGATGGGATAAATATAATCGCACAAAAAGCCCAGAATACCGCAGTTCTAATGGATCTT
CTCATTATAACAACGTTAACAACGAGAGACTCAATAATATTGGTATGACTGAATGATCAAAAATTGAATATTCTGAATGAAATCCC
AATTAAACTAATAAAATTATTTTGTGTTAATCATCTTCTTTATACATAATGTAGTCAGTTTACTTAACAGATTTTATAAGCAAAAT
AAAAAAAAAAAAAAAAAAAAAAAAAA

Table S1 Primers

Primer	Sequence	Uses
**IES 1:**		
Win1_3369L-141	CCAACAAAATGCTAATTAAAATGG	A,B
Win1_3369R-62r	TCATATCCTTAATTTGCCTCATA	A,B
Win1_3369R_junc	GAACCCATTAACACTTTCACGA	B
**IES 2:**		
win1_8019L-126	CAGGCTGCTAGCTTAAATGGA	A,B
win1_8019R-77r	TCACACATTACGGTTTTCTTGC	A,B
win1_8019IES+19	TTTTTATTGATTCTCATCATCTGAAC	B
win1_8019R_junc	CCCTAATTAACACTTCCAAAACCA	B
**IES 3:**		
win1_12075R-130	TTGGACCTGATTATTCACCTG	A
win1_12075L-64r	TTGGATCCTCTATTATAACCTCCA	A
**IES A:**		
IES5_MDSL-124	TGAAAGGCTGGCTGTGATAA	A,B
IES5_MDSR-166	CTGCGAATTTGCCTGAGATT	A
IES5_JuncR1	AGAGTAAGATTATTGCTATTCTCGGA	B
IES5_N_up	TGGCTCTCCTTCTGTTCCAC	C
IES_N_down	GGCAAATTCGCAGCTTCTTA	C
**IES B:**		
IES7_MDSL-112	GGATTGATTGGCATAAATGGA	A,B
IES7_MSDR-158	AAGCCCAGAATACCGCAGTTC	A,B,C
LIA2_N_UP2	AGTTCAAAAGCGGAGAATGC	C
Lia2_N_up2SalI	AATTAGTCGACAGTTCAAAAGCGGAGAATGCAG	D
Lia2_N_upNestSalI	AATTAGTCGACGAGGTTTGGATGTTAAGGATGTC	D
**IES C:**		
IES1_MDSL-110	TGGAAGATCTACTTCAAAGCGAAT	A,B
IES1_MDSR-31	CCAGCTAGACACCCTGTATCAA	A,B
IES1-MDSL-110Kpn	ATAGGTACCTGGAAGATCTACTTCAAAGCGAAT	A
IES1-MDSR-31Apa	ATAGGGCCCAGCTAGACACCCTGTATCAA	A
IES1_N_up1619	TTTTCCTGTTGAAAAACCTTACA	C
IES1_N_down2210r	TCCACTTTCTACCCCAAAACA	C
IES1_N_up1619SalI	AATTAGTCGACTTTTCCTGTTGAAAAACCTTACAACTGTC	D
IES1_N_upNest	GCCTCTATATGTTACAAATAGCTC	D
IES1nestIES(rev)	GGTTGGAGATGCTATTAGCTC	D: No product
IES1nest(rev)	CAGAAATTTACCAGCTAGACAC	D
**IES D:**		
IES11_MDSL-42	GGCCACAATATACTAAGGCAATTT	A,B
IES11_MDSR-34	GGCCACCTTGATACCAGTTT	A,B
**IES E:**		
IES4_MDSL-40	TTTCCAAAAATAAGTTTTTCATTGAG	A
IES4_MDSR-67	CATACGTTTGAATAAGGAGGGTTT	A,B
IES4_IESL120	GAGAAAAATGGAAATAGAGCATGA	B
**IES F:**		
IES12_MDSL-22	CAGGGTAGCTGCCATTTCTC	A
IES12_MDSL-359	ATTGCTTAAGAATTCAAATAAAAGA	A
**IES G:**		
IES2_MDSL-30	TATCAAGCCGCTAAGCCAAG	A
IES2_MDSR-67	ACCTTAAAATCTTAAAATGGATGACTC	A

Uses: A, MAC junction PCR; B, IES amplification; C, hybridization probe template; D, RT-PCR
