# Supplementary material for: Genome-Scale Analysis of Programmed DNA Elimination Sites in Tetrahymena thermophila
Source: G3 (Bethesda). 2011 Nov 1;1(6):515–22. doi: 10.1534/g3.111.000927 (PMC3276166; doi:10.1534/g3.111.000927)
Supplement: Supporting Information [file supp_1.6.515_FileS4.pdf]

Sequence File S4: short IES sequences

Sequence included in the SB210 MAC genome is in black, IES sequence is in blue, and directly repeated IES-flanking sequence retained in single copy at the MAC junction is in red and underlined.

IES 1

TTGTTACTGTGATTGTAAATTAGGAATATTTTATTAAACATTTTGCTATCCTAAATTTTAAGATTAGAATTCGTTTA
ATTAGTAATCTTAATTCACATTTTAGTTTATATCATATCCTTAATTTGCCTCATAATTATAATTAGTTTAACTATAA
TTATTATCTGCATTATTAACTCTTTATTTTATTTTTAATTAAAAAAAATCACTTTTTTTATTTTACAAAAAATTTTT
ATATAAAATTTACATATAACGAAAAGATAAATACAAATACAAAAGCAAAATACAAAATTTTTTTGAAAAATTTATTA
AAAAATTCATTTAATTTTAAAATTTCTTAAACTTCTCCTTATTGTCGTGAAAGTGTTAATGGGTTCGTATAACTAAG
AATTATAATTCAAATCTTAATTAATCTAGCTCTGTTGATTATTAAAGTATTAGTTGCTAGGTTCGTTTATTAAACCA
GTAACTGCATAAGGTTAACCATTTTAATTAGCATTTTGTTGGTTAAAATAAGTATCATTACTGTTAGAGTTATTTTG

IES 2

AAAGGCGGTCGCGACATCGATGTGTTTTTATTAATTGTTGTTATTTTTATTTGTTTAGTTTTATATTTTAGAAGTCT
CTTTGTTCTCTTCTTCACACATTACGGTTTTCTTGCTTTTAGTAATAATATTTTTAGAAGATTAATTTGAAGATTAA
TCTTTATCTTTATTAACACTTCAATTAATTTCTTTTTTTTATTATTTTTATTGATTCTCATCATCTGAACTATTAAA
TTAAATAAAAATATTAAATAATAACTCAATAAATAATAATTGCGCAAATAAATGAATAGGTTGGTTGGTTACCTATC
TGGTTCTATTAATGGAAGTTTCTGTAATGTGAAACCATTTACTGATTTCGTAATCTGTATCGTGCTTACATTTTAGA
CAATTTGATTACAACTAGTAGCATCTTTTTGTTTTTTCTGTTTCTTATTTTATACTAGCTGGTTAGCTAAATTTTAT
TTTTTCACTTAATCTTTTTTCATCATCTTTGTCCTTGCTTTTTTGCTATCTGAATTTTTCTATTAATTTTTTGAAAA
TGAAATTTTCAATTTTGAAATTTTTTTAAAAAATTTTTAAAAAAAATTACCCCCTGTTTTTTTAAATTGGTTTTGGAA
GTGTTAATTAGGGTAATCGTTTAATTAATATTTTATGAATTTACAGTATTTAACCCAGCTGTATTGGATGTCTAGTG
AGAGTTGGCGTTCTGTAAATTTTTGTAATAAACTCCATTTAAGCTAGCAGCCTGATTCAGGTTCATGAGATTAGTAT
TTTAATGGATACTTATGCCAGATCCATTCTGGACGTTTGCAGGAAAATTATTTTAAGGATATTTAAATGG

IES A

GAGATCGATAGCTATAAGAAAATGATATATGAAAGACTGGCAGTGACAAATCCGCTGAAGGTAGAAAATATGGCCAT
GATGCAATCAATGTTCAAATGGTTAATTAATTATCCATTGATTATTTTAATAGAGTTAGATTATTTCTAATACATAT
AGCTATTTTCAGAAAAATATTTCTCGAGTTAGAAATATAGACATAGTAAAATAAGAAATATTATTAAATATAATATT
AAAAAAAATAAAATAAATTAAAAAAAGGTTTTGAAACAGGTTTCGAACCTGATAGAAGAAAAAAATAAAAAATGTTT
AATCAAATATTTAATTTTTTGATGAAGAAATTTAAATTTTTACATCAAACATTTAATCTTGAATTAATTATTTTTTT
TACATTCAAAATGAATGAAAAAAAGGCTTAACTCGAAAAATTAAATGTTTGATTAAAAAATTTTGATTTCCTCATCAA
AATATTAAATCTTTGATTAAACATTTTTTATTTTTTTTGTTAGTTCAGGTTCGAAACCTCTTTCGATACCTTTTTTT
ATTATTTTAAATATACATTAAATTTTTAAAATAATATTTATTATTCTACTAAATATTTATTTCCTACTACAGAAATA
ATTATCCGAGAATAGCAATAATCTTACTCTCTGTATGAAGTAGTAATGAAGAAGAAATTATGAAACTATCTGCTATG
CTTAAATAAATTAAGCAATCTAACTAATTACTCTTAATGTATTAGCTAGGTTTGA

IES B

AGCAGAGAATTTACTAGATGGTTTACTCATTAACACTATCTAATCTTGATTTAAATTTTTTTGAAAAAATATTCAAA
TTTTTTAAAAAAACTCTTCAAAAACTAAAAACTTTTTAAAATTTTATTTAAGTAAAAAATAAATAAACTTTAAAATA
TTTTGCTTATAAAATCTGTTAAGTAAACTGACTACATTATGTATAAAGAAGATGATTAACACAAAATAATTTTATTA
GTTTAATTGGGATTTCATTCAGAATATTCAATTTTTGATCATTCAGTCATACCAATATTATTGAGTCTCTCGCTATT
TTTGATTAAAATATAAAAAATAATTAAAATTTGAAATTAATTAAAGTGTTAAATTGGATGATTAATTATTCTATCCA
TAGGAAGAAGATGATGATGTGGAGGATTAATTCTTTGAATATGAACTTGAATTATTATTAGAATTTGAAGAATACTT
GTTAACGTTGTTATAATGAGAAGATCCATTAGAACTGCGGTATTCTGGGCTTTTTGTGCGATTATATTTATCCCATC
