# Supplementary material for: Genome-Scale Analysis of Programmed DNA Elimination Sites in Tetrahymena thermophila
Source: G3 (Bethesda). 2011 Nov 1;1(6):515–22. doi: 10.1534/g3.111.000927 (PMC3276166; doi:10.1534/g3.111.000927)
Supplement: Supporting Information [file supp_1.6.515_FileS5.pdf]

Sequence File S5: RT-PCR product sequences for the IES-containing *LIA2* mRNA 3' end

Unspliced mRNA:

GAGGTTTGGATGTTAAGGATGTCTCCCACGTATTTAATTACGATTTCCCAAAGGTTATGGAAGACTATGTCCATAGAATCGGTAGA
ACAGGTAGAGCTGGAGCATATGGTTGTGCAGTATCTTTCCTTACTTTTGAAGATGATAAAAAGATATCAAGGGAATATGTCCAAAT
GCTTCATGACGCTAAGTAAGAAATTCCCATTGATCTTCTTGATCTTGCTAGTATTAATCCCAGATACAGAACTTAATATAAAACTG
TTTCCTCTTCATACTATGATATTAAGAAATTTAACAGTGCTGATACTTCAAAGCCTTCAGAAGATCAGAATGTTTCAAATACTGCA
AATAGCAGCGACAAGTACACCAGCAGTAGCAGCTACAATTAATATAAAAGCAAAAAAGATGAAGATGACAAGAGAAGTAGAAGTAG
AAGTCCATATAAATCTGAAAATAACAACAGATGGGATAAATATAATCGCACAAAAAGCCCAGAATACCGCAGTTCTAATGGATCTT
CTCATTATAACAACGTTAACAAGTATTCTTCAAATTCTAATAATAATTCAAGTTCATATTCAAAGAATTAATCCTCCACATCATCA
TCTTCTTCCTATGGATAGAATAATTAATCATCCAATTTAACACTTTAATTAATTTCNNATTTTAATTATTTTTTATATTTTAATCA
AAAATAGCGAGAGACTCAATAATATTGGTATGACTGAATGATCAAAAATTGAATATTCTGAATGAAATCCCAATTAAACTAATAAA
ATTATTTTGTGTTAATCATCTTCTTTATACATAATGTAGTCAGTTTACTTAACAGATTTTATAAGCAAAAAAAAAAAAAAAA

Spliced mRNA:

GAGGTTTGGATGTTAAGGATGTCTCCCACGTATTTAATTACGATTTCCCAAAGGTTATGGAAGACTATGTCCATAGAATCGGTAGA
ACAGGTAGAGCTGGAGCATATGGTTGTGCAGTATCTTTCCTTACTTTTGAAGATGATAAAAAGATATCAAGGGAATATGTCCAAAT
GCTTCATGACGCTAAGTAAGAAATTCCCATTGATCTTCTTGATCTTGCTAGTATTAATCCCAGATACAGAACTTAATATAAAACTG
TTTCCTCTTCATACTATGATATTAAGAAATTTAACAGTGCTGATACTTCAAAGCCTTCAGAAAATCAGAATGTTTCAAATACTGCA
AATAGCAGCGACAAGTACACCAGCAGTAGCAGCTACAATTAATATAAAAGCAAAAAAGATGAAGATGACAAGAGAAGTAGAAGTAG
AAGTCCATATAAATCTGAAAATAACAACAGATGGGATAAATATAATCGCACAAAAAGCCCAGAATACCGCAGTTCTAATGGATCTT
CTCATTATAACAACGTTAACAACGAGAGACTCAATAATATTGGTATGACTGAATGATCAAAAATTGAATATTCTGAATGAAATCCC
AATTAAACTAATAAAATTATTTTGTGTTAATCATCTTCTTTATACATAATGTAGTCAGTTTACTTAACAGATTTTATAAGCAAAAT
AAAAAAAAAAAAAAAAAAAAAAAAA
