# Supplementary material for: Genome-Scale Analysis of Programmed DNA Elimination Sites in Tetrahymena thermophila
Source: G3 (Bethesda). 2011 Nov 1;1(6):515–22. doi: 10.1534/g3.111.000927 (PMC3276166; doi:10.1534/g3.111.000927)
Supplement: Supporting Information [file supp_1.6.515_FileS2.html]

FileS2 

# Genome-Scale Analysis of Programmed DNA Elimination Sites in *Tetrahymena thermophila*

File S2 Reads aligned near MAC contig edges

Also available at http://bioshare.bioinformatics.ucdavis.edu/Data/k6pd8efvnv/windows\_at\_edges\_of\_contigs.html

win3\_7029 77351107 77351114 seq\_129 -2 4 See win3\_7029 on genome browser  
 win3\_7264 78628573 78628580 seq\_137 -3 3 See win3\_7264 on genome browser  
 win2\_8248 84824947 84824954 seq\_166 187077 187083 See win2\_8248 on genome browser  
 win3\_9976 96081383 96081390 seq\_248 -4 2 See win3\_9976 on genome browser  
 win2\_10100 96461335 96461342 seq\_253 86665 86671 See win2\_10100 on genome browser  
 win2\_10536 98641721 98641728 seq\_283 64651 64657 See win2\_10536 on genome browser  
 win2\_11822 104180018 104180025 seq\_402 20221 20227 See win2\_11822 on genome browser  
 win3\_11898 104399529 104399536 seq\_411 -2 4 See win3\_11898 on genome browser  
 win2\_12197 105531234 105531241 seq\_458 13462 13468 See win2\_12197 on genome browser  
 win3\_12297 105924504 105924511 seq\_476 -2 4 See win3\_12297 on genome browser  
 win3\_12371 106255145 106255152 seq\_493 -2 4 See win3\_12371 on genome browser  
 win2\_12521 106805031 106805038 seq\_530 7479 7485 See win2\_12521 on genome browser  
 win2\_12633 107203576 107203583 seq\_557 5600 5606 See win2\_12633 on genome browser  
 win2\_12706 107645126 107645133 seq\_596 3975 3981 See win2\_12706 on genome browser  
 win2\_12710 107659056 107659063 seq\_599 3928 3934 See win2\_12710 on genome browser  
 win2\_12750 107985476 107985483 seq\_634 3328 3334 See win2\_12750 on genome browser  
 win3\_12762 108061268 108061275 seq\_652 -2 4 See win3\_12762 on genome browser  
 win2\_12766 108242155 108242162 seq\_697 2478 2484 See win2\_12766 on genome browser  
 win2\_12781 108377691 108377698 seq\_737 2154 2160 See win2\_12781 on genome browser  
 win3\_12872 109686027 109686034 seq\_1126 -2 4 See win3\_12872 on genome browser  
 win2\_12896 109948632 109948639 seq\_1193 1334 1340 See win2\_12896 on genome browser  
 win2\_12936 110400491 110400498 seq\_1294 1256 1262 See win2\_12936 on genome browser  
 win2\_12978 110916830 110916837 seq\_1403 1190 1196 See win2\_12978 on genome browser  
 win3\_12994 111161575 111161582 seq\_1461 -2 4 See win3\_12994 on genome browser  
 win2\_13008 111307812 111307819 seq\_1486 1153 1159 See win2\_13008 on genome browser  
 win3\_13030 112185449 112185456 seq\_1638 -2 4 See win3\_13030 on genome browser  
 win2\_13046 112939625 112939632 seq\_1747 1053 1059 See win2\_13046 on genome browser  
 win2\_13066 113855624 113855631 seq\_1887 1016 1022 See win2\_13066 on genome browser  
 win3\_13070 113909703 113909710 seq\_1891 -3 3 See win3\_13070 on genome browser
