# Supplementary material for: Genome-Scale Analysis of Programmed DNA Elimination Sites in Tetrahymena thermophila
Source: G3 (Bethesda). 2011 Nov 1;1(6):515–22. doi: 10.1534/g3.111.000927 (PMC3276166; doi:10.1534/g3.111.000927)
Supplement: Supporting Information [file supp_1.6.515_FileS1.html]

FileS1 

# Genome-Scale Analysis of Programmed DNA Elimination Sites in *Tetrahymena thermophila*

File S1 Reads over putative junctions of MAC–destined and MIC–specific sequence, maximally inclusive (not restricted for multi–mappers)

Also available at http://bioshare.bioinformatics.ucdavis.edu/Data/k6pd8efvnv/all\_windows.html

win2\_1 10462 10469 seq\_1 10463 10469 See win2\_1 on genome browser  
 win3\_5 86715 86722 seq\_1 86716 86722 See win3\_5 on genome browser  
 win3\_9 149185 149192 seq\_1 149186 149192 See win3\_9 on genome browser  
 win2\_13 158339 158346 seq\_1 158340 158346 See win2\_13 on genome browser  
 win3\_17 182769 182776 seq\_1 182770 182776 See win3\_17 on genome browser  
 win3\_21 204777 204784 seq\_1 204778 204784 See win3\_21 on genome browser  
 win1\_25 211811 211816 seq\_1 211812 211816 See win1\_25 on genome browser  
 win3\_27 218518 218525 seq\_1 218519 218525 See win3\_27 on genome browser  
 win2\_31 232273 232280 seq\_1 232274 232280 See win2\_31 on genome browser  
 win3\_35 237631 237638 seq\_1 237632 237638 See win3\_35 on genome browser  
 win2\_39 237813 237820 seq\_1 237814 237820 See win2\_39 on genome browser  
 win3\_43 341882 341889 seq\_1 341883 341889 See win3\_43 on genome browser  
 win2\_47 390273 390280 seq\_1 390274 390280 See win2\_47 on genome browser  
 win3\_51 497114 497121 seq\_1 497115 497121 See win3\_51 on genome browser  
 win2\_55 505136 505143 seq\_1 505137 505143 See win2\_55 on genome browser  
 win2\_59 820219 820226 seq\_1 820220 820226 See win2\_59 on genome browser  
 win2\_63 833178 833185 seq\_1 833179 833185 See win2\_63 on genome browser  
 win3\_67 833285 833292 seq\_1 833286 833292 See win3\_67 on genome browser  
 win3\_71 835955 835962 seq\_1 835956 835962 See win3\_71 on genome browser  
 win2\_75 857126 857133 seq\_1 857127 857133 See win2\_75 on genome browser  
 win3\_79 1073649 1073656 seq\_1 1073650 1073656 See win3\_79 on genome browser  
 win3\_83 1098940 1098947 seq\_1 1098941 1098947 See win3\_83 on genome browser  
 win3\_87 1314309 1314316 seq\_1 1314310 1314316 See win3\_87 on genome browser  
 win2\_91 1320492 1320499 seq\_1 1320493 1320499 See win2\_91 on genome browser  
 win1\_95 1328325 1328332 seq\_1 1328326 1328332 See win1\_95 on genome browser  
 win2\_99 1590494 1590501 seq\_1 1590495 1590501 See win2\_99 on genome browser  
 win3\_103 1647427 1647434 seq\_1 1647428 1647434 See win3\_103 on genome browser  
 win2\_107 1667863 1667870 seq\_1 1667864 1667870 See win2\_107 on genome browser  
 win2\_111 1834384 1834391 seq\_1 1834385 1834391 See win2\_111 on genome browser  
 win3\_115 1867100 1867107 seq\_1 1867101 1867107 See win3\_115 on genome browser  
 win2\_119 1872969 1872976 seq\_1 1872970 1872976 See win2\_119 on genome browser  
 win1\_123 1967308 1967315 seq\_1 1967309 1967315 See win1\_123 on genome browser  
 win2\_127 2019877 2019884 seq\_1 2019878 2019884 See win2\_127 on genome browser  
 win2\_131 2095115 2095122 seq\_1 2095116 2095122 See win2\_131 on genome browser  
 win3\_135 2097080 2097087 seq\_1 2097081 2097087 See win3\_135 on genome browser  
 win1\_139 2098707 2098714 seq\_1 2098708 2098714 See win1\_139 on genome browser  
 win2\_143 2100804 2100811 seq\_1 2100805 2100811 See win2\_143 on genome browser  
 win3\_147 2106907 2106914 seq\_1 2106908 2106914 See win3\_147 on genome browser  
 win2\_151 2214485 2214492 seq\_1 2214486 2214492 See win2\_151 on genome browser  
 win3\_155 2226830 2226837 seq\_3825 673 679 See win3\_155 on genome browser  
 win3\_159 2245821 2245828 seq\_3825 19664 19670 See win3\_159 on genome browser  
 win3\_163 2253714 2253721 seq\_3825 27557 27563 See win3\_163 on genome browser  
 win3\_167 2253774 2253781 seq\_3825 27617 27623 See win3\_167 on genome browser  
 win2\_171 2343054 2343061 seq\_3825 116897 116903 See win2\_171 on genome browser  
 win3\_175 2349025 2349032 seq\_3825 122868 122874 See win3\_175 on genome browser  
 win3\_179 2397168 2397175 seq\_3825 171011 171017 See win3\_179 on genome browser  
 win3\_183 2466776 2466783 seq\_3825 240619 240625 See win3\_183 on genome browser  
 win3\_187 2578870 2578877 seq\_3825 352713 352719 See win3\_187 on genome browser  
 win2\_191 2579146 2579153 seq\_3825 352989 352995 See win2\_191 on genome browser  
 win2\_195 2670640 2670647 seq\_3825 444483 444489 See win2\_195 on genome browser  
 win3\_199 2709366 2709373 seq\_3825 483209 483215 See win3\_199 on genome browser  
 win2\_203 2861879 2861886 seq\_3825 635722 635728 See win2\_203 on genome browser  
 win3\_207 2892745 2892752 seq\_3825 666588 666594 See win3\_207 on genome browser  
 win2\_211 2892924 2892931 seq\_3825 666767 666773 See win2\_211 on genome browser  
 win2\_215 2903162 2903169 seq\_3825 677005 677011 See win2\_215 on genome browser  
 win2\_219 2913896 2913903 seq\_3825 687739 687745 See win2\_219 on genome browser  
 win3\_223 2931273 2931280 seq\_3825 705116 705122 See win3\_223 on genome browser  
 win3\_227 2958836 2958843 seq\_3825 732679 732685 See win3\_227 on genome browser  
 win2\_231 3137310 3137317 seq\_3825 911153 911159 See win2\_231 on genome browser  
 win3\_235 3147095 3147102 seq\_3825 920938 920944 See win3\_235 on genome browser  
 win3\_239 3254895 3254902 seq\_3825 1028738 1028744 See win3\_239 on genome browser  
 win1\_243 3263490 3263496 seq\_3825 1037333 1037338 See win1\_243 on genome browser  
 win1\_246 3282599 3282603 seq\_3825 1056442 1056445 See win1\_246 on genome browser  
 win3\_247 3286595 3286602 seq\_3825 1060438 1060444 See win3\_247 on genome browser  
 win3\_251 3550723 3550730 seq\_3825 1324566 1324572 See win3\_251 on genome browser  
 win2\_255 3578810 3578817 seq\_3825 1352653 1352659 See win2\_255 on genome browser  
 win2\_259 3602002 3602009 seq\_3825 1375845 1375851 See win2\_259 on genome browser  
 win3\_263 3604663 3604670 seq\_3825 1378506 1378512 See win3\_263 on genome browser  
 win2\_267 3672826 3672833 seq\_3825 1446669 1446675 See win2\_267 on genome browser  
 win3\_271 3672932 3672939 seq\_3825 1446775 1446781 See win3\_271 on genome browser  
 win2\_275 3711499 3711506 seq\_3825 1485342 1485348 See win2\_275 on genome browser  
 win2\_279 3760136 3760143 seq\_3825 1533979 1533985 See win2\_279 on genome browser  
 win2\_283 3762718 3762725 seq\_3825 1536561 1536567 See win2\_283 on genome browser  
 win2\_287 3815502 3815509 seq\_3825 1589345 1589351 See win2\_287 on genome browser  
 win2\_291 3855992 3855999 seq\_3825 1629835 1629841 See win2\_291 on genome browser  
 win2\_295 3872740 3872747 seq\_3825 1646583 1646589 See win2\_295 on genome browser  
 win3\_299 3988190 3988197 seq\_3825 1762033 1762039 See win3\_299 on genome browser  
 win3\_303 4063813 4063820 seq\_3825 1837656 1837662 See win3\_303 on genome browser  
 win1\_307 4065983 4065987 seq\_3825 1839826 1839829 See win1\_307 on genome browser  
 win3\_308 4110958 4110965 seq\_3825 1884801 1884807 See win3\_308 on genome browser  
 win2\_312 4140024 4140031 seq\_3825 1913867 1913873 See win2\_312 on genome browser  
 win2\_316 4189977 4189984 seq\_3825 1963820 1963826 See win2\_316 on genome browser  
 win3\_320 4375547 4375554 seq\_3825 2149390 2149396 See win3\_320 on genome browser  
 win2\_324 4435676 4435683 seq\_3836 31565 31571 See win2\_324 on genome browser  
 win2\_328 4466712 4466719 seq\_3836 62601 62607 See win2\_328 on genome browser  
 win3\_332 4474172 4474179 seq\_3836 70061 70067 See win3\_332 on genome browser  
 win3\_336 4474399 4474406 seq\_3836 70288 70294 See win3\_336 on genome browser  
 win3\_340 4482854 4482861 seq\_3836 78743 78749 See win3\_340 on genome browser  
 win3\_344 4492863 4492870 seq\_3836 88752 88758 See win3\_344 on genome browser  
 win3\_348 4564641 4564648 seq\_3836 160530 160536 See win3\_348 on genome browser  
 win3\_352 4566547 4566554 seq\_3836 162436 162442 See win3\_352 on genome browser  
 win3\_356 4572407 4572414 seq\_3836 168296 168302 See win3\_356 on genome browser  
 win3\_360 4631096 4631103 seq\_3836 226985 226991 See win3\_360 on genome browser  
 win3\_364 4646395 4646402 seq\_3836 242284 242290 See win3\_364 on genome browser  
 win2\_368 4654891 4654898 seq\_3836 250780 250786 See win2\_368 on genome browser  
 win3\_372 4668769 4668776 seq\_3836 264658 264664 See win3\_372 on genome browser  
 win3\_376 4717066 4717073 seq\_3836 312955 312961 See win3\_376 on genome browser  
 win2\_380 4779929 4779936 seq\_3836 375818 375824 See win2\_380 on genome browser  
 win2\_384 4853715 4853722 seq\_3836 449604 449610 See win2\_384 on genome browser  
 win1\_388 4869961 4869968 seq\_3836 465850 465856 See win1\_388 on genome browser  
 win3\_392 4963145 4963152 seq\_3836 559034 559040 See win3\_392 on genome browser  
 win3\_396 4973179 4973186 seq\_3836 569068 569074 See win3\_396 on genome browser  
 win3\_400 4987052 4987059 seq\_3836 582941 582947 See win3\_400 on genome browser  
 win2\_404 4999046 4999053 seq\_3836 594935 594941 See win2\_404 on genome browser  
 win3\_408 5003502 5003509 seq\_3836 599391 599397 See win3\_408 on genome browser  
 win3\_412 5005352 5005359 seq\_3836 601241 601247 See win3\_412 on genome browser  
 win1\_416 5148615 5148622 seq\_3836 744504 744510 See win1\_416 on genome browser  
 win3\_420 5149329 5149336 seq\_3836 745218 745224 See win3\_420 on genome browser  
 win2\_424 5152709 5152716 seq\_3836 748598 748604 See win2\_424 on genome browser  
 win3\_428 5283262 5283269 seq\_3836 879151 879157 See win3\_428 on genome browser  
 win2\_432 5333035 5333042 seq\_3836 928924 928930 See win2\_432 on genome browser  
 win2\_436 5393164 5393171 seq\_3836 989053 989059 See win2\_436 on genome browser  
 win2\_440 5393367 5393374 seq\_3836 989256 989262 See win2\_440 on genome browser  
 win3\_444 5415229 5415236 seq\_3836 1011118 1011124 See win3\_444 on genome browser  
 win2\_448 5529356 5529363 seq\_3836 1125245 1125251 See win2\_448 on genome browser  
 win3\_452 5572656 5572663 seq\_3836 1168545 1168551 See win3\_452 on genome browser  
 win3\_456 5580246 5580253 seq\_3836 1176135 1176141 See win3\_456 on genome browser  
 win2\_460 5581733 5581740 seq\_3836 1177622 1177628 See win2\_460 on genome browser  
 win2\_464 5768440 5768447 seq\_3836 1364329 1364335 See win2\_464 on genome browser  
 win3\_468 5797323 5797330 seq\_3836 1393212 1393218 See win3\_468 on genome browser  
 win3\_472 5876787 5876794 seq\_3836 1472676 1472682 See win3\_472 on genome browser  
 win3\_476 5958767 5958774 seq\_3836 1554656 1554662 See win3\_476 on genome browser  
 win2\_480 6165367 6165374 seq\_3836 1761256 1761262 See win2\_480 on genome browser  
 win2\_484 6169398 6169405 seq\_3836 1765287 1765293 See win2\_484 on genome browser  
 win2\_488 6172691 6172698 seq\_3836 1768580 1768586 See win2\_488 on genome browser  
 win3\_492 6176804 6176811 seq\_3836 1772693 1772699 See win3\_492 on genome browser  
 win3\_496 6181748 6181755 seq\_3836 1777637 1777643 See win3\_496 on genome browser  
 win3\_500 6282818 6282825 seq\_3824 21 27 See win3\_500 on genome browser  
 win2\_504 6395786 6395793 seq\_3824 112989 112995 See win2\_504 on genome browser  
 win3\_508 6424900 6424907 seq\_3824 142103 142109 See win3\_508 on genome browser  
 win3\_512 6506979 6506986 seq\_3824 224182 224188 See win3\_512 on genome browser  
 win1\_516 6560780 6560787 seq\_3824 277983 277989 See win1\_516 on genome browser  
 win2\_520 6590157 6590164 seq\_3824 307360 307366 See win2\_520 on genome browser  
 win2\_524 6683233 6683240 seq\_3824 400436 400442 See win2\_524 on genome browser  
 win3\_528 6708525 6708532 seq\_3824 425728 425734 See win3\_528 on genome browser  
 win1\_532 6756581 6756588 seq\_3824 473784 473790 See win1\_532 on genome browser  
 win1\_536 6958977 6958982 seq\_3824 676180 676184 See win1\_536 on genome browser  
 win3\_538 6969699 6969706 seq\_3824 686902 686908 See win3\_538 on genome browser  
 win3\_542 7573883 7573890 seq\_3824 1291086 1291092 See win3\_542 on genome browser  
 win1\_546 7607828 7607834 seq\_3824 1325031 1325036 See win1\_546 on genome browser  
 win3\_549 7738263 7738270 seq\_3824 1455466 1455472 See win3\_549 on genome browser  
 win2\_553 7965081 7965088 seq\_3812 25519 25525 See win2\_553 on genome browser  
 win2\_557 7965319 7965326 seq\_3812 25757 25763 See win2\_557 on genome browser  
 win2\_561 8039892 8039899 seq\_3812 100330 100336 See win2\_561 on genome browser  
 win1\_565 8123301 8123306 seq\_3812 183739 183743 See win1\_565 on genome browser  
 win2\_567 8213731 8213738 seq\_3812 274169 274175 See win2\_567 on genome browser  
 win3\_571 8286582 8286589 seq\_3812 347020 347026 See win3\_571 on genome browser  
 win2\_575 8306867 8306874 seq\_3812 367305 367311 See win2\_575 on genome browser  
 win2\_579 8336333 8336340 seq\_3812 396771 396777 See win2\_579 on genome browser  
 win3\_583 8358823 8358830 seq\_3812 419261 419267 See win3\_583 on genome browser  
 win3\_587 8372374 8372381 seq\_3812 432812 432818 See win3\_587 on genome browser  
 win2\_591 8386949 8386956 seq\_3812 447387 447393 See win2\_591 on genome browser  
 win2\_595 8392439 8392446 seq\_3812 452877 452883 See win2\_595 on genome browser  
 win1\_599 8430103 8430109 seq\_3812 490541 490546 See win1\_599 on genome browser  
 win2\_602 8496859 8496866 seq\_3812 557297 557303 See win2\_602 on genome browser  
 win3\_606 8586416 8586423 seq\_3812 646854 646860 See win3\_606 on genome browser  
 win1\_610 8588686 8588692 seq\_3812 649124 649129 See win1\_610 on genome browser  
 win3\_613 8707018 8707025 seq\_3812 767456 767462 See win3\_613 on genome browser  
 win1\_617 8774827 8774833 seq\_3812 835265 835270 See win1\_617 on genome browser  
 win2\_620 8783146 8783153 seq\_3812 843584 843590 See win2\_620 on genome browser  
 win2\_624 8784001 8784008 seq\_3812 844439 844445 See win2\_624 on genome browser  
 win2\_628 8810636 8810643 seq\_3812 871074 871080 See win2\_628 on genome browser  
 win3\_632 8822002 8822009 seq\_3812 882440 882446 See win3\_632 on genome browser  
 win2\_636 8890808 8890815 seq\_3812 951246 951252 See win2\_636 on genome browser  
 win3\_640 8892432 8892439 seq\_3812 952870 952876 See win3\_640 on genome browser  
 win2\_644 8916278 8916285 seq\_3812 976716 976722 See win2\_644 on genome browser  
 win3\_648 8923597 8923604 seq\_3812 984035 984041 See win3\_648 on genome browser  
 win3\_652 8951809 8951816 seq\_3812 1012247 1012253 See win3\_652 on genome browser  
 win3\_656 9011778 9011784 seq\_3812 1072216 1072221 See win3\_656 on genome browser  
 win1\_659 9011840 9011845 seq\_3812 1072278 1072282 See win1\_659 on genome browser  
 win2\_661 9017798 9017805 seq\_3812 1078236 1078242 See win2\_661 on genome browser  
 win3\_665 9023858 9023865 seq\_3812 1084296 1084302 See win3\_665 on genome browser  
 win2\_669 9063762 9063769 seq\_3812 1124200 1124206 See win2\_669 on genome browser  
 win2\_673 9095310 9095317 seq\_3812 1155748 1155754 See win2\_673 on genome browser  
 win2\_677 9210025 9210032 seq\_3812 1270463 1270469 See win2\_677 on genome browser  
 win2\_681 9223164 9223171 seq\_3812 1283602 1283608 See win2\_681 on genome browser  
 win3\_685 9233629 9233636 seq\_3812 1294067 1294073 See win3\_685 on genome browser  
 win1\_689 9252237 9252244 seq\_3812 1312675 1312681 See win1\_689 on genome browser  
 win3\_693 9254008 9254015 seq\_3812 1314446 1314452 See win3\_693 on genome browser  
 win3\_697 9254071 9254078 seq\_3812 1314509 1314515 See win3\_697 on genome browser  
 win2\_701 9255678 9255685 seq\_3812 1316116 1316122 See win2\_701 on genome browser  
 win1\_705 9329691 9329698 seq\_3812 1390129 1390135 See win1\_705 on genome browser  
 win2\_709 9348651 9348658 seq\_3812 1409089 1409095 See win2\_709 on genome browser  
 win3\_713 9352832 9352839 seq\_3812 1413270 1413276 See win3\_713 on genome browser  
 win2\_717 9497251 9497258 seq\_3812 1557689 1557695 See win2\_717 on genome browser  
 win1\_721 9532617 9532623 seq\_3812 1593055 1593060 See win1\_721 on genome browser  
 win3\_724 9548989 9548996 seq\_3707 17 23 See win3\_724 on genome browser  
 win3\_728 9565321 9565328 seq\_3707 16349 16355 See win3\_728 on genome browser  
 win2\_732 9581607 9581614 seq\_3707 32635 32641 See win2\_732 on genome browser  
 win3\_736 9631852 9631859 seq\_3707 82880 82886 See win3\_736 on genome browser  
 win2\_740 9639227 9639234 seq\_3707 90255 90261 See win2\_740 on genome browser  
 win3\_744 9691666 9691673 seq\_3707 142694 142700 See win3\_744 on genome browser  
 win3\_748 9777986 9777993 seq\_3707 229014 229020 See win3\_748 on genome browser  
 win2\_752 9793760 9793767 seq\_3707 244788 244794 See win2\_752 on genome browser  
 win3\_756 9833213 9833220 seq\_3707 284241 284247 See win3\_756 on genome browser  
 win2\_760 9841220 9841227 seq\_3707 292248 292254 See win2\_760 on genome browser  
 win2\_764 9869959 9869966 seq\_3707 320987 320993 See win2\_764 on genome browser  
 win3\_768 9910466 9910473 seq\_3707 361494 361500 See win3\_768 on genome browser  
 win2\_772 9910610 9910617 seq\_3707 361638 361644 See win2\_772 on genome browser  
 win2\_776 9922283 9922290 seq\_3707 373311 373317 See win2\_776 on genome browser  
 win3\_780 10070165 10070172 seq\_3707 521193 521199 See win3\_780 on genome browser  
 win3\_784 10098226 10098233 seq\_3707 549254 549260 See win3\_784 on genome browser  
 win2\_788 10098297 10098304 seq\_3707 549325 549331 See win2\_788 on genome browser  
 win1\_792 10129226 10129231 seq\_3707 580254 580258 See win1\_792 on genome browser  
 win2\_794 10175853 10175860 seq\_3707 626881 626887 See win2\_794 on genome browser  
 win1\_798 10197442 10197449 seq\_3707 648470 648476 See win1\_798 on genome browser  
 win3\_802 10235402 10235409 seq\_3707 686430 686436 See win3\_802 on genome browser  
 win3\_806 10249459 10249466 seq\_3707 700487 700493 See win3\_806 on genome browser  
 win3\_810 10302253 10302260 seq\_3707 753281 753287 See win3\_810 on genome browser  
 win2\_814 10348637 10348644 seq\_3707 799665 799671 See win2\_814 on genome browser  
 win2\_818 10549569 10549576 seq\_3707 1000597 1000603 See win2\_818 on genome browser  
 win3\_822 10778311 10778318 seq\_3707 1229339 1229345 See win3\_822 on genome browser  
 win3\_826 10812960 10812967 seq\_3707 1263988 1263994 See win3\_826 on genome browser  
 win2\_830 10903675 10903682 seq\_3707 1354703 1354709 See win2\_830 on genome browser  
 win2\_834 10921306 10921313 seq\_3707 1372334 1372340 See win2\_834 on genome browser  
 win3\_838 10924837 10924844 seq\_3707 1375865 1375871 See win3\_838 on genome browser  
 win1\_842 10935723 10935729 seq\_3707 1386751 1386756 See win1\_842 on genome browser  
 win2\_845 10936332 10936339 seq\_3707 1387360 1387366 See win2\_845 on genome browser  
 win3\_849 10941868 10941875 seq\_3707 1392896 1392902 See win3\_849 on genome browser  
 win3\_853 10946916 10946923 seq\_3707 1397944 1397950 See win3\_853 on genome browser  
 win2\_857 11012505 11012512 seq\_3828 46107 46113 See win2\_857 on genome browser  
 win3\_861 11024360 11024367 seq\_3828 57962 57968 See win3\_861 on genome browser  
 win1\_865 11026729 11026736 seq\_3828 60331 60337 See win1\_865 on genome browser  
 win2\_869 11038310 11038317 seq\_3828 71912 71918 See win2\_869 on genome browser  
 win2\_873 11078446 11078453 seq\_3828 112048 112054 See win2\_873 on genome browser  
 win1\_877 11080747 11080752 seq\_3828 114349 114353 See win1\_877 on genome browser  
 win2\_879 11082162 11082169 seq\_3828 115764 115770 See win2\_879 on genome browser  
 win3\_883 11093993 11094000 seq\_3828 127595 127601 See win3\_883 on genome browser  
 win2\_887 11094628 11094635 seq\_3828 128230 128236 See win2\_887 on genome browser  
 win2\_891 11133885 11133892 seq\_3828 167487 167493 See win2\_891 on genome browser  
 win3\_895 11137569 11137576 seq\_3828 171171 171177 See win3\_895 on genome browser  
 win2\_899 11142857 11142864 seq\_3828 176459 176465 See win2\_899 on genome browser  
 win3\_903 11150598 11150605 seq\_3828 184200 184206 See win3\_903 on genome browser  
 win2\_907 11184180 11184187 seq\_3828 217782 217788 See win2\_907 on genome browser  
 win2\_911 11210655 11210662 seq\_3828 244257 244263 See win2\_911 on genome browser  
 win2\_915 11223553 11223560 seq\_3828 257155 257161 See win2\_915 on genome browser  
 win1\_919 11287025 11287031 seq\_3828 320627 320632 See win1\_919 on genome browser  
 win3\_922 11289652 11289659 seq\_3828 323254 323260 See win3\_922 on genome browser  
 win3\_926 11304229 11304236 seq\_3828 337831 337837 See win3\_926 on genome browser  
 win3\_930 11327136 11327143 seq\_3828 360738 360744 See win3\_930 on genome browser  
 win3\_934 11356611 11356618 seq\_3828 390213 390219 See win3\_934 on genome browser  
 win3\_938 11357293 11357300 seq\_3828 390895 390901 See win3\_938 on genome browser  
 win3\_942 11363932 11363939 seq\_3828 397534 397540 See win3\_942 on genome browser  
 win2\_946 11373276 11373283 seq\_3828 406878 406884 See win2\_946 on genome browser  
 win2\_950 11482783 11482790 seq\_3828 516385 516391 See win2\_950 on genome browser  
 win1\_954 11507381 11507388 seq\_3828 540983 540989 See win1\_954 on genome browser  
 win2\_958 11508230 11508237 seq\_3828 541832 541838 See win2\_958 on genome browser  
 win2\_962 11512410 11512417 seq\_3828 546012 546018 See win2\_962 on genome browser  
 win2\_966 11563396 11563403 seq\_3828 596998 597004 See win2\_966 on genome browser  
 win3\_970 11578410 11578417 seq\_3828 612012 612018 See win3\_970 on genome browser  
 win1\_974 11628698 11628702 seq\_3828 662300 662303 See win1\_974 on genome browser  
 win3\_975 11662070 11662077 seq\_3828 695672 695678 See win3\_975 on genome browser  
 win2\_979 12078299 12078306 seq\_3828 1111901 1111907 See win2\_979 on genome browser  
 win3\_983 12150336 12150343 seq\_3828 1183938 1183944 See win3\_983 on genome browser  
 win3\_987 12187092 12187099 seq\_3828 1220694 1220700 See win3\_987 on genome browser  
 win3\_991 12211153 12211160 seq\_3831 1219 1225 See win3\_991 on genome browser  
 win2\_995 12211348 12211355 seq\_3831 1414 1420 See win2\_995 on genome browser  
 win3\_999 12218955 12218962 seq\_3831 9021 9027 See win3\_999 on genome browser  
 win2\_1003 12238188 12238195 seq\_3831 28254 28260 See win2\_1003 on genome browser  
 win1\_1007 12242350 12242357 seq\_3831 32416 32422 See win1\_1007 on genome browser  
 win2\_1011 12245439 12245446 seq\_3831 35505 35511 See win2\_1011 on genome browser  
 win2\_1015 12249263 12249270 seq\_3831 39329 39335 See win2\_1015 on genome browser  
 win3\_1019 12249470 12249477 seq\_3831 39536 39542 See win3\_1019 on genome browser  
 win2\_1023 12254119 12254126 seq\_3831 44185 44191 See win2\_1023 on genome browser  
 win2\_1027 12268764 12268771 seq\_3831 58830 58836 See win2\_1027 on genome browser  
 win3\_1031 12279383 12279390 seq\_3831 69449 69455 See win3\_1031 on genome browser  
 win3\_1035 12295357 12295364 seq\_3831 85423 85429 See win3\_1035 on genome browser  
 win2\_1039 12362773 12362780 seq\_3831 152839 152845 See win2\_1039 on genome browser  
 win2\_1043 12370103 12370110 seq\_3831 160169 160175 See win2\_1043 on genome browser  
 win2\_1047 12386369 12386376 seq\_3831 176435 176441 See win2\_1047 on genome browser  
 win3\_1051 12387250 12387257 seq\_3831 177316 177322 See win3\_1051 on genome browser  
 win2\_1055 12390815 12390822 seq\_3831 180881 180887 See win2\_1055 on genome browser  
 win3\_1059 12413680 12413687 seq\_3831 203746 203752 See win3\_1059 on genome browser  
 win3\_1063 12417832 12417839 seq\_3831 207898 207904 See win3\_1063 on genome browser  
 win3\_1067 12419483 12419490 seq\_3831 209549 209555 See win3\_1067 on genome browser  
 win2\_1071 12446273 12446280 seq\_3831 236339 236345 See win2\_1071 on genome browser  
 win1\_1075 12465969 12465974 seq\_3831 256035 256039 See win1\_1075 on genome browser  
 win3\_1077 12477178 12477185 seq\_3831 267244 267250 See win3\_1077 on genome browser  
 win1\_1081 12485300 12485307 seq\_3831 275366 275372 See win1\_1081 on genome browser  
 win3\_1085 12532549 12532556 seq\_3831 322615 322621 See win3\_1085 on genome browser  
 win2\_1089 12572742 12572749 seq\_3831 362808 362814 See win2\_1089 on genome browser  
 win3\_1093 12634652 12634659 seq\_3831 424718 424724 See win3\_1093 on genome browser  
 win2\_1097 12652768 12652775 seq\_3831 442834 442840 See win2\_1097 on genome browser  
 win2\_1101 12670154 12670161 seq\_3831 460220 460226 See win2\_1101 on genome browser  
 win3\_1105 12670198 12670205 seq\_3831 460264 460270 See win3\_1105 on genome browser  
 win3\_1109 12685048 12685055 seq\_3831 475114 475120 See win3\_1109 on genome browser  
 win2\_1113 12717225 12717232 seq\_3831 507291 507297 See win2\_1113 on genome browser  
 win3\_1117 12736703 12736710 seq\_3831 526769 526775 See win3\_1117 on genome browser  
 win1\_1121 12760572 12760577 seq\_3831 550638 550642 See win1\_1121 on genome browser  
 win2\_1123 12762705 12762712 seq\_3831 552771 552777 See win2\_1123 on genome browser  
 win3\_1127 12766593 12766600 seq\_3831 556659 556665 See win3\_1127 on genome browser  
 win2\_1131 12776503 12776510 seq\_3831 566569 566575 See win2\_1131 on genome browser  
 win2\_1135 12804100 12804107 seq\_3831 594166 594172 See win2\_1135 on genome browser  
 win1\_1139 12857773 12857779 seq\_3831 647839 647844 See win1\_1139 on genome browser  
 win2\_1142 12999441 12999448 seq\_3831 789507 789513 See win2\_1142 on genome browser  
 win2\_1146 13084513 13084520 seq\_3831 874579 874585 See win2\_1146 on genome browser  
 win3\_1150 13152877 13152884 seq\_3831 942943 942949 See win3\_1150 on genome browser  
 win3\_1154 13184289 13184296 seq\_3831 974355 974361 See win3\_1154 on genome browser  
 win3\_1158 13186049 13186056 seq\_3831 976115 976121 See win3\_1158 on genome browser  
 win2\_1162 13186199 13186206 seq\_3831 976265 976271 See win2\_1162 on genome browser  
 win2\_1166 13190330 13190337 seq\_3831 980396 980402 See win2\_1166 on genome browser  
 win2\_1170 13249042 13249049 seq\_3831 1039108 1039114 See win2\_1170 on genome browser  
 win3\_1174 13320791 13320798 seq\_3831 1110857 1110863 See win3\_1174 on genome browser  
 win3\_1178 13341685 13341692 seq\_3831 1131751 1131757 See win3\_1178 on genome browser  
 win2\_1182 13390329 13390336 seq\_3831 1180395 1180401 See win2\_1182 on genome browser  
 win2\_1186 13420038 13420045 seq\_3831 1210104 1210110 See win2\_1186 on genome browser  
 win3\_1190 13503443 13503450 seq\_7 72289 72295 See win3\_1190 on genome browser  
 win2\_1194 13540846 13540853 seq\_7 109692 109698 See win2\_1194 on genome browser  
 win1\_1198 13548709 13548715 seq\_7 117555 117560 See win1\_1198 on genome browser  
 win2\_1201 13573040 13573047 seq\_7 141886 141892 See win2\_1201 on genome browser  
 win3\_1205 13602791 13602798 seq\_7 171637 171643 See win3\_1205 on genome browser  
 win2\_1209 13635345 13635352 seq\_7 204191 204197 See win2\_1209 on genome browser  
 win3\_1213 13686208 13686215 seq\_7 255054 255060 See win3\_1213 on genome browser  
 win1\_1217 13686894 13686901 seq\_7 255740 255746 See win1\_1217 on genome browser  
 win3\_1221 13703150 13703157 seq\_7 271996 272002 See win3\_1221 on genome browser  
 win3\_1225 13720666 13720673 seq\_7 289512 289518 See win3\_1225 on genome browser  
 win3\_1229 13727367 13727374 seq\_7 296213 296219 See win3\_1229 on genome browser  
 win1\_1233 13734244 13734248 seq\_7 303090 303093 See win1\_1233 on genome browser  
 win3\_1234 13740005 13740012 seq\_7 308851 308857 See win3\_1234 on genome browser  
 win3\_1238 13744162 13744169 seq\_7 313008 313014 See win3\_1238 on genome browser  
 win1\_1242 13818017 13818024 seq\_7 386863 386869 See win1\_1242 on genome browser  
 win3\_1246 13924949 13924956 seq\_7 493795 493801 See win3\_1246 on genome browser  
 win3\_1250 13950839 13950846 seq\_7 519685 519691 See win3\_1250 on genome browser  
 win3\_1254 13974882 13974889 seq\_7 543728 543734 See win3\_1254 on genome browser  
 win2\_1258 13978084 13978091 seq\_7 546930 546936 See win2\_1258 on genome browser  
 win2\_1262 13989712 13989719 seq\_7 558558 558564 See win2\_1262 on genome browser  
 win3\_1266 14049661 14049668 seq\_7 618507 618513 See win3\_1266 on genome browser  
 win3\_1270 14130680 14130687 seq\_7 699526 699532 See win3\_1270 on genome browser  
 win3\_1274 14162554 14162561 seq\_7 731400 731406 See win3\_1274 on genome browser  
 win3\_1278 14165710 14165717 seq\_7 734556 734562 See win3\_1278 on genome browser  
 win2\_1282 14168193 14168200 seq\_7 737039 737045 See win2\_1282 on genome browser  
 win3\_1286 14226972 14226979 seq\_7 795818 795824 See win3\_1286 on genome browser  
 win3\_1290 14276754 14276761 seq\_7 845600 845606 See win3\_1290 on genome browser  
 win3\_1294 14305854 14305861 seq\_7 874700 874706 See win3\_1294 on genome browser  
 win3\_1298 14313000 14313007 seq\_7 881846 881852 See win3\_1298 on genome browser  
 win3\_1302 14340600 14340607 seq\_7 909446 909452 See win3\_1302 on genome browser  
 win2\_1306 14375758 14375765 seq\_7 944604 944610 See win2\_1306 on genome browser  
 win3\_1310 14431820 14431827 seq\_7 1000666 1000672 See win3\_1310 on genome browser  
 win2\_1314 14434475 14434482 seq\_7 1003321 1003327 See win2\_1314 on genome browser  
 win3\_1318 14466145 14466152 seq\_7 1034991 1034997 See win3\_1318 on genome browser  
 win2\_1322 14466276 14466283 seq\_7 1035122 1035128 See win2\_1322 on genome browser  
 win2\_1326 14468925 14468932 seq\_7 1037771 1037777 See win2\_1326 on genome browser  
 win2\_1330 14471826 14471833 seq\_7 1040672 1040678 See win2\_1330 on genome browser  
 win2\_1334 14515694 14515701 seq\_7 1084540 1084546 See win2\_1334 on genome browser  
 win2\_1338 14748787 14748794 seq\_3810 115704 115710 See win2\_1338 on genome browser  
 win3\_1342 14846164 14846171 seq\_3810 213081 213087 See win3\_1342 on genome browser  
 win2\_1346 14894967 14894974 seq\_3810 261884 261890 See win2\_1346 on genome browser  
 win2\_1350 15116814 15116821 seq\_3810 483731 483737 See win2\_1350 on genome browser  
 win2\_1354 15139126 15139133 seq\_3810 506043 506049 See win2\_1354 on genome browser  
 win2\_1358 15272601 15272608 seq\_3810 639518 639524 See win2\_1358 on genome browser  
 win2\_1362 15304662 15304669 seq\_3810 671579 671585 See win2\_1362 on genome browser  
 win2\_1366 15406784 15406791 seq\_3810 773701 773707 See win2\_1366 on genome browser  
 win2\_1370 15551757 15551764 seq\_3810 918674 918680 See win2\_1370 on genome browser  
 win3\_1374 15584447 15584454 seq\_3810 951364 951370 See win3\_1374 on genome browser  
 win2\_1378 15670510 15670517 seq\_3810 1037427 1037433 See win2\_1378 on genome browser  
 win3\_1382 15671486 15671493 seq\_3810 1038403 1038409 See win3\_1382 on genome browser  
 win3\_1386 15709550 15709557 seq\_3810 1076467 1076473 See win3\_1386 on genome browser  
 win3\_1390 15740699 15740706 seq\_3810 1107616 1107622 See win3\_1390 on genome browser  
 win1\_1394 15854721 15854728 seq\_3699 68638 68644 See win1\_1394 on genome browser  
 win2\_1398 15962839 15962846 seq\_3699 176756 176762 See win2\_1398 on genome browser  
 win3\_1402 15972377 15972384 seq\_3699 186294 186300 See win3\_1402 on genome browser  
 win3\_1406 16029469 16029476 seq\_3699 243386 243392 See win3\_1406 on genome browser  
 win2\_1410 16029558 16029565 seq\_3699 243475 243481 See win2\_1410 on genome browser  
 win2\_1414 16064851 16064858 seq\_3699 278768 278774 See win2\_1414 on genome browser  
 win2\_1418 16097790 16097797 seq\_3699 311707 311713 See win2\_1418 on genome browser  
 win3\_1422 16151492 16151499 seq\_3699 365409 365415 See win3\_1422 on genome browser  
 win1\_1426 16175088 16175095 seq\_3699 389005 389011 See win1\_1426 on genome browser  
 win2\_1430 16176025 16176032 seq\_3699 389942 389948 See win2\_1430 on genome browser  
 win2\_1434 16211465 16211472 seq\_3699 425382 425388 See win2\_1434 on genome browser  
 win3\_1438 16235107 16235114 seq\_3699 449024 449030 See win3\_1438 on genome browser  
 win3\_1442 16265666 16265673 seq\_3699 479583 479589 See win3\_1442 on genome browser  
 win3\_1446 16272237 16272244 seq\_3699 486154 486160 See win3\_1446 on genome browser  
 win3\_1450 16287617 16287624 seq\_3699 501534 501540 See win3\_1450 on genome browser  
 win1\_1454 16288573 16288579 seq\_3699 502490 502495 See win1\_1454 on genome browser  
 win2\_1457 16309147 16309154 seq\_3699 523064 523070 See win2\_1457 on genome browser  
 win2\_1461 16331183 16331190 seq\_3699 545100 545106 See win2\_1461 on genome browser  
 win3\_1465 16349840 16349847 seq\_3699 563757 563763 See win3\_1465 on genome browser  
 win3\_1469 16356280 16356287 seq\_3699 570197 570203 See win3\_1469 on genome browser  
 win2\_1473 16396260 16396267 seq\_3699 610177 610183 See win2\_1473 on genome browser  
 win3\_1477 16481913 16481920 seq\_3699 695830 695836 See win3\_1477 on genome browser  
 win3\_1481 16552543 16552550 seq\_3699 766460 766466 See win3\_1481 on genome browser  
 win2\_1485 16562988 16562995 seq\_3699 776905 776911 See win2\_1485 on genome browser  
 win3\_1489 16570296 16570303 seq\_3699 784213 784219 See win3\_1489 on genome browser  
 win3\_1493 16621130 16621137 seq\_3699 835047 835053 See win3\_1493 on genome browser  
 win2\_1497 16621324 16621331 seq\_3699 835241 835247 See win2\_1497 on genome browser  
 win2\_1501 16659318 16659325 seq\_3699 873235 873241 See win2\_1501 on genome browser  
 win1\_1505 16754295 16754302 seq\_3699 968212 968218 See win1\_1505 on genome browser  
 win3\_1509 17117924 17117931 seq\_3698 243072 243078 See win3\_1509 on genome browser  
 win3\_1513 17257534 17257541 seq\_3698 382682 382688 See win3\_1513 on genome browser  
 win1\_1517 17273476 17273481 seq\_3698 398624 398628 See win1\_1517 on genome browser  
 win2\_1519 17317313 17317320 seq\_3698 442461 442467 See win2\_1519 on genome browser  
 win2\_1523 17385873 17385880 seq\_3698 511021 511027 See win2\_1523 on genome browser  
 win3\_1527 17530536 17530543 seq\_3698 655684 655690 See win3\_1527 on genome browser  
 win3\_1531 17616073 17616080 seq\_3698 741221 741227 See win3\_1531 on genome browser  
 win3\_1535 17661619 17661626 seq\_3698 786767 786773 See win3\_1535 on genome browser  
 win2\_1539 17726471 17726478 seq\_3698 851619 851625 See win2\_1539 on genome browser  
 win2\_1543 17734260 17734267 seq\_3698 859408 859414 See win2\_1543 on genome browser  
 win3\_1547 17807753 17807760 seq\_3698 932901 932907 See win3\_1547 on genome browser  
 win2\_1551 17996758 17996765 seq\_3702 38078 38084 See win2\_1551 on genome browser  
 win2\_1555 18390984 18390991 seq\_3702 432304 432310 See win2\_1555 on genome browser  
 win2\_1559 18440210 18440217 seq\_3702 481530 481536 See win2\_1559 on genome browser  
 win2\_1563 18698686 18698693 seq\_3702 740006 740012 See win2\_1563 on genome browser  
 win3\_1567 19022179 19022186 seq\_3700 20 26 See win3\_1567 on genome browser  
 win2\_1571 19553918 19553925 seq\_3700 531759 531765 See win2\_1571 on genome browser  
 win2\_1575 19587714 19587721 seq\_3700 565555 565561 See win2\_1575 on genome browser  
 win3\_1579 19838632 19838639 seq\_3700 816473 816479 See win3\_1579 on genome browser  
 win3\_1583 19884718 19884725 seq\_3700 862559 862565 See win3\_1583 on genome browser  
 win3\_1587 19946525 19946532 seq\_3700 924366 924372 See win3\_1587 on genome browser  
 win3\_1591 19966811 19966818 seq\_3700 944652 944658 See win3\_1591 on genome browser  
 win2\_1595 19971503 19971510 seq\_3700 949344 949350 See win2\_1595 on genome browser  
 win3\_1599 20119052 20119059 seq\_3829 37787 37793 See win3\_1599 on genome browser  
 win2\_1603 20126096 20126103 seq\_3829 44831 44837 See win2\_1603 on genome browser  
 win3\_1607 20201613 20201620 seq\_3829 120348 120354 See win3\_1607 on genome browser  
 win2\_1611 20226872 20226879 seq\_3829 145607 145613 See win2\_1611 on genome browser  
 win3\_1615 20249858 20249865 seq\_3829 168593 168599 See win3\_1615 on genome browser  
 win3\_1619 20262577 20262584 seq\_3829 181312 181318 See win3\_1619 on genome browser  
 win3\_1623 20287563 20287570 seq\_3829 206298 206304 See win3\_1623 on genome browser  
 win3\_1627 20450057 20450064 seq\_3829 368792 368798 See win3\_1627 on genome browser  
 win1\_1631 20614820 20614827 seq\_3829 533555 533561 See win1\_1631 on genome browser  
 win2\_1635 20725808 20725815 seq\_3829 644543 644549 See win2\_1635 on genome browser  
 win3\_1639 20746721 20746728 seq\_3829 665456 665462 See win3\_1639 on genome browser  
 win2\_1643 20874323 20874330 seq\_3829 793058 793064 See win2\_1643 on genome browser  
 win2\_1647 20881368 20881375 seq\_3829 800103 800109 See win2\_1647 on genome browser  
 win3\_1651 20921442 20921449 seq\_3829 840177 840183 See win3\_1651 on genome browser  
 win3\_1655 20941626 20941633 seq\_3829 860361 860367 See win3\_1655 on genome browser  
 win2\_1659 21083572 21083579 seq\_3829 1002307 1002313 See win2\_1659 on genome browser  
 win3\_1663 21098673 21098680 seq\_3829 1017408 1017414 See win3\_1663 on genome browser  
 win2\_1667 21119751 21119758 seq\_3829 1038486 1038492 See win2\_1667 on genome browser  
 win3\_1671 21139493 21139500 seq\_14 7305 7311 See win3\_1671 on genome browser  
 win3\_1675 21199368 21199375 seq\_14 67180 67186 See win3\_1675 on genome browser  
 win3\_1679 21213625 21213632 seq\_14 81437 81443 See win3\_1679 on genome browser  
 win3\_1683 21215331 21215338 seq\_14 83143 83149 See win3\_1683 on genome browser  
 win2\_1687 21233656 21233663 seq\_14 101468 101474 See win2\_1687 on genome browser  
 win2\_1691 21240805 21240812 seq\_14 108617 108623 See win2\_1691 on genome browser  
 win3\_1695 21244656 21244663 seq\_14 112468 112474 See win3\_1695 on genome browser  
 win1\_1699 21246514 21246521 seq\_14 114326 114332 See win1\_1699 on genome browser  
 win3\_1703 21263863 21263870 seq\_14 131675 131681 See win3\_1703 on genome browser  
 win3\_1707 21268419 21268426 seq\_14 136231 136237 See win3\_1707 on genome browser  
 win2\_1711 21284093 21284100 seq\_14 151905 151911 See win2\_1711 on genome browser  
 win2\_1715 21301379 21301386 seq\_14 169191 169197 See win2\_1715 on genome browser  
 win2\_1719 21315856 21315863 seq\_14 183668 183674 See win2\_1719 on genome browser  
 win1\_1723 21348461 21348466 seq\_14 216273 216277 See win1\_1723 on genome browser  
 win2\_1725 21367725 21367732 seq\_14 235537 235543 See win2\_1725 on genome browser  
 win3\_1729 21369717 21369724 seq\_14 237529 237535 See win3\_1729 on genome browser  
 win1\_1733 21396215 21396219 seq\_14 264027 264030 See win1\_1733 on genome browser  
 win3\_1734 21450423 21450430 seq\_14 318235 318241 See win3\_1734 on genome browser  
 win2\_1738 21481564 21481571 seq\_14 349376 349382 See win2\_1738 on genome browser  
 win3\_1742 21496037 21496044 seq\_14 363849 363855 See win3\_1742 on genome browser  
 win3\_1746 21561930 21561937 seq\_14 429742 429748 See win3\_1746 on genome browser  
 win2\_1750 21562771 21562778 seq\_14 430583 430589 See win2\_1750 on genome browser  
 win3\_1754 21604167 21604174 seq\_14 471979 471985 See win3\_1754 on genome browser  
 win2\_1758 21607225 21607232 seq\_14 475037 475043 See win2\_1758 on genome browser  
 win1\_1762 21642078 21642084 seq\_14 509890 509895 See win1\_1762 on genome browser  
 win3\_1765 21647088 21647095 seq\_14 514900 514906 See win3\_1765 on genome browser  
 win2\_1769 21662079 21662086 seq\_14 529891 529897 See win2\_1769 on genome browser  
 win2\_1773 21670875 21670882 seq\_14 538687 538693 See win2\_1773 on genome browser  
 win2\_1777 21700672 21700679 seq\_14 568484 568490 See win2\_1777 on genome browser  
 win3\_1781 21710423 21710430 seq\_14 578235 578241 See win3\_1781 on genome browser  
 win3\_1785 21713238 21713245 seq\_14 581050 581056 See win3\_1785 on genome browser  
 win3\_1789 21757216 21757223 seq\_14 625028 625034 See win3\_1789 on genome browser  
 win3\_1793 21765919 21765926 seq\_14 633731 633737 See win3\_1793 on genome browser  
 win2\_1797 21791342 21791349 seq\_14 659154 659160 See win2\_1797 on genome browser  
 win1\_1801 21812259 21812266 seq\_14 680071 680077 See win1\_1801 on genome browser  
 win1\_1805 21815027 21815033 seq\_14 682839 682844 See win1\_1805 on genome browser  
 win3\_1808 21835704 21835711 seq\_14 703516 703522 See win3\_1808 on genome browser  
 win2\_1812 21883423 21883430 seq\_14 751235 751241 See win2\_1812 on genome browser  
 win3\_1816 21887611 21887618 seq\_14 755423 755429 See win3\_1816 on genome browser  
 win3\_1820 21943282 21943289 seq\_14 811094 811100 See win3\_1820 on genome browser  
 win2\_1824 21949567 21949574 seq\_14 817379 817385 See win2\_1824 on genome browser  
 win3\_1828 21979808 21979815 seq\_14 847620 847626 See win3\_1828 on genome browser  
 win2\_1832 22015261 22015268 seq\_14 883073 883079 See win2\_1832 on genome browser  
 win3\_1836 22025651 22025658 seq\_14 893463 893469 See win3\_1836 on genome browser  
 win2\_1840 22066686 22066693 seq\_14 934498 934504 See win2\_1840 on genome browser  
 win3\_1844 22092786 22092793 seq\_14 960598 960604 See win3\_1844 on genome browser  
 win3\_1848 22105751 22105758 seq\_14 973563 973569 See win3\_1848 on genome browser  
 win3\_1852 22105950 22105957 seq\_14 973762 973768 See win3\_1852 on genome browser  
 win2\_1856 22222656 22222663 seq\_3697 97209 97215 See win2\_1856 on genome browser  
 win1\_1860 22289150 22289156 seq\_3697 163703 163708 See win1\_1860 on genome browser  
 win2\_1863 22291509 22291516 seq\_3697 166062 166068 See win2\_1863 on genome browser  
 win3\_1867 22557176 22557183 seq\_3697 431729 431735 See win3\_1867 on genome browser  
 win2\_1871 22560803 22560810 seq\_3697 435356 435362 See win2\_1871 on genome browser  
 win1\_1875 22581858 22581865 seq\_3697 456411 456417 See win1\_1875 on genome browser  
 win2\_1879 22672675 22672682 seq\_3697 547228 547234 See win2\_1879 on genome browser  
 win3\_1883 22748795 22748802 seq\_3697 623348 623354 See win3\_1883 on genome browser  
 win2\_1887 22760293 22760300 seq\_3697 634846 634852 See win2\_1887 on genome browser  
 win2\_1891 22788998 22789005 seq\_3697 663551 663557 See win2\_1891 on genome browser  
 win2\_1895 23041239 23041246 seq\_3697 915792 915798 See win2\_1895 on genome browser  
 win2\_1899 23044462 23044469 seq\_3697 919015 919021 See win2\_1899 on genome browser  
 win2\_1903 23051595 23051602 seq\_3697 926148 926154 See win2\_1903 on genome browser  
 win2\_1907 23085351 23085358 seq\_3697 959904 959910 See win2\_1907 on genome browser  
 win2\_1911 23098345 23098352 seq\_3697 972898 972904 See win2\_1911 on genome browser  
 win2\_1915 23116787 23116794 seq\_16 1804 1810 See win2\_1915 on genome browser  
 win2\_1919 23117943 23117950 seq\_16 2960 2966 See win2\_1919 on genome browser  
 win3\_1923 23126125 23126132 seq\_16 11142 11148 See win3\_1923 on genome browser  
 win2\_1927 23203588 23203595 seq\_16 88605 88611 See win2\_1927 on genome browser  
 win2\_1931 23206565 23206572 seq\_16 91582 91588 See win2\_1931 on genome browser  
 win3\_1935 23235276 23235280 seq\_16 120293 120296 See win3\_1935 on genome browser  
 win2\_1936 23235535 23235541 seq\_16 120552 120557 See win2\_1936 on genome browser  
 win1\_1939 23297113 23297120 seq\_16 182130 182136 See win1\_1939 on genome browser  
 win2\_1943 23365387 23365394 seq\_16 250404 250410 See win2\_1943 on genome browser  
 win1\_1947 23617686 23617693 seq\_16 502703 502709 See win1\_1947 on genome browser  
 win2\_1951 23642257 23642264 seq\_16 527274 527280 See win2\_1951 on genome browser  
 win1\_1955 23686064 23686068 seq\_16 571081 571084 See win1\_1955 on genome browser  
 win3\_1956 23754887 23754894 seq\_16 639904 639910 See win3\_1956 on genome browser  
 win3\_1960 23878283 23878290 seq\_16 763300 763306 See win3\_1960 on genome browser  
 win2\_1964 23932962 23932969 seq\_16 817979 817985 See win2\_1964 on genome browser  
 win3\_1968 24043772 24043779 seq\_16 928789 928795 See win3\_1968 on genome browser  
 win3\_1972 24087424 24087431 seq\_17 27441 27447 See win3\_1972 on genome browser  
 win2\_1976 24134593 24134600 seq\_17 74610 74616 See win2\_1976 on genome browser  
 win1\_1980 24140302 24140306 seq\_17 80319 80322 See win1\_1980 on genome browser  
 win3\_1981 24178337 24178344 seq\_17 118354 118360 See win3\_1981 on genome browser  
 win3\_1985 24202122 24202129 seq\_17 142139 142145 See win3\_1985 on genome browser  
 win3\_1989 24269919 24269926 seq\_17 209936 209942 See win3\_1989 on genome browser  
 win1\_1993 24290763 24290770 seq\_17 230780 230786 See win1\_1993 on genome browser  
 win2\_1997 24324803 24324810 seq\_17 264820 264826 See win2\_1997 on genome browser  
 win3\_2001 24327833 24327840 seq\_17 267850 267856 See win3\_2001 on genome browser  
 win3\_2005 24345238 24345245 seq\_17 285255 285261 See win3\_2005 on genome browser  
 win1\_2009 24353634 24353639 seq\_17 293651 293655 See win1\_2009 on genome browser  
 win2\_2011 24381397 24381404 seq\_17 321414 321420 See win2\_2011 on genome browser  
 win3\_2015 24385079 24385086 seq\_17 325096 325102 See win3\_2015 on genome browser  
 win2\_2019 24386069 24386076 seq\_17 326086 326092 See win2\_2019 on genome browser  
 win2\_2023 24388483 24388490 seq\_17 328500 328506 See win2\_2023 on genome browser  
 win2\_2027 24396566 24396573 seq\_17 336583 336589 See win2\_2027 on genome browser  
 win3\_2031 24418739 24418746 seq\_17 358756 358762 See win3\_2031 on genome browser  
 win3\_2035 24422010 24422017 seq\_17 362027 362033 See win3\_2035 on genome browser  
 win3\_2039 24424607 24424614 seq\_17 364624 364630 See win3\_2039 on genome browser  
 win2\_2043 24448493 24448500 seq\_17 388510 388516 See win2\_2043 on genome browser  
 win2\_2047 24448800 24448807 seq\_17 388817 388823 See win2\_2047 on genome browser  
 win3\_2051 24469656 24469663 seq\_17 409673 409679 See win3\_2051 on genome browser  
 win2\_2055 24551530 24551537 seq\_17 491547 491553 See win2\_2055 on genome browser  
 win2\_2059 24557821 24557828 seq\_17 497838 497844 See win2\_2059 on genome browser  
 win2\_2063 24596142 24596149 seq\_17 536159 536165 See win2\_2063 on genome browser  
 win3\_2067 24671189 24671196 seq\_17 611206 611212 See win3\_2067 on genome browser  
 win3\_2071 24674357 24674364 seq\_17 614374 614380 See win3\_2071 on genome browser  
 win2\_2075 24710450 24710457 seq\_17 650467 650473 See win2\_2075 on genome browser  
 win2\_2079 24711269 24711276 seq\_17 651286 651292 See win2\_2079 on genome browser  
 win2\_2083 24721434 24721441 seq\_17 661451 661457 See win2\_2083 on genome browser  
 win2\_2087 24721705 24721712 seq\_17 661722 661728 See win2\_2087 on genome browser  
 win2\_2091 24769622 24769629 seq\_17 709639 709645 See win2\_2091 on genome browser  
 win3\_2095 24775837 24775844 seq\_17 715854 715860 See win3\_2095 on genome browser  
 win2\_2099 24778961 24778968 seq\_17 718978 718984 See win2\_2099 on genome browser  
 win3\_2103 24799790 24799797 seq\_17 739807 739813 See win3\_2103 on genome browser  
 win2\_2107 24808221 24808228 seq\_17 748238 748244 See win2\_2107 on genome browser  
 win3\_2111 24808321 24808328 seq\_17 748338 748344 See win3\_2111 on genome browser  
 win3\_2115 24808760 24808767 seq\_17 748777 748783 See win3\_2115 on genome browser  
 win2\_2119 24811488 24811495 seq\_17 751505 751511 See win2\_2119 on genome browser  
 win1\_2123 24836155 24836160 seq\_17 776172 776176 See win1\_2123 on genome browser  
 win2\_2125 24843066 24843073 seq\_17 783083 783089 See win2\_2125 on genome browser  
 win3\_2129 24848146 24848153 seq\_17 788163 788169 See win3\_2129 on genome browser  
 win2\_2133 24851306 24851313 seq\_17 791323 791329 See win2\_2133 on genome browser  
 win2\_2137 24852792 24852799 seq\_17 792809 792815 See win2\_2137 on genome browser  
 win2\_2141 24900164 24900171 seq\_17 840181 840187 See win2\_2141 on genome browser  
 win2\_2145 24909448 24909455 seq\_17 849465 849471 See win2\_2145 on genome browser  
 win3\_2149 24912132 24912139 seq\_17 852149 852155 See win3\_2149 on genome browser  
 win2\_2153 25122733 25122740 seq\_3701 143442 143448 See win2\_2153 on genome browser  
 win2\_2157 25126875 25126882 seq\_3701 147584 147590 See win2\_2157 on genome browser  
 win2\_2161 25189514 25189521 seq\_3701 210223 210229 See win2\_2161 on genome browser  
 win3\_2165 25290587 25290594 seq\_3701 311296 311302 See win3\_2165 on genome browser  
 win2\_2169 25360857 25360864 seq\_3701 381566 381572 See win2\_2169 on genome browser  
 win3\_2173 25395151 25395158 seq\_3701 415860 415866 See win3\_2173 on genome browser  
 win1\_2177 25605438 25605445 seq\_3701 626147 626153 See win1\_2177 on genome browser  
 win2\_2181 25679829 25679836 seq\_3701 700538 700544 See win2\_2181 on genome browser  
 win2\_2185 25698718 25698725 seq\_3701 719427 719433 See win2\_2185 on genome browser  
 win2\_2189 25836245 25836252 seq\_3701 856954 856960 See win2\_2189 on genome browser  
 win3\_2193 25864144 25864151 seq\_3701 884853 884859 See win3\_2193 on genome browser  
 win2\_2197 25993218 25993225 seq\_19 112657 112663 See win2\_2197 on genome browser  
 win3\_2201 26001582 26001589 seq\_19 121021 121027 See win3\_2201 on genome browser  
 win2\_2205 26014531 26014538 seq\_19 133970 133976 See win2\_2205 on genome browser  
 win2\_2209 26025061 26025068 seq\_19 144500 144506 See win2\_2209 on genome browser  
 win3\_2213 26040157 26040164 seq\_19 159596 159602 See win3\_2213 on genome browser  
 win3\_2217 26070573 26070580 seq\_19 190012 190018 See win3\_2217 on genome browser  
 win3\_2221 26078155 26078162 seq\_19 197594 197600 See win3\_2221 on genome browser  
 win2\_2225 26081078 26081085 seq\_19 200517 200523 See win2\_2225 on genome browser  
 win3\_2229 26099172 26099179 seq\_19 218611 218617 See win3\_2229 on genome browser  
 win2\_2233 26115524 26115531 seq\_19 234963 234969 See win2\_2233 on genome browser  
 win1\_2237 26182866 26182873 seq\_19 302305 302311 See win1\_2237 on genome browser  
 win2\_2241 26188587 26188594 seq\_19 308026 308032 See win2\_2241 on genome browser  
 win2\_2245 26216316 26216323 seq\_19 335755 335761 See win2\_2245 on genome browser  
 win2\_2249 26216424 26216431 seq\_19 335863 335869 See win2\_2249 on genome browser  
 win3\_2253 26236215 26236222 seq\_19 355654 355660 See win3\_2253 on genome browser  
 win2\_2257 26243680 26243687 seq\_19 363119 363125 See win2\_2257 on genome browser  
 win3\_2261 26247310 26247317 seq\_19 366749 366755 See win3\_2261 on genome browser  
 win2\_2265 26424087 26424094 seq\_19 543526 543532 See win2\_2265 on genome browser  
 win2\_2269 26441134 26441141 seq\_19 560573 560579 See win2\_2269 on genome browser  
 win3\_2273 26468807 26468814 seq\_19 588246 588252 See win3\_2273 on genome browser  
 win2\_2277 26487278 26487285 seq\_19 606717 606723 See win2\_2277 on genome browser  
 win2\_2281 26489115 26489122 seq\_19 608554 608560 See win2\_2281 on genome browser  
 win2\_2285 26498880 26498887 seq\_19 618319 618325 See win2\_2285 on genome browser  
 win3\_2289 26519031 26519038 seq\_19 638470 638476 See win3\_2289 on genome browser  
 win3\_2293 26550171 26550178 seq\_19 669610 669616 See win3\_2293 on genome browser  
 win2\_2297 26618380 26618387 seq\_19 737819 737825 See win2\_2297 on genome browser  
 win1\_2301 26624339 26624346 seq\_19 743778 743784 See win1\_2301 on genome browser  
 win3\_2305 26628337 26628344 seq\_19 747776 747782 See win3\_2305 on genome browser  
 win3\_2309 26632835 26632842 seq\_19 752274 752280 See win3\_2309 on genome browser  
 win2\_2313 26648574 26648581 seq\_19 768013 768019 See win2\_2313 on genome browser  
 win3\_2317 26648674 26648681 seq\_19 768113 768119 See win3\_2317 on genome browser  
 win1\_2321 26653426 26653430 seq\_19 772865 772868 See win1\_2321 on genome browser  
 win2\_2322 26665114 26665121 seq\_19 784553 784559 See win2\_2322 on genome browser  
 win3\_2326 26686966 26686973 seq\_19 806405 806411 See win3\_2326 on genome browser  
 win1\_2330 26691144 26691150 seq\_19 810583 810588 See win1\_2330 on genome browser  
 win2\_2333 26716677 26716684 seq\_19 836116 836122 See win2\_2333 on genome browser  
 win3\_2337 26721883 26721890 seq\_19 841322 841328 See win3\_2337 on genome browser  
 win1\_2341 26723462 26723468 seq\_19 842901 842906 See win1\_2341 on genome browser  
 win3\_2344 26724655 26724662 seq\_19 844094 844100 See win3\_2344 on genome browser  
 win3\_2348 26728989 26728996 seq\_19 848428 848434 See win3\_2348 on genome browser  
 win2\_2352 26751829 26751836 seq\_19 871268 871274 See win2\_2352 on genome browser  
 win2\_2356 26784459 26784466 seq\_3706 16299 16305 See win2\_2356 on genome browser  
 win2\_2360 26812837 26812844 seq\_3706 44677 44683 See win2\_2360 on genome browser  
 win2\_2364 26866189 26866196 seq\_3706 98029 98035 See win2\_2364 on genome browser  
 win2\_2368 27005815 27005822 seq\_3706 237655 237661 See win2\_2368 on genome browser  
 win2\_2372 27020750 27020757 seq\_3706 252590 252596 See win2\_2372 on genome browser  
 win3\_2376 27048179 27048186 seq\_3706 280019 280025 See win3\_2376 on genome browser  
 win2\_2380 27075771 27075778 seq\_3706 307611 307617 See win2\_2380 on genome browser  
 win2\_2384 27089244 27089251 seq\_3706 321084 321090 See win2\_2384 on genome browser  
 win2\_2388 27105092 27105099 seq\_3706 336932 336938 See win2\_2388 on genome browser  
 win1\_2392 27167645 27167652 seq\_3706 399485 399491 See win1\_2392 on genome browser  
 win3\_2396 27182575 27182582 seq\_3706 414415 414421 See win3\_2396 on genome browser  
 win3\_2400 27214280 27214287 seq\_3706 446120 446126 See win3\_2400 on genome browser  
 win3\_2404 27219448 27219455 seq\_3706 451288 451294 See win3\_2404 on genome browser  
 win2\_2408 27293329 27293336 seq\_3706 525169 525175 See win2\_2408 on genome browser  
 win3\_2412 27299615 27299622 seq\_3706 531455 531461 See win3\_2412 on genome browser  
 win3\_2416 27385499 27385506 seq\_3706 617339 617345 See win3\_2416 on genome browser  
 win3\_2420 27557130 27557137 seq\_3706 788970 788976 See win3\_2420 on genome browser  
 win3\_2424 27559157 27559164 seq\_3706 790997 791003 See win3\_2424 on genome browser  
 win3\_2428 27634810 27634817 seq\_3706 866650 866656 See win3\_2428 on genome browser  
 win2\_2433 27696292 27696299 seq\_3813 40862 40868 See win2\_2433 on genome browser  
 win1\_2437 27739585 27739591 seq\_3813 84155 84160 See win1\_2437 on genome browser  
 win2\_2440 27760353 27760360 seq\_3813 104923 104929 See win2\_2440 on genome browser  
 win2\_2444 27814245 27814252 seq\_3813 158815 158821 See win2\_2444 on genome browser  
 win3\_2448 27857341 27857348 seq\_3813 201911 201917 See win3\_2448 on genome browser  
 win2\_2452 27880505 27880512 seq\_3813 225075 225081 See win2\_2452 on genome browser  
 win3\_2456 27948084 27948091 seq\_3813 292654 292660 See win3\_2456 on genome browser  
 win2\_2460 28000003 28000010 seq\_3813 344573 344579 See win2\_2460 on genome browser  
 win2\_2464 28052798 28052805 seq\_3813 397368 397374 See win2\_2464 on genome browser  
 win1\_2468 28157328 28157334 seq\_3813 501898 501903 See win1\_2468 on genome browser  
 win2\_2471 28170077 28170084 seq\_3813 514647 514653 See win2\_2471 on genome browser  
 win2\_2475 28262607 28262614 seq\_3813 607177 607183 See win2\_2475 on genome browser  
 win3\_2479 28300629 28300636 seq\_3813 645199 645205 See win3\_2479 on genome browser  
 win3\_2483 28403208 28403215 seq\_3813 747778 747784 See win3\_2483 on genome browser  
 win3\_2487 28423068 28423075 seq\_3813 767638 767644 See win3\_2487 on genome browser  
 win3\_2491 28441418 28441425 seq\_3813 785988 785994 See win3\_2491 on genome browser  
 win3\_2495 28453683 28453690 seq\_3813 798253 798259 See win3\_2495 on genome browser  
 win2\_2499 28455311 28455318 seq\_3813 799881 799887 See win2\_2499 on genome browser  
 win1\_2503 28459429 28459434 seq\_3813 803999 804003 See win1\_2503 on genome browser  
 win2\_2505 28463372 28463379 seq\_3813 807942 807948 See win2\_2505 on genome browser  
 win1\_2509 28490646 28490650 seq\_3813 835216 835219 See win1\_2509 on genome browser  
 win1\_2510 28491802 28491809 seq\_3813 836372 836378 See win1\_2510 on genome browser  
 win3\_2514 28493242 28493249 seq\_3813 837812 837818 See win3\_2514 on genome browser  
 win2\_2518 28493387 28493394 seq\_3813 837957 837963 See win2\_2518 on genome browser  
 win2\_2522 28497776 28497783 seq\_3813 842346 842352 See win2\_2522 on genome browser  
 win3\_2526 28644164 28644171 seq\_21 118113 118119 See win3\_2526 on genome browser  
 win2\_2530 28659060 28659067 seq\_21 133009 133015 See win2\_2530 on genome browser  
 win3\_2534 28683837 28683844 seq\_21 157786 157792 See win3\_2534 on genome browser  
 win2\_2538 28712028 28712035 seq\_21 185977 185983 See win2\_2538 on genome browser  
 win3\_2542 28800020 28800027 seq\_21 273969 273975 See win3\_2542 on genome browser  
 win2\_2546 28852087 28852094 seq\_21 326036 326042 See win2\_2546 on genome browser  
 win3\_2550 28855274 28855281 seq\_21 329223 329229 See win3\_2550 on genome browser  
 win2\_2554 28858151 28858158 seq\_21 332100 332106 See win2\_2554 on genome browser  
 win2\_2558 28897042 28897049 seq\_21 370991 370997 See win2\_2558 on genome browser  
 win3\_2562 28957392 28957399 seq\_21 431341 431347 See win3\_2562 on genome browser  
 win3\_2566 29119243 29119250 seq\_21 593192 593198 See win3\_2566 on genome browser  
 win3\_2570 29173791 29173798 seq\_21 647740 647746 See win3\_2570 on genome browser  
 win3\_2574 29275511 29275518 seq\_21 749460 749466 See win3\_2574 on genome browser  
 win1\_2578 29288349 29288353 seq\_21 762298 762301 See win1\_2578 on genome browser  
 win2\_2579 29337067 29337074 seq\_21 811016 811022 See win2\_2579 on genome browser  
 win3\_2583 29386187 29386194 seq\_22 10039 10045 See win3\_2583 on genome browser  
 win2\_2587 29394899 29394906 seq\_22 18751 18757 See win2\_2587 on genome browser  
 win2\_2591 29433718 29433725 seq\_22 57570 57576 See win2\_2591 on genome browser  
 win2\_2595 29448667 29448674 seq\_22 72519 72525 See win2\_2595 on genome browser  
 win2\_2599 29462753 29462760 seq\_22 86605 86611 See win2\_2599 on genome browser  
 win3\_2603 29467236 29467243 seq\_22 91088 91094 See win3\_2603 on genome browser  
 win2\_2607 29476796 29476803 seq\_22 100648 100654 See win2\_2607 on genome browser  
 win2\_2611 29531635 29531642 seq\_22 155487 155493 See win2\_2611 on genome browser  
 win2\_2615 29585445 29585452 seq\_22 209297 209303 See win2\_2615 on genome browser  
 win3\_2619 29598813 29598820 seq\_22 222665 222671 See win3\_2619 on genome browser  
 win3\_2623 29606249 29606256 seq\_22 230101 230107 See win3\_2623 on genome browser  
 win2\_2627 29607909 29607916 seq\_22 231761 231767 See win2\_2627 on genome browser  
 win2\_2631 29621038 29621045 seq\_22 244890 244896 See win2\_2631 on genome browser  
 win3\_2635 29631187 29631194 seq\_22 255039 255045 See win3\_2635 on genome browser  
 win3\_2639 29638790 29638797 seq\_22 262642 262648 See win3\_2639 on genome browser  
 win3\_2643 29661290 29661297 seq\_22 285142 285148 See win3\_2643 on genome browser  
 win1\_2647 29663024 29663028 seq\_22 286876 286879 See win1\_2647 on genome browser  
 win1\_2648 29664794 29664801 seq\_22 288646 288652 See win1\_2648 on genome browser  
 win3\_2652 29667643 29667650 seq\_22 291495 291501 See win3\_2652 on genome browser  
 win1\_2656 29673142 29673149 seq\_22 296994 297000 See win1\_2656 on genome browser  
 win3\_2660 29751959 29751966 seq\_22 375811 375817 See win3\_2660 on genome browser  
 win3\_2664 29771647 29771654 seq\_22 395499 395505 See win3\_2664 on genome browser  
 win2\_2668 29794765 29794772 seq\_22 418617 418623 See win2\_2668 on genome browser  
 win3\_2672 29794865 29794872 seq\_22 418717 418723 See win3\_2672 on genome browser  
 win2\_2676 29819109 29819116 seq\_22 442961 442967 See win2\_2676 on genome browser  
 win2\_2680 29910116 29910123 seq\_22 533968 533974 See win2\_2680 on genome browser  
 win3\_2684 29949747 29949754 seq\_22 573599 573605 See win3\_2684 on genome browser  
 win2\_2688 30078630 30078637 seq\_22 702482 702488 See win2\_2688 on genome browser  
 win2\_2692 30154046 30154053 seq\_22 777898 777904 See win2\_2692 on genome browser  
 win3\_2696 30161228 30161235 seq\_22 785080 785086 See win3\_2696 on genome browser  
 win3\_2700 30227720 30227727 seq\_23 3210 3216 See win3\_2700 on genome browser  
 win2\_2704 30240957 30240964 seq\_23 16447 16453 See win2\_2704 on genome browser  
 win3\_2708 30253060 30253067 seq\_23 28550 28556 See win3\_2708 on genome browser  
 win3\_2712 30330970 30330977 seq\_23 106460 106466 See win3\_2712 on genome browser  
 win2\_2716 30372271 30372278 seq\_23 147761 147767 See win2\_2716 on genome browser  
 win2\_2720 30440581 30440588 seq\_23 216071 216077 See win2\_2720 on genome browser  
 win3\_2724 30470863 30470870 seq\_23 246353 246359 See win3\_2724 on genome browser  
 win2\_2728 30519586 30519593 seq\_23 295076 295082 See win2\_2728 on genome browser  
 win3\_2732 30523328 30523335 seq\_23 298818 298824 See win3\_2732 on genome browser  
 win1\_2736 30527977 30527983 seq\_23 303467 303472 See win1\_2736 on genome browser  
 win1\_2739 30553071 30553078 seq\_23 328561 328567 See win1\_2739 on genome browser  
 win1\_2743 30571852 30571859 seq\_23 347342 347348 See win1\_2743 on genome browser  
 win3\_2747 30610788 30610795 seq\_23 386278 386284 See win3\_2747 on genome browser  
 win1\_2751 30695812 30695818 seq\_23 471302 471307 See win1\_2751 on genome browser  
 win3\_2754 30860423 30860430 seq\_23 635913 635919 See win3\_2754 on genome browser  
 win3\_2758 30860534 30860541 seq\_23 636024 636030 See win3\_2758 on genome browser  
 win2\_2762 30864899 30864906 seq\_23 640389 640395 See win2\_2762 on genome browser  
 win3\_2766 30918649 30918656 seq\_23 694139 694145 See win3\_2766 on genome browser  
 win3\_2770 30949084 30949091 seq\_23 724574 724580 See win3\_2770 on genome browser  
 win1\_2774 30978618 30978624 seq\_23 754108 754113 See win1\_2774 on genome browser  
 win2\_2777 31197870 31197877 seq\_3690 142077 142083 See win2\_2777 on genome browser  
 win3\_2781 31222539 31222546 seq\_3690 166746 166752 See win3\_2781 on genome browser  
 win2\_2785 31271320 31271327 seq\_3690 215527 215533 See win2\_2785 on genome browser  
 win3\_2789 31316427 31316434 seq\_3690 260634 260640 See win3\_2789 on genome browser  
 win3\_2793 31325120 31325127 seq\_3690 269327 269333 See win3\_2793 on genome browser  
 win2\_2797 31377989 31377996 seq\_3690 322196 322202 See win2\_2797 on genome browser  
 win2\_2801 31438932 31438939 seq\_3690 383139 383145 See win2\_2801 on genome browser  
 win2\_2805 31466793 31466800 seq\_3690 411000 411006 See win2\_2805 on genome browser  
 win3\_2809 31487574 31487581 seq\_3690 431781 431787 See win3\_2809 on genome browser  
 win2\_2813 31495591 31495598 seq\_3690 439798 439804 See win2\_2813 on genome browser  
 win1\_2817 31506303 31506308 seq\_3690 450510 450514 See win1\_2817 on genome browser  
 win3\_2819 31563508 31563515 seq\_3690 507715 507721 See win3\_2819 on genome browser  
 win3\_2823 31590336 31590343 seq\_3690 534543 534549 See win3\_2823 on genome browser  
 win2\_2827 31627274 31627281 seq\_3690 571481 571487 See win2\_2827 on genome browser  
 win3\_2831 31650375 31650382 seq\_3690 594582 594588 See win3\_2831 on genome browser  
 win3\_2835 31660322 31660329 seq\_3690 604529 604535 See win3\_2835 on genome browser  
 win2\_2839 31662735 31662742 seq\_3690 606942 606948 See win2\_2839 on genome browser  
 win3\_2843 31677773 31677780 seq\_3690 621980 621986 See win3\_2843 on genome browser  
 win3\_2847 31749611 31749618 seq\_3690 693818 693824 See win3\_2847 on genome browser  
 win2\_2851 31790107 31790114 seq\_3690 734314 734320 See win2\_2851 on genome browser  
 win3\_2855 31867799 31867806 seq\_3690 812006 812012 See win3\_2855 on genome browser  
 win3\_2859 31954677 31954684 seq\_3694 69381 69387 See win3\_2859 on genome browser  
 win3\_2863 31986908 31986915 seq\_3694 101612 101618 See win3\_2863 on genome browser  
 win3\_2867 31995598 31995605 seq\_3694 110302 110308 See win3\_2867 on genome browser  
 win2\_2871 32054127 32054134 seq\_3694 168831 168837 See win2\_2871 on genome browser  
 win3\_2875 32056428 32056435 seq\_3694 171132 171138 See win3\_2875 on genome browser  
 win2\_2879 32095665 32095672 seq\_3694 210369 210375 See win2\_2879 on genome browser  
 win2\_2883 32097186 32097193 seq\_3694 211890 211896 See win2\_2883 on genome browser  
 win3\_2887 32101719 32101726 seq\_3694 216423 216429 See win3\_2887 on genome browser  
 win2\_2891 32107598 32107605 seq\_3694 222302 222308 See win2\_2891 on genome browser  
 win2\_2895 32207050 32207057 seq\_3694 321754 321760 See win2\_2895 on genome browser  
 win2\_2899 32343898 32343905 seq\_3694 458602 458608 See win2\_2899 on genome browser  
 win1\_2903 32345262 32345267 seq\_3694 459966 459970 See win1\_2903 on genome browser  
 win2\_2905 32365524 32365531 seq\_3694 480228 480234 See win2\_2905 on genome browser  
 win3\_2909 32385475 32385482 seq\_3694 500179 500185 See win3\_2909 on genome browser  
 win2\_2913 32385548 32385555 seq\_3694 500252 500258 See win2\_2913 on genome browser  
 win3\_2917 32446907 32446914 seq\_3694 561611 561617 See win3\_2917 on genome browser  
 win2\_2921 32463477 32463484 seq\_3694 578181 578187 See win2\_2921 on genome browser  
 win3\_2925 32570335 32570342 seq\_3694 685039 685045 See win3\_2925 on genome browser  
 win3\_2929 32587474 32587481 seq\_3694 702178 702184 See win3\_2929 on genome browser  
 win1\_2933 32596424 32596428 seq\_3694 711128 711131 See win1\_2933 on genome browser  
 win3\_2934 32676915 32676922 seq\_3694 791619 791625 See win3\_2934 on genome browser  
 win3\_2938 32924194 32924201 seq\_3833 212838 212844 See win3\_2938 on genome browser  
 win3\_2942 32961820 32961827 seq\_3833 250464 250470 See win3\_2942 on genome browser  
 win2\_2946 32986595 32986602 seq\_3833 275239 275245 See win2\_2946 on genome browser  
 win2\_2950 33027849 33027856 seq\_3833 316493 316499 See win2\_2950 on genome browser  
 win2\_2954 33120239 33120246 seq\_3833 408883 408889 See win2\_2954 on genome browser  
 win3\_2958 33124508 33124515 seq\_3833 413152 413158 See win3\_2958 on genome browser  
 win3\_2962 33149131 33149138 seq\_3833 437775 437781 See win3\_2962 on genome browser  
 win2\_2966 33185138 33185145 seq\_3833 473782 473788 See win2\_2966 on genome browser  
 win3\_2970 33190295 33190302 seq\_3833 478939 478945 See win3\_2970 on genome browser  
 win2\_2974 33193829 33193836 seq\_3833 482473 482479 See win2\_2974 on genome browser  
 win1\_2978 33202566 33202571 seq\_3833 491210 491214 See win1\_2978 on genome browser  
 win3\_2980 33296690 33296697 seq\_3833 585334 585340 See win3\_2980 on genome browser  
 win3\_2984 33296820 33296827 seq\_3833 585464 585470 See win3\_2984 on genome browser  
 win2\_2988 33460561 33460568 seq\_3833 749205 749211 See win2\_2988 on genome browser  
 win2\_2992 33605120 33605127 seq\_3688 72285 72291 See win2\_2992 on genome browser  
 win2\_2996 33618652 33618659 seq\_3688 85817 85823 See win2\_2996 on genome browser  
 win2\_3000 33779947 33779954 seq\_3688 247112 247118 See win2\_3000 on genome browser  
 win3\_3004 33876279 33876286 seq\_3688 343444 343450 See win3\_3004 on genome browser  
 win2\_3008 33949875 33949882 seq\_3688 417040 417046 See win2\_3008 on genome browser  
 win3\_3012 33954614 33954621 seq\_3688 421779 421785 See win3\_3012 on genome browser  
 win3\_3016 34037220 34037227 seq\_3688 504385 504391 See win3\_3016 on genome browser  
 win2\_3020 34040650 34040657 seq\_3688 507815 507821 See win2\_3020 on genome browser  
 win3\_3024 34043189 34043196 seq\_3688 510354 510360 See win3\_3024 on genome browser  
 win3\_3028 34049870 34049877 seq\_3688 517035 517041 See win3\_3028 on genome browser  
 win2\_3032 34054870 34054877 seq\_3688 522035 522041 See win2\_3032 on genome browser  
 win3\_3036 34066027 34066034 seq\_3688 533192 533198 See win3\_3036 on genome browser  
 win3\_3040 34067079 34067086 seq\_3688 534244 534250 See win3\_3040 on genome browser  
 win3\_3044 34070493 34070500 seq\_3688 537658 537664 See win3\_3044 on genome browser  
 win3\_3048 34076430 34076437 seq\_3688 543595 543601 See win3\_3048 on genome browser  
 win3\_3052 34084859 34084866 seq\_3688 552024 552030 See win3\_3052 on genome browser  
 win3\_3056 34210322 34210329 seq\_3688 677487 677493 See win3\_3056 on genome browser  
 win2\_3060 34253446 34253453 seq\_3688 720611 720617 See win2\_3060 on genome browser  
 win2\_3064 34360976 34360983 seq\_3691 55874 55880 See win2\_3064 on genome browser  
 win3\_3068 34410008 34410015 seq\_3691 104906 104912 See win3\_3068 on genome browser  
 win2\_3072 34422510 34422517 seq\_3691 117408 117414 See win2\_3072 on genome browser  
 win2\_3076 34425316 34425323 seq\_3691 120214 120220 See win2\_3076 on genome browser  
 win1\_3080 34757934 34757941 seq\_3691 452832 452838 See win1\_3080 on genome browser  
 win2\_3084 34819253 34819260 seq\_3691 514151 514157 See win2\_3084 on genome browser  
 win3\_3088 35003297 35003304 seq\_3691 698195 698201 See win3\_3088 on genome browser  
 win2\_3092 35061055 35061062 seq\_3691 755953 755959 See win2\_3092 on genome browser  
 win3\_3096 35074562 35074569 seq\_3692 26 32 See win3\_3096 on genome browser  
 win3\_3100 35392173 35392180 seq\_3692 317637 317643 See win3\_3100 on genome browser  
 win3\_3104 35828158 35828165 seq\_3692 753622 753628 See win3\_3104 on genome browser  
 win2\_3108 35926058 35926065 seq\_3827 83221 83227 See win2\_3108 on genome browser  
 win2\_3112 36000097 36000104 seq\_3827 157260 157266 See win2\_3112 on genome browser  
 win3\_3116 36026493 36026500 seq\_3827 183656 183662 See win3\_3116 on genome browser  
 win3\_3120 36040409 36040416 seq\_3827 197572 197578 See win3\_3120 on genome browser  
 win1\_3124 36055783 36055788 seq\_3827 212946 212950 See win1\_3124 on genome browser  
 win2\_3126 36060966 36060973 seq\_3827 218129 218135 See win2\_3126 on genome browser  
 win3\_3130 36062202 36062209 seq\_3827 219365 219371 See win3\_3130 on genome browser  
 win3\_3134 36068299 36068306 seq\_3827 225462 225468 See win3\_3134 on genome browser  
 win3\_3138 36091445 36091452 seq\_3827 248608 248614 See win3\_3138 on genome browser  
 win3\_3142 36148630 36148637 seq\_3827 305793 305799 See win3\_3142 on genome browser  
 win3\_3146 36178104 36178111 seq\_3827 335267 335273 See win3\_3146 on genome browser  
 win2\_3150 36196611 36196618 seq\_3827 353774 353780 See win2\_3150 on genome browser  
 win3\_3154 36218397 36218404 seq\_3827 375560 375566 See win3\_3154 on genome browser  
 win1\_3158 36251480 36251485 seq\_3827 408643 408647 See win1\_3158 on genome browser  
 win3\_3160 36252802 36252809 seq\_3827 409965 409971 See win3\_3160 on genome browser  
 win3\_3164 36266672 36266679 seq\_3827 423835 423841 See win3\_3164 on genome browser  
 win2\_3168 36300429 36300436 seq\_3827 457592 457598 See win2\_3168 on genome browser  
 win1\_3172 36347764 36347771 seq\_3827 504927 504933 See win1\_3172 on genome browser  
 win2\_3176 36423353 36423360 seq\_3827 580516 580522 See win2\_3176 on genome browser  
 win3\_3180 36514836 36514843 seq\_3827 671999 672005 See win3\_3180 on genome browser  
 win3\_3184 36527637 36527644 seq\_3827 684800 684806 See win3\_3184 on genome browser  
 win3\_3188 36527771 36527778 seq\_3827 684934 684940 See win3\_3188 on genome browser  
 win3\_3192 36541880 36541887 seq\_3827 699043 699049 See win3\_3192 on genome browser  
 win2\_3196 36542483 36542490 seq\_3827 699646 699652 See win2\_3196 on genome browser  
 win3\_3200 36555376 36555383 seq\_3827 712539 712545 See win3\_3200 on genome browser  
 win3\_3204 36564809 36564816 seq\_3827 721972 721978 See win3\_3204 on genome browser  
 win2\_3208 36583859 36583866 seq\_3687 7086 7092 See win2\_3208 on genome browser  
 win3\_3212 36674212 36674219 seq\_3687 97439 97445 See win3\_3212 on genome browser  
 win2\_3216 36795176 36795183 seq\_3687 218403 218409 See win2\_3216 on genome browser  
 win3\_3220 37070911 37070918 seq\_3687 494138 494144 See win3\_3220 on genome browser  
 win3\_3224 37157443 37157450 seq\_3687 580670 580676 See win3\_3224 on genome browser  
 win2\_3228 37166673 37166680 seq\_3687 589900 589906 See win2\_3228 on genome browser  
 win3\_3232 37254453 37254460 seq\_3687 677680 677686 See win3\_3232 on genome browser  
 win3\_3236 37320404 37320411 seq\_3696 17994 18000 See win3\_3236 on genome browser  
 win3\_3240 37352721 37352728 seq\_3696 50311 50317 See win3\_3240 on genome browser  
 win3\_3244 37382010 37382017 seq\_3696 79600 79606 See win3\_3244 on genome browser  
 win3\_3248 37402529 37402536 seq\_3696 100119 100125 See win3\_3248 on genome browser  
 win2\_3252 37503127 37503134 seq\_3696 200717 200723 See win2\_3252 on genome browser  
 win3\_3256 37505405 37505412 seq\_3696 202995 203001 See win3\_3256 on genome browser  
 win2\_3260 37528666 37528673 seq\_3696 226256 226262 See win2\_3260 on genome browser  
 win3\_3264 37528673 37528680 seq\_3696 226263 226269 See win3\_3264 on genome browser  
 win3\_3268 37555228 37555235 seq\_3696 252818 252824 See win3\_3268 on genome browser  
 win2\_3272 37596223 37596230 seq\_3696 293813 293819 See win2\_3272 on genome browser  
 win2\_3276 37666623 37666630 seq\_3696 364213 364219 See win2\_3276 on genome browser  
 win2\_3280 37671051 37671058 seq\_3696 368641 368647 See win2\_3280 on genome browser  
 win2\_3284 37682916 37682923 seq\_3696 380506 380512 See win2\_3284 on genome browser  
 win3\_3288 37719511 37719516 seq\_3696 417101 417105 See win3\_3288 on genome browser  
 win3\_3290 37780657 37780664 seq\_3696 478247 478253 See win3\_3290 on genome browser  
 win2\_3294 37782420 37782427 seq\_3696 480010 480016 See win2\_3294 on genome browser  
 win3\_3298 37785792 37785799 seq\_3696 483382 483388 See win3\_3298 on genome browser  
 win3\_3302 37789958 37789965 seq\_3696 487548 487554 See win3\_3302 on genome browser  
 win3\_3306 37804814 37804821 seq\_3696 502404 502410 See win3\_3306 on genome browser  
 win2\_3310 37821411 37821418 seq\_3696 519001 519007 See win2\_3310 on genome browser  
 win2\_3314 37979276 37979283 seq\_3696 676866 676872 See win2\_3314 on genome browser  
 win2\_3318 37981906 37981913 seq\_3696 679496 679502 See win2\_3318 on genome browser  
 win3\_3322 38084437 38084444 seq\_32 57751 57757 See win3\_3322 on genome browser  
 win2\_3326 38087565 38087572 seq\_32 60879 60885 See win2\_3326 on genome browser  
 win2\_3330 38128080 38128087 seq\_32 101394 101400 See win2\_3330 on genome browser  
 win1\_3334 38138633 38138637 seq\_32 111947 111950 See win1\_3334 on genome browser  
 win3\_3335 38152864 38152871 seq\_32 126178 126184 See win3\_3335 on genome browser  
 win3\_3339 38231597 38231604 seq\_32 204911 204917 See win3\_3339 on genome browser  
 win3\_3343 38234355 38234362 seq\_32 207669 207675 See win3\_3343 on genome browser  
 win3\_3347 38255893 38255900 seq\_32 229207 229213 See win3\_3347 on genome browser  
 win2\_3351 38317296 38317303 seq\_32 290610 290616 See win2\_3351 on genome browser  
 win2\_3355 38423222 38423229 seq\_32 396536 396542 See win2\_3355 on genome browser  
 win2\_3359 38499080 38499087 seq\_32 472394 472400 See win2\_3359 on genome browser  
 win1\_3363 38504445 38504449 seq\_32 477759 477762 See win1\_3363 on genome browser  
 win2\_3364 38518213 38518220 seq\_32 491527 491533 See win2\_3364 on genome browser  
 win3\_3368 38624912 38624919 seq\_32 598226 598232 See win3\_3368 on genome browser  
 win3\_3372 38696577 38696584 seq\_32 669891 669897 See win3\_3372 on genome browser  
 win1\_3376 38749946 38749953 seq\_3705 27096 27102 See win1\_3376 on genome browser  
 win3\_3380 38769093 38769100 seq\_3705 46243 46249 See win3\_3380 on genome browser  
 win3\_3384 38819418 38819425 seq\_3705 96568 96574 See win3\_3384 on genome browser  
 win2\_3388 39108658 39108665 seq\_3705 385808 385814 See win2\_3388 on genome browser  
 win2\_3392 39236421 39236428 seq\_3705 513571 513577 See win2\_3392 on genome browser  
 win2\_3396 39249557 39249564 seq\_3705 526707 526713 See win2\_3396 on genome browser  
 win3\_3400 39260371 39260378 seq\_3705 537521 537527 See win3\_3400 on genome browser  
 win2\_3404 39313737 39313744 seq\_3705 590887 590893 See win2\_3404 on genome browser  
 win3\_3408 39320685 39320692 seq\_3705 597835 597841 See win3\_3408 on genome browser  
 win2\_3412 39333796 39333803 seq\_3705 610946 610952 See win2\_3412 on genome browser  
 win1\_3416 39345847 39345852 seq\_3705 622997 623001 See win1\_3416 on genome browser  
 win2\_3418 39372526 39372533 seq\_3705 649676 649682 See win2\_3418 on genome browser  
 win3\_3422 39439793 39439800 seq\_3823 23066 23072 See win3\_3422 on genome browser  
 win3\_3426 39487070 39487077 seq\_3823 70343 70349 See win3\_3426 on genome browser  
 win2\_3430 39525947 39525954 seq\_3823 109220 109226 See win2\_3430 on genome browser  
 win3\_3434 39551623 39551630 seq\_3823 134896 134902 See win3\_3434 on genome browser  
 win1\_3438 39580608 39580613 seq\_3823 163881 163885 See win1\_3438 on genome browser  
 win3\_3440 39585779 39585786 seq\_3823 169052 169058 See win3\_3440 on genome browser  
 win3\_3444 39623735 39623742 seq\_3823 207008 207014 See win3\_3444 on genome browser  
 win1\_3448 39714584 39714591 seq\_3823 297857 297863 See win1\_3448 on genome browser  
 win3\_3452 39768110 39768117 seq\_3823 351383 351389 See win3\_3452 on genome browser  
 win2\_3456 39776818 39776825 seq\_3823 360091 360097 See win2\_3456 on genome browser  
 win3\_3460 39811189 39811196 seq\_3823 394462 394468 See win3\_3460 on genome browser  
 win2\_3464 39811274 39811281 seq\_3823 394547 394553 See win2\_3464 on genome browser  
 win2\_3468 39833624 39833631 seq\_3823 416897 416903 See win2\_3468 on genome browser  
 win1\_3472 39878476 39878482 seq\_3823 461749 461754 See win1\_3472 on genome browser  
 win3\_3475 39881046 39881053 seq\_3823 464319 464325 See win3\_3475 on genome browser  
 win3\_3479 40029355 40029362 seq\_3823 612628 612634 See win3\_3479 on genome browser  
 win2\_3483 40049195 40049202 seq\_3823 632468 632474 See win2\_3483 on genome browser  
 win3\_3487 40107363 40107370 seq\_3689 665 671 See win3\_3487 on genome browser  
 win1\_3491 40150203 40150210 seq\_3689 43505 43511 See win1\_3491 on genome browser  
 win2\_3495 40277848 40277855 seq\_3689 171150 171156 See win2\_3495 on genome browser  
 win2\_3499 40394756 40394763 seq\_3689 288058 288064 See win2\_3499 on genome browser  
 win3\_3503 40518179 40518186 seq\_3689 411481 411487 See win3\_3503 on genome browser  
 win3\_3507 40701834 40701841 seq\_3689 595136 595142 See win3\_3507 on genome browser  
 win2\_3511 40787046 40787053 seq\_3695 6470 6476 See win2\_3511 on genome browser  
 win1\_3515 40867968 40867974 seq\_3695 87392 87397 See win1\_3515 on genome browser  
 win3\_3518 40901303 40901310 seq\_3695 120727 120733 See win3\_3518 on genome browser  
 win3\_3522 40917730 40917737 seq\_3695 137154 137160 See win3\_3522 on genome browser  
 win2\_3526 40985969 40985976 seq\_3695 205393 205399 See win2\_3526 on genome browser  
 win3\_3530 40994505 40994512 seq\_3695 213929 213935 See win3\_3530 on genome browser  
 win3\_3534 41003556 41003563 seq\_3695 222980 222986 See win3\_3534 on genome browser  
 win1\_3538 41027522 41027529 seq\_3695 246946 246952 See win1\_3538 on genome browser  
 win3\_3542 41044451 41044458 seq\_3695 263875 263881 See win3\_3542 on genome browser  
 win1\_3546 41057005 41057012 seq\_3695 276429 276435 See win1\_3546 on genome browser  
 win3\_3550 41079656 41079663 seq\_3695 299080 299086 See win3\_3550 on genome browser  
 win3\_3554 41086332 41086339 seq\_3695 305756 305762 See win3\_3554 on genome browser  
 win2\_3558 41089132 41089139 seq\_3695 308556 308562 See win2\_3558 on genome browser  
 win3\_3562 41102427 41102434 seq\_3695 321851 321857 See win3\_3562 on genome browser  
 win2\_3566 41132912 41132919 seq\_3695 352336 352342 See win2\_3566 on genome browser  
 win3\_3570 41205516 41205523 seq\_3695 424940 424946 See win3\_3570 on genome browser  
 win2\_3574 41212654 41212661 seq\_3695 432078 432084 See win2\_3574 on genome browser  
 win2\_3578 41217248 41217253 seq\_3695 436672 436676 See win2\_3578 on genome browser  
 win3\_3580 41217317 41217322 seq\_3695 436741 436745 See win3\_3580 on genome browser  
 win2\_3582 41217379 41217386 seq\_3695 436803 436809 See win2\_3582 on genome browser  
 win3\_3586 41229426 41229433 seq\_3695 448850 448856 See win3\_3586 on genome browser  
 win2\_3590 41285266 41285273 seq\_3695 504690 504696 See win2\_3590 on genome browser  
 win2\_3594 41297730 41297737 seq\_3695 517154 517160 See win2\_3594 on genome browser  
 win3\_3598 41301794 41301801 seq\_3695 521218 521224 See win3\_3598 on genome browser  
 win2\_3602 41416230 41416237 seq\_3695 635654 635660 See win2\_3602 on genome browser  
 win3\_3606 41424811 41424818 seq\_3695 644235 644241 See win3\_3606 on genome browser  
 win3\_3610 41538061 41538068 seq\_3703 100997 101003 See win3\_3610 on genome browser  
 win3\_3614 41612026 41612033 seq\_3703 174962 174968 See win3\_3614 on genome browser  
 win2\_3618 41684977 41684984 seq\_3703 247913 247919 See win2\_3618 on genome browser  
 win3\_3622 41713594 41713601 seq\_3703 276530 276536 See win3\_3622 on genome browser  
 win2\_3626 41762190 41762197 seq\_3703 325126 325132 See win2\_3626 on genome browser  
 win2\_3630 41849408 41849415 seq\_3703 412344 412350 See win2\_3630 on genome browser  
 win3\_3634 41882499 41882506 seq\_3703 445435 445441 See win3\_3634 on genome browser  
 win2\_3638 41882599 41882606 seq\_3703 445535 445541 See win2\_3638 on genome browser  
 win3\_3642 41944480 41944487 seq\_3703 507416 507422 See win3\_3642 on genome browser  
 win3\_3646 41961790 41961797 seq\_3703 524726 524732 See win3\_3646 on genome browser  
 win3\_3650 42091793 42091800 seq\_38 5963 5969 See win3\_3650 on genome browser  
 win2\_3654 42101552 42101559 seq\_38 15722 15728 See win2\_3654 on genome browser  
 win1\_3658 42109183 42109189 seq\_38 23353 23358 See win1\_3658 on genome browser  
 win2\_3661 42111456 42111463 seq\_38 25626 25632 See win2\_3661 on genome browser  
 win3\_3665 42217134 42217141 seq\_38 131304 131310 See win3\_3665 on genome browser  
 win2\_3669 42262111 42262118 seq\_38 176281 176287 See win2\_3669 on genome browser  
 win2\_3673 42289587 42289594 seq\_38 203757 203763 See win2\_3673 on genome browser  
 win1\_3677 42290798 42290803 seq\_38 204968 204972 See win1\_3677 on genome browser  
 win3\_3679 42297465 42297472 seq\_38 211635 211641 See win3\_3679 on genome browser  
 win1\_3683 42299040 42299044 seq\_38 213210 213213 See win1\_3683 on genome browser  
 win2\_3684 42300018 42300025 seq\_38 214188 214194 See win2\_3684 on genome browser  
 win2\_3688 42301168 42301175 seq\_38 215338 215344 See win2\_3688 on genome browser  
 win3\_3692 42304836 42304843 seq\_38 219006 219012 See win3\_3692 on genome browser  
 win3\_3696 42304901 42304908 seq\_38 219071 219077 See win3\_3696 on genome browser  
 win1\_3700 42307081 42307088 seq\_38 221251 221257 See win1\_3700 on genome browser  
 win2\_3704 42337099 42337106 seq\_38 251269 251275 See win2\_3704 on genome browser  
 win2\_3708 42340725 42340732 seq\_38 254895 254901 See win2\_3708 on genome browser  
 win3\_3712 42364233 42364240 seq\_38 278403 278409 See win3\_3712 on genome browser  
 win1\_3716 42370774 42370778 seq\_38 284944 284947 See win1\_3716 on genome browser  
 win1\_3717 42386660 42386666 seq\_38 300830 300835 See win1\_3717 on genome browser  
 win2\_3720 42393494 42393501 seq\_38 307664 307670 See win2\_3720 on genome browser  
 win2\_3724 42405978 42405985 seq\_38 320148 320154 See win2\_3724 on genome browser  
 win2\_3728 42414406 42414413 seq\_38 328576 328582 See win2\_3728 on genome browser  
 win3\_3732 42432237 42432244 seq\_38 346407 346413 See win3\_3732 on genome browser  
 win3\_3736 42453238 42453245 seq\_38 367408 367414 See win3\_3736 on genome browser  
 win2\_3740 42455992 42455999 seq\_38 370162 370168 See win2\_3740 on genome browser  
 win2\_3744 42457426 42457433 seq\_38 371596 371602 See win2\_3744 on genome browser  
 win3\_3748 42524397 42524404 seq\_38 438567 438573 See win3\_3748 on genome browser  
 win3\_3752 42540075 42540082 seq\_38 454245 454251 See win3\_3752 on genome browser  
 win3\_3756 42559295 42559302 seq\_38 473465 473471 See win3\_3756 on genome browser  
 win3\_3760 42589952 42589959 seq\_38 504122 504128 See win3\_3760 on genome browser  
 win1\_3764 42615192 42615196 seq\_38 529362 529365 See win1\_3764 on genome browser  
 win1\_3765 42626971 42626977 seq\_38 541141 541146 See win1\_3765 on genome browser  
 win2\_3768 42629452 42629459 seq\_38 543622 543628 See win2\_3768 on genome browser  
 win3\_3772 42641392 42641399 seq\_38 555562 555568 See win3\_3772 on genome browser  
 win3\_3776 42657561 42657568 seq\_38 571731 571737 See win3\_3776 on genome browser  
 win3\_3780 42705530 42705537 seq\_38 619700 619706 See win3\_3780 on genome browser  
 win3\_3784 42767118 42767125 seq\_3693 48374 48380 See win3\_3784 on genome browser  
 win2\_3788 42920343 42920350 seq\_3693 201599 201605 See win2\_3788 on genome browser  
 win3\_3792 42980008 42980015 seq\_3693 261264 261270 See win3\_3792 on genome browser  
 win2\_3796 43142233 43142240 seq\_3693 423489 423495 See win2\_3796 on genome browser  
 win3\_3800 43175060 43175067 seq\_3693 456316 456322 See win3\_3800 on genome browser  
 win3\_3804 43192810 43192817 seq\_3693 474066 474072 See win3\_3804 on genome browser  
 win2\_3808 43204948 43204955 seq\_3693 486204 486210 See win2\_3808 on genome browser  
 win3\_3812 43208623 43208630 seq\_3693 489879 489885 See win3\_3812 on genome browser  
 win2\_3816 43274240 43274247 seq\_3693 555496 555502 See win2\_3816 on genome browser  
 win3\_3820 43284657 43284664 seq\_3693 565913 565919 See win3\_3820 on genome browser  
 win3\_3824 43297504 43297511 seq\_3693 578760 578766 See win3\_3824 on genome browser  
 win3\_3828 43344897 43344904 seq\_40 2680 2686 See win3\_3828 on genome browser  
 win1\_3832 43354343 43354348 seq\_40 12126 12130 See win1\_3832 on genome browser  
 win1\_3834 43355309 43355315 seq\_40 13092 13097 See win1\_3834 on genome browser  
 win3\_3837 43364170 43364177 seq\_40 21953 21959 See win3\_3837 on genome browser  
 win2\_3841 43448316 43448323 seq\_40 106099 106105 See win2\_3841 on genome browser  
 win2\_3845 43450027 43450034 seq\_40 107810 107816 See win2\_3845 on genome browser  
 win2\_3849 43553821 43553828 seq\_40 211604 211610 See win2\_3849 on genome browser  
 win3\_3853 43577382 43577389 seq\_40 235165 235171 See win3\_3853 on genome browser  
 win3\_3857 43625725 43625731 seq\_40 283508 283513 See win3\_3857 on genome browser  
 win2\_3860 43625782 43625789 seq\_40 283565 283571 See win2\_3860 on genome browser  
 win3\_3864 43658003 43658010 seq\_40 315786 315792 See win3\_3864 on genome browser  
 win2\_3868 43665481 43665488 seq\_40 323264 323270 See win2\_3868 on genome browser  
 win2\_3872 43778519 43778526 seq\_40 436302 436308 See win2\_3872 on genome browser  
 win2\_3876 43783995 43784002 seq\_40 441778 441784 See win2\_3876 on genome browser  
 win3\_3880 43790100 43790107 seq\_40 447883 447889 See win3\_3880 on genome browser  
 win2\_3884 43878954 43878961 seq\_40 536737 536743 See win2\_3884 on genome browser  
 win3\_3888 43906327 43906334 seq\_40 564110 564116 See win3\_3888 on genome browser  
 win3\_3892 43917945 43917952 seq\_40 575728 575734 See win3\_3892 on genome browser  
 win3\_3896 43922636 43922643 seq\_40 580419 580425 See win3\_3896 on genome browser  
 win2\_3900 43924085 43924092 seq\_40 581868 581874 See win2\_3900 on genome browser  
 win3\_3904 43948585 43948592 seq\_40 606368 606374 See win3\_3904 on genome browser  
 win3\_3908 43965673 43965680 seq\_41 2284 2290 See win3\_3908 on genome browser  
 win2\_3912 44061357 44061364 seq\_41 97968 97974 See win2\_3912 on genome browser  
 win3\_3916 44061400 44061407 seq\_41 98011 98017 See win3\_3916 on genome browser  
 win3\_3920 44100465 44100472 seq\_41 137076 137082 See win3\_3920 on genome browser  
 win2\_3924 44101128 44101133 seq\_41 137739 137743 See win2\_3924 on genome browser  
 win2\_3926 44121119 44121126 seq\_41 157730 157736 See win2\_3926 on genome browser  
 win3\_3930 44272001 44272008 seq\_41 308612 308618 See win3\_3930 on genome browser  
 win2\_3934 44370466 44370473 seq\_41 407077 407083 See win2\_3934 on genome browser  
 win3\_3938 44374926 44374933 seq\_41 411537 411543 See win3\_3938 on genome browser  
 win3\_3942 44470639 44470646 seq\_41 507250 507256 See win3\_3942 on genome browser  
 win2\_3946 44480182 44480189 seq\_41 516793 516799 See win2\_3946 on genome browser  
 win3\_3950 44506266 44506273 seq\_41 542877 542883 See win3\_3950 on genome browser  
 win3\_3954 44689911 44689918 seq\_3714 110558 110564 See win3\_3954 on genome browser  
 win2\_3958 44809664 44809671 seq\_3714 230311 230317 See win2\_3958 on genome browser  
 win1\_3962 44814125 44814132 seq\_3714 234772 234778 See win1\_3962 on genome browser  
 win2\_3966 44879607 44879614 seq\_3714 300254 300260 See win2\_3966 on genome browser  
 win3\_3970 45144578 45144585 seq\_3714 565225 565231 See win3\_3970 on genome browser  
 win3\_3974 45260430 45260437 seq\_3713 73636 73642 See win3\_3974 on genome browser  
 win3\_3978 45342433 45342440 seq\_3713 155639 155645 See win3\_3978 on genome browser  
 win3\_3982 45374833 45374840 seq\_3713 188039 188045 See win3\_3982 on genome browser  
 win2\_3986 45374969 45374976 seq\_3713 188175 188181 See win2\_3986 on genome browser  
 win3\_3990 45405483 45405490 seq\_3713 218689 218695 See win3\_3990 on genome browser  
 win3\_3994 45409872 45409879 seq\_3713 223078 223084 See win3\_3994 on genome browser  
 win2\_3998 45410116 45410123 seq\_3713 223322 223328 See win2\_3998 on genome browser  
 win2\_4002 45419926 45419933 seq\_3713 233132 233138 See win2\_4002 on genome browser  
 win3\_4006 45423430 45423437 seq\_3713 236636 236642 See win3\_4006 on genome browser  
 win2\_4010 45430664 45430671 seq\_3713 243870 243876 See win2\_4010 on genome browser  
 win1\_4014 45449971 45449977 seq\_3713 263177 263182 See win1\_4014 on genome browser  
 win2\_4017 45470366 45470373 seq\_3713 283572 283578 See win2\_4017 on genome browser  
 win3\_4021 45506243 45506250 seq\_3713 319449 319455 See win3\_4021 on genome browser  
 win2\_4025 45506372 45506379 seq\_3713 319578 319584 See win2\_4025 on genome browser  
 win3\_4029 45508759 45508766 seq\_3713 321965 321971 See win3\_4029 on genome browser  
 win1\_4033 45624267 45624274 seq\_3713 437473 437479 See win1\_4033 on genome browser  
 win3\_4037 45641127 45641134 seq\_3713 454333 454339 See win3\_4037 on genome browser  
 win2\_4041 45641689 45641696 seq\_3713 454895 454901 See win2\_4041 on genome browser  
 win3\_4045 45664858 45664865 seq\_3713 478064 478070 See win3\_4045 on genome browser  
 win2\_4049 45667418 45667425 seq\_3713 480624 480630 See win2\_4049 on genome browser  
 win3\_4053 45713982 45713989 seq\_3713 527188 527194 See win3\_4053 on genome browser  
 win2\_4057 45744145 45744152 seq\_3713 557351 557357 See win2\_4057 on genome browser  
 win2\_4061 45772540 45772547 seq\_3713 585746 585752 See win2\_4061 on genome browser  
 win3\_4065 45812515 45812522 seq\_3679 19880 19886 See win3\_4065 on genome browser  
 win2\_4069 45859027 45859034 seq\_3679 66392 66398 See win2\_4069 on genome browser  
 win3\_4073 45861655 45861662 seq\_3679 69020 69026 See win3\_4073 on genome browser  
 win3\_4077 45970418 45970425 seq\_3679 177783 177789 See win3\_4077 on genome browser  
 win2\_4081 46041567 46041572 seq\_3679 248932 248936 See win2\_4081 on genome browser  
 win1\_4083 46103960 46103967 seq\_3679 311325 311331 See win1\_4083 on genome browser  
 win2\_4087 46168969 46168976 seq\_3679 376334 376340 See win2\_4087 on genome browser  
 win2\_4091 46249789 46249796 seq\_3679 457154 457160 See win2\_4091 on genome browser  
 win3\_4095 46394952 46394959 seq\_47 3097 3103 See win3\_4095 on genome browser  
 win1\_4099 46442458 46442464 seq\_47 50603 50608 See win1\_4099 on genome browser  
 win2\_4102 46492430 46492437 seq\_47 100575 100581 See win2\_4102 on genome browser  
 win3\_4106 46514935 46514942 seq\_47 123080 123086 See win3\_4106 on genome browser  
 win2\_4110 46530226 46530233 seq\_47 138371 138377 See win2\_4110 on genome browser  
 win2\_4114 46530355 46530362 seq\_47 138500 138506 See win2\_4114 on genome browser  
 win2\_4118 46610673 46610680 seq\_47 218818 218824 See win2\_4118 on genome browser  
 win2\_4122 46610853 46610860 seq\_47 218998 219004 See win2\_4122 on genome browser  
 win3\_4126 46635364 46635371 seq\_47 243509 243515 See win3\_4126 on genome browser  
 win2\_4130 46651462 46651469 seq\_47 259607 259613 See win2\_4130 on genome browser  
 win2\_4134 46742005 46742012 seq\_47 350150 350156 See win2\_4134 on genome browser  
 win3\_4138 46750396 46750403 seq\_47 358541 358547 See win3\_4138 on genome browser  
 win3\_4142 46753439 46753446 seq\_47 361584 361590 See win3\_4142 on genome browser  
 win3\_4146 46771104 46771111 seq\_47 379249 379255 See win3\_4146 on genome browser  
 win2\_4150 46824688 46824695 seq\_47 432833 432839 See win2\_4150 on genome browser  
 win3\_4154 46841131 46841138 seq\_47 449276 449282 See win3\_4154 on genome browser  
 win2\_4158 46876984 46876991 seq\_47 485129 485135 See win2\_4158 on genome browser  
 win2\_4162 46971656 46971663 seq\_47 579801 579807 See win2\_4162 on genome browser  
 win3\_4166 46973159 46973166 seq\_47 581304 581310 See win3\_4166 on genome browser  
 win3\_4170 47016380 47016387 seq\_3708 31504 31510 See win3\_4170 on genome browser  
 win2\_4174 47039028 47039035 seq\_3708 54152 54158 See win2\_4174 on genome browser  
 win2\_4178 47045267 47045274 seq\_3708 60391 60397 See win2\_4178 on genome browser  
 win3\_4182 47145893 47145900 seq\_3708 161017 161023 See win3\_4182 on genome browser  
 win2\_4186 47149101 47149108 seq\_3708 164225 164231 See win2\_4186 on genome browser  
 win3\_4190 47157669 47157676 seq\_3708 172793 172799 See win3\_4190 on genome browser  
 win3\_4194 47248507 47248514 seq\_3708 263631 263637 See win3\_4194 on genome browser  
 win2\_4198 47282444 47282451 seq\_3708 297568 297574 See win2\_4198 on genome browser  
 win2\_4202 47430889 47430896 seq\_3708 446013 446019 See win2\_4202 on genome browser  
 win3\_4206 47493736 47493743 seq\_3708 508860 508866 See win3\_4206 on genome browser  
 win3\_4210 47501593 47501600 seq\_3708 516717 516723 See win3\_4210 on genome browser  
 win2\_4214 47524651 47524658 seq\_3708 539775 539781 See win2\_4214 on genome browser  
 win3\_4218 47528779 47528786 seq\_3708 543903 543909 See win3\_4218 on genome browser  
 win3\_4222 47561862 47561869 seq\_3811 8303 8309 See win3\_4222 on genome browser  
 win2\_4226 47686076 47686083 seq\_3811 132517 132523 See win2\_4226 on genome browser  
 win2\_4230 47779394 47779401 seq\_3811 225835 225841 See win2\_4230 on genome browser  
 win3\_4234 47785568 47785575 seq\_3811 232009 232015 See win3\_4234 on genome browser  
 win2\_4238 47819276 47819283 seq\_3811 265717 265723 See win2\_4238 on genome browser  
 win3\_4242 47925102 47925109 seq\_3811 371543 371549 See win3\_4242 on genome browser  
 win3\_4246 47927736 47927743 seq\_3811 374177 374183 See win3\_4246 on genome browser  
 win3\_4250 47934329 47934336 seq\_3811 380770 380776 See win3\_4250 on genome browser  
 win3\_4254 47984783 47984790 seq\_3811 431224 431230 See win3\_4254 on genome browser  
 win3\_4258 48012933 48012940 seq\_3811 459374 459380 See win3\_4258 on genome browser  
 win2\_4262 48021348 48021355 seq\_3811 467789 467795 See win2\_4262 on genome browser  
 win2\_4266 48084485 48084492 seq\_3811 530926 530932 See win2\_4266 on genome browser  
 win2\_4270 48110173 48110180 seq\_3811 556614 556620 See win2\_4270 on genome browser  
 win3\_4274 48238654 48238661 seq\_3681 118379 118385 See win3\_4274 on genome browser  
 win3\_4278 48465756 48465763 seq\_3681 345481 345487 See win3\_4278 on genome browser  
 win3\_4282 48637017 48637024 seq\_3681 516742 516748 See win3\_4282 on genome browser  
 win2\_4286 48647628 48647635 seq\_3681 527353 527359 See win2\_4286 on genome browser  
 win3\_4290 48654850 48654857 seq\_3681 534575 534581 See win3\_4290 on genome browser  
 win2\_4294 48656533 48656540 seq\_3681 536258 536264 See win2\_4294 on genome browser  
 win2\_4298 48662262 48662269 seq\_3681 541987 541993 See win2\_4298 on genome browser  
 win3\_4302 48711594 48711601 seq\_3704 24780 24786 See win3\_4302 on genome browser  
 win2\_4306 48801484 48801491 seq\_3704 114670 114676 See win2\_4306 on genome browser  
 win2\_4310 48951673 48951680 seq\_3704 264859 264865 See win2\_4310 on genome browser  
 win3\_4314 49190879 49190885 seq\_3704 504065 504070 See win3\_4314 on genome browser  
 win2\_4317 49220109 49220116 seq\_3704 533295 533301 See win2\_4317 on genome browser  
 win3\_4321 49391928 49391935 seq\_3712 146927 146933 See win3\_4321 on genome browser  
 win3\_4325 49449678 49449685 seq\_3712 204677 204683 See win3\_4325 on genome browser  
 win2\_4329 49452635 49452642 seq\_3712 207634 207640 See win2\_4329 on genome browser  
 win2\_4333 49465094 49465101 seq\_3712 220093 220099 See win2\_4333 on genome browser  
 win2\_4337 49577978 49577985 seq\_3712 332977 332983 See win2\_4337 on genome browser  
 win3\_4341 49629846 49629853 seq\_3712 384845 384851 See win3\_4341 on genome browser  
 win2\_4345 49656289 49656296 seq\_3712 411288 411294 See win2\_4345 on genome browser  
 win1\_4349 49738411 49738416 seq\_3712 493410 493414 See win1\_4349 on genome browser  
 win2\_4351 49848797 49848804 seq\_3719 51072 51078 See win2\_4351 on genome browser  
 win2\_4355 49898905 49898912 seq\_3719 101180 101186 See win2\_4355 on genome browser  
 win3\_4359 49996085 49996092 seq\_3719 198360 198366 See win3\_4359 on genome browser  
 win3\_4363 50027306 50027313 seq\_3719 229581 229587 See win3\_4363 on genome browser  
 win2\_4367 50041483 50041490 seq\_3719 243758 243764 See win2\_4367 on genome browser  
 win2\_4371 50044130 50044137 seq\_3719 246405 246411 See win2\_4371 on genome browser  
 win3\_4375 50057647 50057654 seq\_3719 259922 259928 See win3\_4375 on genome browser  
 win2\_4379 50063097 50063104 seq\_3719 265372 265378 See win2\_4379 on genome browser  
 win2\_4383 50079808 50079815 seq\_3719 282083 282089 See win2\_4383 on genome browser  
 win2\_4387 50092281 50092288 seq\_3719 294556 294562 See win2\_4387 on genome browser  
 win2\_4391 50146839 50146846 seq\_3719 349114 349120 See win2\_4391 on genome browser  
 win3\_4395 50178907 50178914 seq\_3719 381182 381188 See win3\_4395 on genome browser  
 win2\_4399 50183377 50183384 seq\_3719 385652 385658 See win2\_4399 on genome browser  
 win3\_4403 50223531 50223538 seq\_3719 425806 425812 See win3\_4403 on genome browser  
 win3\_4407 50268829 50268836 seq\_3719 471104 471110 See win3\_4407 on genome browser  
 win2\_4411 50304906 50304911 seq\_3719 507181 507185 See win2\_4411 on genome browser  
 win2\_4413 50306581 50306588 seq\_3719 508856 508862 See win2\_4413 on genome browser  
 win3\_4417 50306757 50306764 seq\_3719 509032 509038 See win3\_4417 on genome browser  
 win2\_4421 50308885 50308892 seq\_3719 511160 511166 See win2\_4421 on genome browser  
 win3\_4425 50310959 50310966 seq\_3719 513234 513240 See win3\_4425 on genome browser  
 win1\_4429 50311029 50311035 seq\_3719 513304 513309 See win1\_4429 on genome browser  
 win2\_4432 50312908 50312915 seq\_3719 515183 515189 See win2\_4432 on genome browser  
 win3\_4436 50375335 50375342 seq\_3710 36115 36121 See win3\_4436 on genome browser  
 win2\_4440 50434454 50434461 seq\_3710 95234 95240 See win2\_4440 on genome browser  
 win3\_4444 50469815 50469822 seq\_3710 130595 130601 See win3\_4444 on genome browser  
 win2\_4448 50471405 50471412 seq\_3710 132185 132191 See win2\_4448 on genome browser  
 win3\_4452 50475382 50475389 seq\_3710 136162 136168 See win3\_4452 on genome browser  
 win3\_4456 50477420 50477427 seq\_3710 138200 138206 See win3\_4456 on genome browser  
 win1\_4460 50478886 50478892 seq\_3710 139666 139671 See win1\_4460 on genome browser  
 win3\_4463 50483105 50483112 seq\_3710 143885 143891 See win3\_4463 on genome browser  
 win3\_4467 50509547 50509554 seq\_3710 170327 170333 See win3\_4467 on genome browser  
 win2\_4471 50577720 50577727 seq\_3710 238500 238506 See win2\_4471 on genome browser  
 win2\_4475 50671528 50671535 seq\_3710 332308 332314 See win2\_4475 on genome browser  
 win3\_4479 50676405 50676412 seq\_3710 337185 337191 See win3\_4479 on genome browser  
 win2\_4483 50869813 50869820 seq\_3710 530593 530599 See win2\_4483 on genome browser  
 win3\_4487 50880388 50880395 seq\_3686 28 34 See win3\_4487 on genome browser  
 win2\_4491 50892459 50892466 seq\_3686 12099 12105 See win2\_4491 on genome browser  
 win3\_4495 51058224 51058231 seq\_3686 177864 177870 See win3\_4495 on genome browser  
 win3\_4499 51103025 51103032 seq\_3686 222665 222671 See win3\_4499 on genome browser  
 win3\_4503 51268266 51268273 seq\_3686 387906 387912 See win3\_4503 on genome browser  
 win3\_4507 51379435 51379442 seq\_3686 499075 499081 See win3\_4507 on genome browser  
 win3\_4511 51458768 51458775 seq\_3709 39937 39943 See win3\_4511 on genome browser  
 win3\_4515 51724221 51724228 seq\_3709 305390 305396 See win3\_4515 on genome browser  
 win3\_4519 52197621 52197628 seq\_3680 241755 241761 See win3\_4519 on genome browser  
 win2\_4523 52250740 52250747 seq\_3680 294874 294880 See win2\_4523 on genome browser  
 win3\_4527 52420868 52420875 seq\_3680 465002 465008 See win3\_4527 on genome browser  
 win3\_4531 52421328 52421335 seq\_3680 465462 465468 See win3\_4531 on genome browser  
 win3\_4535 52523105 52523112 seq\_3711 36296 36302 See win3\_4535 on genome browser  
 win3\_4539 52798298 52798305 seq\_3711 311489 311495 See win3\_4539 on genome browser  
 win3\_4543 53015949 53015956 seq\_60 557 563 See win3\_4543 on genome browser  
 win2\_4547 53016163 53016173 seq\_60 771 780 See win2\_4547 on genome browser  
 win2\_4554 53016278 53016285 seq\_60 886 892 See win2\_4554 on genome browser  
 win2\_4558 53042079 53042086 seq\_60 26687 26693 See win2\_4558 on genome browser  
 win3\_4562 53098475 53098482 seq\_60 83083 83089 See win3\_4562 on genome browser  
 win3\_4566 53162440 53162447 seq\_60 147048 147054 See win3\_4566 on genome browser  
 win3\_4570 53167490 53167497 seq\_60 152098 152104 See win3\_4570 on genome browser  
 win3\_4574 53168739 53168746 seq\_60 153347 153353 See win3\_4574 on genome browser  
 win2\_4578 53176856 53176863 seq\_60 161464 161470 See win2\_4578 on genome browser  
 win2\_4582 53178274 53178281 seq\_60 162882 162888 See win2\_4582 on genome browser  
 win3\_4586 53183278 53183285 seq\_60 167886 167892 See win3\_4586 on genome browser  
 win3\_4590 53185033 53185040 seq\_60 169641 169647 See win3\_4590 on genome browser  
 win2\_4594 53191918 53191925 seq\_60 176526 176532 See win2\_4594 on genome browser  
 win2\_4598 53235322 53235329 seq\_60 219930 219936 See win2\_4598 on genome browser  
 win3\_4602 53251230 53251237 seq\_60 235838 235844 See win3\_4602 on genome browser  
 win2\_4606 53286325 53286332 seq\_60 270933 270939 See win2\_4606 on genome browser  
 win3\_4610 53286589 53286596 seq\_60 271197 271203 See win3\_4610 on genome browser  
 win3\_4614 53301337 53301344 seq\_60 285945 285951 See win3\_4614 on genome browser  
 win3\_4618 53313147 53313154 seq\_60 297755 297761 See win3\_4618 on genome browser  
 win1\_4622 53336803 53336809 seq\_60 321411 321416 See win1\_4622 on genome browser  
 win3\_4625 53338503 53338510 seq\_60 323111 323117 See win3\_4625 on genome browser  
 win3\_4629 53348446 53348453 seq\_60 333054 333060 See win3\_4629 on genome browser  
 win2\_4633 53385919 53385926 seq\_60 370527 370533 See win2\_4633 on genome browser  
 win2\_4637 53400657 53400664 seq\_60 385265 385271 See win2\_4637 on genome browser  
 win1\_4641 53406804 53406811 seq\_60 391412 391418 See win1\_4641 on genome browser  
 win2\_4645 53408259 53408266 seq\_60 392867 392873 See win2\_4645 on genome browser  
 win3\_4649 53461447 53461454 seq\_60 446055 446061 See win3\_4649 on genome browser  
 win2\_4653 53481287 53481294 seq\_60 465895 465901 See win2\_4653 on genome browser  
 win3\_4657 53629580 53629587 seq\_3715 88120 88126 See win3\_4657 on genome browser  
 win2\_4661 53629830 53629837 seq\_3715 88370 88376 See win2\_4661 on genome browser  
 win2\_4665 53695855 53695862 seq\_3715 154395 154401 See win2\_4665 on genome browser  
 win3\_4669 53735203 53735210 seq\_3715 193743 193749 See win3\_4669 on genome browser  
 win2\_4673 53735260 53735266 seq\_3715 193800 193805 See win2\_4673 on genome browser  
 win3\_4676 53915125 53915132 seq\_3715 373665 373671 See win3\_4676 on genome browser  
 win3\_4680 54119620 54119627 seq\_3723 52275 52281 See win3\_4680 on genome browser  
 win3\_4684 54144568 54144575 seq\_3723 77223 77229 See win3\_4684 on genome browser  
 win3\_4688 54151491 54151498 seq\_3723 84146 84152 See win3\_4688 on genome browser  
 win2\_4692 54165961 54165968 seq\_3723 98616 98622 See win2\_4692 on genome browser  
 win3\_4696 54239530 54239537 seq\_3723 172185 172191 See win3\_4696 on genome browser  
 win2\_4700 54239678 54239687 seq\_3723 172333 172341 See win2\_4700 on genome browser  
 win1\_4706 54250923 54250930 seq\_3723 183578 183584 See win1\_4706 on genome browser  
 win3\_4710 54322859 54322866 seq\_3723 255514 255520 See win3\_4710 on genome browser  
 win3\_4714 54334122 54334129 seq\_3723 266777 266783 See win3\_4714 on genome browser  
 win2\_4718 54336151 54336158 seq\_3723 268806 268812 See win2\_4718 on genome browser  
 win2\_4722 54336227 54336234 seq\_3723 268882 268888 See win2\_4722 on genome browser  
 win3\_4726 54338387 54338394 seq\_3723 271042 271048 See win3\_4726 on genome browser  
 win2\_4730 54362359 54362366 seq\_3723 295014 295020 See win2\_4730 on genome browser  
 win3\_4734 54442348 54442355 seq\_3723 375003 375009 See win3\_4734 on genome browser  
 win2\_4738 54494107 54494114 seq\_3723 426762 426768 See win2\_4738 on genome browser  
 win2\_4742 54602357 54602364 seq\_3716 9888 9894 See win2\_4742 on genome browser  
 win3\_4746 54791500 54791507 seq\_3716 199031 199037 See win3\_4746 on genome browser  
 win1\_4750 54827588 54827593 seq\_3716 235119 235123 See win1\_4750 on genome browser  
 win2\_4752 55001444 55001451 seq\_3716 408975 408981 See win2\_4752 on genome browser  
 win3\_4756 55006178 55006185 seq\_3716 413709 413715 See win3\_4756 on genome browser  
 win2\_4760 55106388 55106395 seq\_3716 513919 513925 See win2\_4760 on genome browser  
 win2\_4764 55368193 55368200 seq\_3717 251364 251370 See win2\_4764 on genome browser  
 win2\_4768 55406572 55406579 seq\_3717 289743 289749 See win2\_4768 on genome browser  
 win2\_4772 55483497 55483504 seq\_3717 366668 366674 See win2\_4772 on genome browser  
 win3\_4776 55542019 55542026 seq\_3717 425190 425196 See win3\_4776 on genome browser  
 win3\_4780 55554278 55554285 seq\_3717 437449 437455 See win3\_4780 on genome browser  
 win2\_4784 55635613 55635620 seq\_3722 5005 5011 See win2\_4784 on genome browser  
 win2\_4788 55772713 55772719 seq\_3722 142105 142110 See win2\_4788 on genome browser  
 win3\_4791 55787500 55787507 seq\_3722 156892 156898 See win3\_4791 on genome browser  
 win2\_4795 55842155 55842162 seq\_3722 211547 211553 See win2\_4795 on genome browser  
 win3\_4799 55897460 55897467 seq\_3722 266852 266858 See win3\_4799 on genome browser  
 win2\_4803 55909037 55909044 seq\_3722 278429 278435 See win2\_4803 on genome browser  
 win2\_4807 56047728 56047735 seq\_3722 417120 417126 See win2\_4807 on genome browser  
 win3\_4811 56068526 56068533 seq\_3722 437918 437924 See win3\_4811 on genome browser  
 win3\_4815 56080658 56080665 seq\_3722 450050 450056 See win3\_4815 on genome browser  
 win3\_4819 56112328 56112335 seq\_3722 481720 481726 See win3\_4819 on genome browser  
 win2\_4823 56112446 56112453 seq\_3722 481838 481844 See win2\_4823 on genome browser  
 win2\_4827 56114548 56114555 seq\_3722 483940 483946 See win2\_4827 on genome browser  
 win3\_4831 56135665 56135672 seq\_3684 64 70 See win3\_4831 on genome browser  
 win3\_4835 56172066 56172073 seq\_3684 36465 36471 See win3\_4835 on genome browser  
 win3\_4839 56183868 56183875 seq\_3684 48267 48273 See win3\_4839 on genome browser  
 win2\_4843 56361117 56361124 seq\_3684 225516 225522 See win2\_4843 on genome browser  
 win3\_4847 56388579 56388586 seq\_3684 252978 252984 See win3\_4847 on genome browser  
 win2\_4851 56600087 56600094 seq\_3684 464486 464492 See win2\_4851 on genome browser  
 win2\_4855 56644205 56644212 seq\_3830 11357 11363 See win2\_4855 on genome browser  
 win2\_4859 56731065 56731072 seq\_3830 98217 98223 See win2\_4859 on genome browser  
 win3\_4863 56970858 56970865 seq\_3830 338010 338016 See win3\_4863 on genome browser  
 win2\_4867 57010159 57010166 seq\_3830 377311 377317 See win2\_4867 on genome browser  
 win2\_4871 57060629 57060636 seq\_3830 427781 427787 See win2\_4871 on genome browser  
 win2\_4875 57117882 57117889 seq\_3830 485034 485040 See win2\_4875 on genome browser  
 win3\_4879 57272001 57272008 seq\_3718 143536 143542 See win3\_4879 on genome browser  
 win3\_4883 57296305 57296312 seq\_3718 167840 167846 See win3\_4883 on genome browser  
 win2\_4887 57335988 57335995 seq\_3718 207523 207529 See win2\_4887 on genome browser  
 win3\_4891 57611209 57611216 seq\_3835 268 274 See win3\_4891 on genome browser  
 win3\_4895 57642436 57642443 seq\_3835 31495 31501 See win3\_4895 on genome browser  
 win2\_4899 57698170 57698177 seq\_3835 87229 87235 See win2\_4899 on genome browser  
 win2\_4903 57703314 57703321 seq\_3835 92373 92379 See win2\_4903 on genome browser  
 win1\_4907 57767378 57767385 seq\_3835 156437 156443 See win1\_4907 on genome browser  
 win3\_4911 57804954 57804961 seq\_3835 194013 194019 See win3\_4911 on genome browser  
 win3\_4915 57810045 57810052 seq\_3835 199104 199110 See win3\_4915 on genome browser  
 win3\_4919 57811825 57811832 seq\_3835 200884 200890 See win3\_4919 on genome browser  
 win2\_4923 57815029 57815036 seq\_3835 204088 204094 See win2\_4923 on genome browser  
 win3\_4927 57822968 57822975 seq\_3835 212027 212033 See win3\_4927 on genome browser  
 win2\_4931 57823197 57823203 seq\_3835 212256 212261 See win2\_4931 on genome browser  
 win2\_4934 57837336 57837343 seq\_3835 226395 226401 See win2\_4934 on genome browser  
 win2\_4938 57837464 57837471 seq\_3835 226523 226529 See win2\_4938 on genome browser  
 win3\_4942 57846884 57846891 seq\_3835 235943 235949 See win3\_4942 on genome browser  
 win3\_4946 57852897 57852904 seq\_3835 241956 241962 See win3\_4946 on genome browser  
 win1\_4950 57866832 57866836 seq\_3835 255891 255894 See win1\_4950 on genome browser  
 win3\_4951 57868189 57868196 seq\_3835 257248 257254 See win3\_4951 on genome browser  
 win2\_4955 57891869 57891876 seq\_3835 280928 280934 See win2\_4955 on genome browser  
 win2\_4959 57893709 57893716 seq\_3835 282768 282774 See win2\_4959 on genome browser  
 win3\_4963 57932364 57932368 seq\_3835 321423 321426 See win3\_4963 on genome browser  
 win3\_4964 57980549 57980556 seq\_3835 369608 369614 See win3\_4964 on genome browser  
 win2\_4968 57994940 57994947 seq\_3835 383999 384005 See win2\_4968 on genome browser  
 win3\_4972 58014211 58014218 seq\_3835 403270 403276 See win3\_4972 on genome browser  
 win2\_4976 58014356 58014363 seq\_3835 403415 403421 See win2\_4976 on genome browser  
 win2\_4980 58026799 58026806 seq\_3835 415858 415864 See win2\_4980 on genome browser  
 win2\_4984 58026926 58026933 seq\_3835 415985 415991 See win2\_4984 on genome browser  
 win2\_4988 58027038 58027045 seq\_3835 416097 416103 See win2\_4988 on genome browser  
 win2\_4992 58034421 58034428 seq\_3835 423480 423486 See win2\_4992 on genome browser  
 win2\_4996 58039442 58039449 seq\_3835 428501 428507 See win2\_4996 on genome browser  
 win2\_5000 58047925 58047932 seq\_3835 436984 436990 See win2\_5000 on genome browser  
 win2\_5004 58052750 58052757 seq\_3835 441809 441815 See win2\_5004 on genome browser  
 win3\_5008 58057170 58057177 seq\_3835 446229 446235 See win3\_5008 on genome browser  
 win1\_5012 58057318 58057325 seq\_3835 446377 446383 See win1\_5012 on genome browser  
 win3\_5016 58058079 58058086 seq\_3835 447138 447144 See win3\_5016 on genome browser  
 win3\_5020 58058603 58058610 seq\_3835 447662 447668 See win3\_5020 on genome browser  
 win3\_5024 58070190 58070197 seq\_3835 459249 459255 See win3\_5024 on genome browser  
 win2\_5028 58073066 58073073 seq\_3835 462125 462131 See win2\_5028 on genome browser  
 win3\_5032 58095873 58095880 seq\_3728 3849 3855 See win3\_5032 on genome browser  
 win2\_5036 58124068 58124075 seq\_3728 32044 32050 See win2\_5036 on genome browser  
 win2\_5040 58149987 58149994 seq\_3728 57963 57969 See win2\_5040 on genome browser  
 win2\_5044 58155205 58155212 seq\_3728 63181 63187 See win2\_5044 on genome browser  
 win2\_5048 58188564 58188571 seq\_3728 96540 96546 See win2\_5048 on genome browser  
 win3\_5052 58192243 58192250 seq\_3728 100219 100225 See win3\_5052 on genome browser  
 win3\_5056 58319504 58319511 seq\_3728 227480 227486 See win3\_5056 on genome browser  
 win3\_5060 58365614 58365621 seq\_3728 273590 273596 See win3\_5060 on genome browser  
 win3\_5064 58370152 58370159 seq\_3728 278128 278134 See win3\_5064 on genome browser  
 win2\_5068 58372485 58372492 seq\_3728 280461 280467 See win2\_5068 on genome browser  
 win3\_5072 58425926 58425933 seq\_3728 333902 333908 See win3\_5072 on genome browser  
 win3\_5076 58503578 58503585 seq\_3728 411554 411560 See win3\_5076 on genome browser  
 win1\_5080 58521097 58521104 seq\_3728 429073 429079 See win1\_5080 on genome browser  
 win1\_5084 58530506 58530513 seq\_3728 438482 438488 See win1\_5084 on genome browser  
 win2\_5088 58537121 58537128 seq\_3728 445097 445103 See win2\_5088 on genome browser  
 win3\_5092 58546185 58546192 seq\_3728 454161 454167 See win3\_5092 on genome browser  
 win3\_5096 58568566 58568573 seq\_3729 360 366 See win3\_5096 on genome browser  
 win3\_5100 58623689 58623696 seq\_3729 55483 55489 See win3\_5100 on genome browser  
 win3\_5104 58693223 58693230 seq\_3729 125017 125023 See win3\_5104 on genome browser  
 win3\_5108 58698642 58698649 seq\_3729 130436 130442 See win3\_5108 on genome browser  
 win1\_5112 58701518 58701523 seq\_3729 133312 133316 See win1\_5112 on genome browser  
 win3\_5114 58706759 58706766 seq\_3729 138553 138559 See win3\_5114 on genome browser  
 win2\_5118 58750409 58750416 seq\_3729 182203 182209 See win2\_5118 on genome browser  
 win2\_5122 58751868 58751875 seq\_3729 183662 183668 See win2\_5122 on genome browser  
 win2\_5126 58770092 58770099 seq\_3729 201886 201892 See win2\_5126 on genome browser  
 win3\_5130 58777542 58777549 seq\_3729 209336 209342 See win3\_5130 on genome browser  
 win3\_5134 58777602 58777609 seq\_3729 209396 209402 See win3\_5134 on genome browser  
 win3\_5138 58813734 58813741 seq\_3729 245528 245534 See win3\_5138 on genome browser  
 win2\_5142 58869595 58869602 seq\_3729 301389 301395 See win2\_5142 on genome browser  
 win3\_5146 58879918 58879925 seq\_3729 311712 311718 See win3\_5146 on genome browser  
 win3\_5150 58901613 58901620 seq\_3729 333407 333413 See win3\_5150 on genome browser  
 win2\_5154 58959267 58959274 seq\_3729 391061 391067 See win2\_5154 on genome browser  
 win2\_5158 58965759 58965766 seq\_3729 397553 397559 See win2\_5158 on genome browser  
 win1\_5162 58976645 58976650 seq\_3729 408439 408443 See win1\_5162 on genome browser  
 win3\_5164 58985582 58985589 seq\_3729 417376 417382 See win3\_5164 on genome browser  
 win3\_5168 58999356 58999363 seq\_3729 431150 431156 See win3\_5168 on genome browser  
 win2\_5172 59022778 59022785 seq\_3729 454572 454578 See win2\_5172 on genome browser  
 win2\_5176 59161328 59161335 seq\_3822 117181 117187 See win2\_5176 on genome browser  
 win3\_5180 59208733 59208740 seq\_3822 164586 164592 See win3\_5180 on genome browser  
 win3\_5184 59258654 59258661 seq\_3822 214507 214513 See win3\_5184 on genome browser  
 win2\_5188 59263906 59263913 seq\_3822 219759 219765 See win2\_5188 on genome browser  
 win3\_5192 59280326 59280333 seq\_3822 236179 236185 See win3\_5192 on genome browser  
 win1\_5196 59308794 59308800 seq\_3822 264647 264652 See win1\_5196 on genome browser  
 win2\_5199 59354744 59354751 seq\_3822 310597 310603 See win2\_5199 on genome browser  
 win2\_5203 59387697 59387704 seq\_3822 343550 343556 See win2\_5203 on genome browser  
 win2\_5207 59406748 59406755 seq\_3822 362601 362607 See win2\_5207 on genome browser  
 win3\_5211 59419760 59419767 seq\_3822 375613 375619 See win3\_5211 on genome browser  
 win3\_5215 59432668 59432675 seq\_3822 388521 388527 See win3\_5215 on genome browser  
 win2\_5219 59434360 59434371 seq\_3822 390213 390223 See win2\_5219 on genome browser  
 win2\_5227 59485447 59485454 seq\_3822 441300 441306 See win2\_5227 on genome browser  
 win3\_5231 59511529 59511536 seq\_3814 2826 2832 See win3\_5231 on genome browser  
 win2\_5235 59555663 59555670 seq\_3814 46960 46966 See win2\_5235 on genome browser  
 win3\_5239 59568778 59568785 seq\_3814 60075 60081 See win3\_5239 on genome browser  
 win2\_5243 59803618 59803625 seq\_3814 294915 294921 See win2\_5243 on genome browser  
 win2\_5247 59917356 59917363 seq\_3814 408653 408659 See win2\_5247 on genome browser  
 win3\_5251 59924161 59924168 seq\_3814 415458 415464 See win3\_5251 on genome browser  
 win2\_5255 59978803 59978810 seq\_72 7049 7055 See win2\_5255 on genome browser  
 win2\_5259 59987896 59987903 seq\_72 16142 16148 See win2\_5259 on genome browser  
 win3\_5263 60029799 60029806 seq\_72 58045 58051 See win3\_5263 on genome browser  
 win2\_5267 60075305 60075312 seq\_72 103551 103557 See win2\_5267 on genome browser  
 win2\_5271 60119696 60119703 seq\_72 147942 147948 See win2\_5271 on genome browser  
 win3\_5275 60150896 60150903 seq\_72 179142 179148 See win3\_5275 on genome browser  
 win2\_5279 60196241 60196248 seq\_72 224487 224493 See win2\_5279 on genome browser  
 win3\_5283 60206014 60206021 seq\_72 234260 234266 See win3\_5283 on genome browser  
 win2\_5287 60212207 60212214 seq\_72 240453 240459 See win2\_5287 on genome browser  
 win1\_5291 60222245 60222252 seq\_72 250491 250497 See win1\_5291 on genome browser  
 win1\_5295 60325564 60325571 seq\_72 353810 353816 See win1\_5295 on genome browser  
 win2\_5299 60334924 60334931 seq\_72 363170 363176 See win2\_5299 on genome browser  
 win1\_5303 60348517 60348524 seq\_72 376763 376769 See win1\_5303 on genome browser  
 win1\_5307 60351748 60351755 seq\_72 379994 380000 See win1\_5307 on genome browser  
 win1\_5311 60353376 60353383 seq\_72 381622 381628 See win1\_5311 on genome browser  
 win3\_5315 60368068 60368075 seq\_72 396314 396320 See win3\_5315 on genome browser  
 win2\_5319 60392529 60392536 seq\_72 420775 420781 See win2\_5319 on genome browser  
 win3\_5323 60444095 60444102 seq\_3735 22987 22993 See win3\_5323 on genome browser  
 win3\_5327 60445798 60445805 seq\_3735 24690 24696 See win3\_5327 on genome browser  
 win2\_5331 60450881 60450888 seq\_3735 29773 29779 See win2\_5331 on genome browser  
 win3\_5335 60466748 60466755 seq\_3735 45640 45646 See win3\_5335 on genome browser  
 win3\_5339 60624219 60624226 seq\_3735 203111 203117 See win3\_5339 on genome browser  
 win2\_5343 60651010 60651017 seq\_3735 229902 229908 See win2\_5343 on genome browser  
 win3\_5347 60664920 60664927 seq\_3735 243812 243818 See win3\_5347 on genome browser  
 win3\_5351 60733713 60733720 seq\_3735 312605 312611 See win3\_5351 on genome browser  
 win3\_5355 60739760 60739767 seq\_3735 318652 318658 See win3\_5355 on genome browser  
 win3\_5359 60776967 60776974 seq\_3735 355859 355865 See win3\_5359 on genome browser  
 win3\_5363 60802237 60802244 seq\_3735 381129 381135 See win3\_5363 on genome browser  
 win1\_5367 60810819 60810826 seq\_3735 389711 389717 See win1\_5367 on genome browser  
 win3\_5371 60834034 60834041 seq\_3735 412926 412932 See win3\_5371 on genome browser  
 win2\_5375 60845553 60845560 seq\_3735 424445 424451 See win2\_5375 on genome browser  
 win2\_5379 60904583 60904590 seq\_3721 41684 41690 See win2\_5379 on genome browser  
 win2\_5383 60914366 60914373 seq\_3721 51467 51473 See win2\_5383 on genome browser  
 win3\_5387 60937703 60937710 seq\_3721 74804 74810 See win3\_5387 on genome browser  
 win3\_5391 61149101 61149108 seq\_3721 286202 286208 See win3\_5391 on genome browser  
 win1\_5395 61181921 61181928 seq\_3721 319022 319028 See win1\_5395 on genome browser  
 win2\_5399 61331123 61331130 seq\_3720 29981 29987 See win2\_5399 on genome browser  
 win3\_5403 61353238 61353245 seq\_3720 52096 52102 See win3\_5403 on genome browser  
 win3\_5407 61358572 61358579 seq\_3720 57430 57436 See win3\_5407 on genome browser  
 win3\_5411 61486709 61486716 seq\_3720 185567 185573 See win3\_5411 on genome browser  
 win1\_5415 61515892 61515897 seq\_3720 214750 214754 See win1\_5415 on genome browser  
 win3\_5417 61617537 61617544 seq\_3720 316395 316401 See win3\_5417 on genome browser  
 win2\_5421 61658541 61658548 seq\_3720 357399 357405 See win2\_5421 on genome browser  
 win3\_5425 61677064 61677071 seq\_3720 375922 375928 See win3\_5425 on genome browser  
 win3\_5429 61739276 61739283 seq\_3733 316 322 See win3\_5429 on genome browser  
 win3\_5433 61825260 61825267 seq\_3733 86300 86306 See win3\_5433 on genome browser  
 win2\_5437 61834797 61834804 seq\_3733 95837 95843 See win2\_5437 on genome browser  
 win3\_5441 61879192 61879199 seq\_3733 140232 140238 See win3\_5441 on genome browser  
 win2\_5445 61901148 61901155 seq\_3733 162188 162194 See win2\_5445 on genome browser  
 win3\_5449 61976720 61976727 seq\_3733 237760 237766 See win3\_5449 on genome browser  
 win1\_5453 61984749 61984755 seq\_3733 245789 245794 See win1\_5453 on genome browser  
 win2\_5456 62020626 62020633 seq\_3733 281666 281672 See win2\_5456 on genome browser  
 win2\_5460 62042227 62042234 seq\_3733 303267 303273 See win2\_5460 on genome browser  
 win3\_5464 62056700 62056707 seq\_3733 317740 317746 See win3\_5464 on genome browser  
 win3\_5468 62099931 62099938 seq\_3733 360971 360977 See win3\_5468 on genome browser  
 win2\_5472 62100008 62100015 seq\_3733 361048 361054 See win2\_5472 on genome browser  
 win1\_5476 62118210 62118216 seq\_3733 379250 379255 See win1\_5476 on genome browser  
 win2\_5479 62156578 62156585 seq\_3726 363 369 See win2\_5479 on genome browser  
 win3\_5483 62170110 62170117 seq\_3726 13895 13901 See win3\_5483 on genome browser  
 win3\_5487 62177689 62177696 seq\_3726 21474 21480 See win3\_5487 on genome browser  
 win2\_5491 62180234 62180241 seq\_3726 24019 24025 See win2\_5491 on genome browser  
 win1\_5495 62188876 62188880 seq\_3726 32661 32664 See win1\_5495 on genome browser  
 win1\_5496 62191031 62191036 seq\_3726 34816 34820 See win1\_5496 on genome browser  
 win2\_5498 62192185 62192192 seq\_3726 35970 35976 See win2\_5498 on genome browser  
 win2\_5502 62204471 62204478 seq\_3726 48256 48262 See win2\_5502 on genome browser  
 win2\_5506 62204537 62204544 seq\_3726 48322 48328 See win2\_5506 on genome browser  
 win2\_5510 62233351 62233358 seq\_3726 77136 77142 See win2\_5510 on genome browser  
 win1\_5514 62234535 62234539 seq\_3726 78320 78323 See win1\_5514 on genome browser  
 win3\_5515 62237762 62237769 seq\_3726 81547 81553 See win3\_5515 on genome browser  
 win2\_5519 62266838 62266845 seq\_3726 110623 110629 See win2\_5519 on genome browser  
 win3\_5523 62267035 62267042 seq\_3726 110820 110826 See win3\_5523 on genome browser  
 win3\_5527 62270009 62270016 seq\_3726 113794 113800 See win3\_5527 on genome browser  
 win3\_5531 62313510 62313517 seq\_3726 157295 157301 See win3\_5531 on genome browser  
 win1\_5535 62317138 62317142 seq\_3726 160923 160926 See win1\_5535 on genome browser  
 win3\_5536 62384147 62384154 seq\_3726 227932 227938 See win3\_5536 on genome browser  
 win2\_5540 62451840 62451847 seq\_3726 295625 295631 See win2\_5540 on genome browser  
 win2\_5544 62476662 62476669 seq\_3726 320447 320453 See win2\_5544 on genome browser  
 win3\_5548 62481373 62481380 seq\_3726 325158 325164 See win3\_5548 on genome browser  
 win1\_5552 62540166 62540171 seq\_3726 383951 383955 See win1\_5552 on genome browser  
 win2\_5554 62542606 62542613 seq\_3726 386391 386397 See win2\_5554 on genome browser  
 win2\_5558 62555122 62555129 seq\_3726 398907 398913 See win2\_5558 on genome browser  
 win1\_5562 62588000 62588007 seq\_3675 17448 17454 See win1\_5562 on genome browser  
 win2\_5566 62674894 62674901 seq\_3675 104342 104348 See win2\_5566 on genome browser  
 win3\_5570 63108721 63108728 seq\_3685 126885 126891 See win3\_5570 on genome browser  
 win2\_5574 63115323 63115330 seq\_3685 133487 133493 See win2\_5574 on genome browser  
 win2\_5578 63142502 63142509 seq\_3685 160666 160672 See win2\_5578 on genome browser  
 win3\_5582 63169849 63169856 seq\_3685 188013 188019 See win3\_5582 on genome browser  
 win2\_5586 63294420 63294427 seq\_3685 312584 312590 See win2\_5586 on genome browser  
 win2\_5590 63399642 63399649 seq\_84 10762 10768 See win2\_5590 on genome browser  
 win3\_5594 63433434 63433441 seq\_84 44554 44560 See win3\_5594 on genome browser  
 win1\_5598 63435861 63435868 seq\_84 46981 46987 See win1\_5598 on genome browser  
 win2\_5602 63439560 63439567 seq\_84 50680 50686 See win2\_5602 on genome browser  
 win3\_5606 63445373 63445380 seq\_84 56493 56499 See win3\_5606 on genome browser  
 win3\_5610 63461087 63461094 seq\_84 72207 72213 See win3\_5610 on genome browser  
 win2\_5614 63468019 63468026 seq\_84 79139 79145 See win2\_5614 on genome browser  
 win2\_5618 63469267 63469274 seq\_84 80387 80393 See win2\_5618 on genome browser  
 win3\_5622 63471082 63471089 seq\_84 82202 82208 See win3\_5622 on genome browser  
 win3\_5626 63479236 63479243 seq\_84 90356 90362 See win3\_5626 on genome browser  
 win3\_5630 63481050 63481057 seq\_84 92170 92176 See win3\_5630 on genome browser  
 win2\_5634 63482023 63482030 seq\_84 93143 93149 See win2\_5634 on genome browser  
 win3\_5638 63484512 63484519 seq\_84 95632 95638 See win3\_5638 on genome browser  
 win1\_5642 63489407 63489411 seq\_84 100527 100530 See win1\_5642 on genome browser  
 win2\_5643 63490642 63490649 seq\_84 101762 101768 See win2\_5643 on genome browser  
 win2\_5647 63495120 63495127 seq\_84 106240 106246 See win2\_5647 on genome browser  
 win2\_5651 63498065 63498072 seq\_84 109185 109191 See win2\_5651 on genome browser  
 win3\_5655 63516184 63516191 seq\_84 127304 127310 See win3\_5655 on genome browser  
 win2\_5659 63545617 63545624 seq\_84 156737 156743 See win2\_5659 on genome browser  
 win2\_5663 63571356 63571363 seq\_84 182476 182482 See win2\_5663 on genome browser  
 win1\_5667 63611432 63611439 seq\_84 222552 222558 See win1\_5667 on genome browser  
 win3\_5671 63642680 63642687 seq\_84 253800 253806 See win3\_5671 on genome browser  
 win2\_5675 63644713 63644720 seq\_84 255833 255839 See win2\_5675 on genome browser  
 win1\_5679 63661240 63661245 seq\_84 272360 272364 See win1\_5679 on genome browser  
 win3\_5681 63662917 63662924 seq\_84 274037 274043 See win3\_5681 on genome browser  
 win2\_5685 63693457 63693464 seq\_84 304577 304583 See win2\_5685 on genome browser  
 win2\_5689 63727041 63727048 seq\_84 338161 338167 See win2\_5689 on genome browser  
 win1\_5693 63731201 63731208 seq\_84 342321 342327 See win1\_5693 on genome browser  
 win1\_5697 63772452 63772457 seq\_84 383572 383576 See win1\_5697 on genome browser  
 win3\_5699 63870368 63870375 seq\_3725 78701 78707 See win3\_5699 on genome browser  
 win2\_5703 63870455 63870462 seq\_3725 78788 78794 See win2\_5703 on genome browser  
 win2\_5707 63902095 63902102 seq\_3725 110428 110434 See win2\_5707 on genome browser  
 win2\_5711 63918326 63918333 seq\_3725 126659 126665 See win2\_5711 on genome browser  
 win1\_5715 63930174 63930181 seq\_3725 138507 138513 See win1\_5715 on genome browser  
 win3\_5719 63969922 63969929 seq\_3725 178255 178261 See win3\_5719 on genome browser  
 win2\_5723 63979364 63979371 seq\_3725 187697 187703 See win2\_5723 on genome browser  
 win3\_5727 64110743 64110750 seq\_3725 319076 319082 See win3\_5727 on genome browser  
 win2\_5731 64122696 64122703 seq\_3725 331029 331035 See win2\_5731 on genome browser  
 win2\_5735 64197508 64197515 seq\_86 9082 9088 See win2\_5735 on genome browser  
 win2\_5739 64239916 64239923 seq\_86 51490 51496 See win2\_5739 on genome browser  
 win1\_5743 64246342 64246347 seq\_86 57916 57920 See win1\_5743 on genome browser  
 win3\_5745 64248536 64248543 seq\_86 60110 60116 See win3\_5745 on genome browser  
 win2\_5749 64250568 64250575 seq\_86 62142 62148 See win2\_5749 on genome browser  
 win2\_5753 64250666 64250673 seq\_86 62240 62246 See win2\_5753 on genome browser  
 win2\_5757 64280342 64280349 seq\_86 91916 91922 See win2\_5757 on genome browser  
 win3\_5761 64288202 64288209 seq\_86 99776 99782 See win3\_5761 on genome browser  
 win1\_5765 64300645 64300650 seq\_86 112219 112223 See win1\_5765 on genome browser  
 win2\_5767 64361798 64361805 seq\_86 173372 173378 See win2\_5767 on genome browser  
 win2\_5771 64440653 64440660 seq\_86 252227 252233 See win2\_5771 on genome browser  
 win3\_5775 64468515 64468522 seq\_86 280089 280095 See win3\_5775 on genome browser  
 win3\_5779 64548758 64548765 seq\_86 360332 360338 See win3\_5779 on genome browser  
 win3\_5783 64568722 64568729 seq\_86 380296 380302 See win3\_5783 on genome browser  
 win3\_5787 64618510 64618517 seq\_90 35320 35326 See win3\_5787 on genome browser  
 win2\_5791 64618924 64618931 seq\_90 35734 35740 See win2\_5791 on genome browser  
 win2\_5795 64655788 64655795 seq\_90 72598 72604 See win2\_5795 on genome browser  
 win3\_5799 64693800 64693807 seq\_90 110610 110616 See win3\_5799 on genome browser  
 win3\_5803 64713785 64713792 seq\_90 130595 130601 See win3\_5803 on genome browser  
 win3\_5807 64745532 64745539 seq\_90 162342 162348 See win3\_5807 on genome browser  
 win3\_5811 64772381 64772388 seq\_90 189191 189197 See win3\_5811 on genome browser  
 win3\_5815 64813348 64813355 seq\_90 230158 230164 See win3\_5815 on genome browser  
 win1\_5819 64814572 64814576 seq\_90 231382 231385 See win1\_5819 on genome browser  
 win2\_5820 64816494 64816501 seq\_90 233304 233310 See win2\_5820 on genome browser  
 win1\_5824 64819138 64819143 seq\_90 235948 235952 See win1\_5824 on genome browser  
 win3\_5826 64863308 64863315 seq\_90 280118 280124 See win3\_5826 on genome browser  
 win3\_5830 64867671 64867678 seq\_90 284481 284487 See win3\_5830 on genome browser  
 win2\_5834 64878400 64878407 seq\_90 295210 295216 See win2\_5834 on genome browser  
 win3\_5838 64893932 64893939 seq\_90 310742 310748 See win3\_5838 on genome browser  
 win2\_5842 64897398 64897405 seq\_90 314208 314214 See win2\_5842 on genome browser  
 win2\_5846 64902067 64902074 seq\_90 318877 318883 See win2\_5846 on genome browser  
 win1\_5850 64913649 64913656 seq\_90 330459 330465 See win1\_5850 on genome browser  
 win2\_5854 64921773 64921780 seq\_90 338583 338589 See win2\_5854 on genome browser  
 win2\_5858 64943314 64943319 seq\_90 360124 360128 See win2\_5858 on genome browser  
 win3\_5860 64954861 64954868 seq\_90 371671 371677 See win3\_5860 on genome browser  
 win2\_5864 65062446 65062453 seq\_91 94025 94031 See win2\_5864 on genome browser  
 win3\_5868 65086726 65086733 seq\_91 118305 118311 See win3\_5868 on genome browser  
 win3\_5872 65113906 65113913 seq\_91 145485 145491 See win3\_5872 on genome browser  
 win3\_5876 65140351 65140358 seq\_91 171930 171936 See win3\_5876 on genome browser  
 win3\_5880 65158479 65158486 seq\_91 190058 190064 See win3\_5880 on genome browser  
 win2\_5884 65209162 65209169 seq\_91 240741 240747 See win2\_5884 on genome browser  
 win2\_5888 65240665 65240672 seq\_91 272244 272250 See win2\_5888 on genome browser  
 win1\_5892 65241853 65241859 seq\_91 273432 273437 See win1\_5892 on genome browser  
 win2\_5895 65242171 65242178 seq\_91 273750 273756 See win2\_5895 on genome browser  
 win3\_5899 65337910 65337917 seq\_91 369489 369495 See win3\_5899 on genome browser  
 win2\_5903 65368838 65368845 seq\_92 18445 18451 See win2\_5903 on genome browser  
 win2\_5907 65391254 65391261 seq\_92 40861 40867 See win2\_5907 on genome browser  
 win2\_5911 65399713 65399720 seq\_92 49320 49326 See win2\_5911 on genome browser  
 win2\_5915 65429027 65429034 seq\_92 78634 78640 See win2\_5915 on genome browser  
 win3\_5919 65485574 65485581 seq\_92 135181 135187 See win3\_5919 on genome browser  
 win2\_5923 65537643 65537650 seq\_92 187250 187256 See win2\_5923 on genome browser  
 win3\_5927 65558336 65558343 seq\_92 207943 207949 See win3\_5927 on genome browser  
 win3\_5931 65590928 65590935 seq\_92 240535 240541 See win3\_5931 on genome browser  
 win2\_5935 65592613 65592620 seq\_92 242220 242226 See win2\_5935 on genome browser  
 win2\_5939 65614497 65614504 seq\_92 264104 264110 See win2\_5939 on genome browser  
 win3\_5943 65660542 65660549 seq\_92 310149 310155 See win3\_5943 on genome browser  
 win2\_5947 65693798 65693805 seq\_92 343405 343411 See win2\_5947 on genome browser  
 win2\_5951 65704584 65704591 seq\_92 354191 354197 See win2\_5951 on genome browser  
 win3\_5955 65704620 65704627 seq\_92 354227 354233 See win3\_5955 on genome browser  
 win2\_5959 65707813 65707820 seq\_92 357420 357426 See win2\_5959 on genome browser  
 win3\_5963 65807463 65807470 seq\_3727 77804 77810 See win3\_5963 on genome browser  
 win2\_5967 65854479 65854486 seq\_3727 124820 124826 See win2\_5967 on genome browser  
 win3\_5971 65975682 65975689 seq\_3727 246023 246029 See win3\_5971 on genome browser  
 win3\_5975 66064856 66064863 seq\_3727 335197 335203 See win3\_5975 on genome browser  
 win2\_5979 66067407 66067414 seq\_3727 337748 337754 See win2\_5979 on genome browser  
 win3\_5983 66205483 66205490 seq\_3677 98730 98736 See win3\_5983 on genome browser  
 win2\_5987 66208880 66208887 seq\_3677 102127 102133 See win2\_5987 on genome browser  
 win3\_5991 66241481 66241488 seq\_3677 134728 134734 See win3\_5991 on genome browser  
 win3\_5995 66263508 66263515 seq\_3677 156755 156761 See win3\_5995 on genome browser  
 win2\_5999 66345479 66345486 seq\_3677 238726 238732 See win2\_5999 on genome browser  
 win2\_6003 66347786 66347793 seq\_3677 241033 241039 See win2\_6003 on genome browser  
 win3\_6007 66395271 66395278 seq\_3677 288518 288524 See win3\_6007 on genome browser  
 win2\_6011 66455435 66455442 seq\_3677 348682 348688 See win2\_6011 on genome browser  
 win2\_6015 66457732 66457739 seq\_3677 350979 350985 See win2\_6015 on genome browser  
 win2\_6019 66465833 66465840 seq\_3677 359080 359086 See win2\_6019 on genome browser  
 win2\_6023 66555814 66555821 seq\_3724 79955 79961 See win2\_6023 on genome browser  
 win2\_6027 66641577 66641584 seq\_3724 165718 165724 See win2\_6027 on genome browser  
 win2\_6031 66662095 66662102 seq\_3724 186236 186242 See win2\_6031 on genome browser  
 win2\_6035 66876536 66876543 seq\_96 32792 32798 See win2\_6035 on genome browser  
 win1\_6039 66902881 66902886 seq\_96 59137 59141 See win1\_6039 on genome browser  
 win3\_6041 66927667 66927674 seq\_96 83923 83929 See win3\_6041 on genome browser  
 win3\_6045 67024527 67024534 seq\_96 180783 180789 See win3\_6045 on genome browser  
 win3\_6049 67147720 67147727 seq\_96 303976 303982 See win3\_6049 on genome browser  
 win3\_6053 67155643 67155650 seq\_96 311899 311905 See win3\_6053 on genome browser  
 win1\_6057 67198194 67198201 seq\_96 354450 354456 See win1\_6057 on genome browser  
 win2\_6061 67199464 67199471 seq\_96 355720 355726 See win2\_6061 on genome browser  
 win2\_6065 67287180 67287187 seq\_3736 76618 76624 See win2\_6065 on genome browser  
 win2\_6069 67357067 67357074 seq\_3736 146505 146511 See win2\_6069 on genome browser  
 win2\_6073 67397481 67397488 seq\_3736 186919 186925 See win2\_6073 on genome browser  
 win3\_6077 67454054 67454061 seq\_3736 243492 243498 See win3\_6077 on genome browser  
 win3\_6081 67480523 67480530 seq\_3736 269961 269967 See win3\_6081 on genome browser  
 win2\_6085 67485382 67485389 seq\_3736 274820 274826 See win2\_6085 on genome browser  
 win3\_6089 67559662 67559669 seq\_3736 349100 349106 See win3\_6089 on genome browser  
 win2\_6093 67609984 67609991 seq\_3821 39941 39947 See win2\_6093 on genome browser  
 win2\_6097 67623790 67623797 seq\_3821 53747 53753 See win2\_6097 on genome browser  
 win3\_6101 67623805 67623812 seq\_3821 53762 53768 See win3\_6101 on genome browser  
 win2\_6105 67670554 67670561 seq\_3821 100511 100517 See win2\_6105 on genome browser  
 win2\_6109 67708647 67708654 seq\_3821 138604 138610 See win2\_6109 on genome browser  
 win3\_6113 67708655 67708662 seq\_3821 138612 138618 See win3\_6113 on genome browser  
 win3\_6117 67836105 67836112 seq\_3821 266062 266068 See win3\_6117 on genome browser  
 win3\_6121 67880083 67880090 seq\_3821 310040 310046 See win3\_6121 on genome browser  
 win2\_6125 67973304 67973311 seq\_99 44847 44853 See win2\_6125 on genome browser  
 win3\_6129 68009054 68009061 seq\_99 80597 80603 See win3\_6129 on genome browser  
 win2\_6133 68050719 68050726 seq\_99 122262 122268 See win2\_6133 on genome browser  
 win2\_6137 68063881 68063888 seq\_99 135424 135430 See win2\_6137 on genome browser  
 win1\_6141 68178202 68178208 seq\_99 249745 249750 See win1\_6141 on genome browser  
 win1\_6144 68264055 68264062 seq\_99 335598 335604 See win1\_6144 on genome browser  
 win3\_6148 68285416 68285423 seq\_3731 298 304 See win3\_6148 on genome browser  
 win1\_6152 68292681 68292686 seq\_3731 7563 7567 See win1\_6152 on genome browser  
 win3\_6154 68294057 68294064 seq\_3731 8939 8945 See win3\_6154 on genome browser  
 win2\_6158 68294301 68294308 seq\_3731 9183 9189 See win2\_6158 on genome browser  
 win2\_6162 68302231 68302238 seq\_3731 17113 17119 See win2\_6162 on genome browser  
 win3\_6166 68302798 68302804 seq\_3731 17680 17685 See win3\_6166 on genome browser  
 win2\_6169 68302932 68302939 seq\_3731 17814 17820 See win2\_6169 on genome browser  
 win3\_6173 68389870 68389877 seq\_3731 104752 104758 See win3\_6173 on genome browser  
 win2\_6177 68441843 68441850 seq\_3731 156725 156731 See win2\_6177 on genome browser  
 win3\_6181 68643582 68643589 seq\_101 2960 2966 See win3\_6181 on genome browser  
 win3\_6185 68643766 68643773 seq\_101 3144 3150 See win3\_6185 on genome browser  
 win2\_6189 68672344 68672351 seq\_101 31722 31728 See win2\_6189 on genome browser  
 win3\_6193 68675206 68675213 seq\_101 34584 34590 See win3\_6193 on genome browser  
 win1\_6197 68676849 68676856 seq\_101 36227 36233 See win1\_6197 on genome browser  
 win1\_6201 68737019 68737026 seq\_101 96397 96403 See win1\_6201 on genome browser  
 win2\_6205 68838259 68838266 seq\_101 197637 197643 See win2\_6205 on genome browser  
 win3\_6209 68923506 68923513 seq\_101 282884 282890 See win3\_6209 on genome browser  
 win3\_6213 68940600 68940607 seq\_101 299978 299984 See win3\_6213 on genome browser  
 win2\_6217 68950061 68950068 seq\_101 309439 309445 See win2\_6217 on genome browser  
 win3\_6221 69124317 69124324 seq\_3730 136082 136088 See win3\_6221 on genome browser  
 win2\_6225 69226518 69226525 seq\_3730 238283 238289 See win2\_6225 on genome browser  
 win3\_6229 69295485 69295492 seq\_3730 307250 307256 See win3\_6229 on genome browser  
 win2\_6233 69359281 69359286 seq\_3732 27794 27798 See win2\_6233 on genome browser  
 win2\_6235 69359598 69359605 seq\_3732 28111 28117 See win2\_6235 on genome browser  
 win3\_6239 69367018 69367025 seq\_3732 35531 35537 See win3\_6239 on genome browser  
 win2\_6243 69442407 69442414 seq\_3732 110920 110926 See win2\_6243 on genome browser  
 win2\_6247 69474542 69474549 seq\_3732 143055 143061 See win2\_6247 on genome browser  
 win3\_6251 69590613 69590620 seq\_3732 259126 259132 See win3\_6251 on genome browser  
 win2\_6255 69663506 69663513 seq\_3732 332019 332025 See win2\_6255 on genome browser  
 win3\_6259 69724554 69724561 seq\_3734 50773 50779 See win3\_6259 on genome browser  
 win3\_6263 69745077 69745084 seq\_3734 71296 71302 See win3\_6263 on genome browser  
 win3\_6267 69776420 69776427 seq\_3734 102639 102645 See win3\_6267 on genome browser  
 win2\_6271 69776864 69776871 seq\_3734 103083 103089 See win2\_6271 on genome browser  
 win2\_6275 69951056 69951063 seq\_3734 277275 277281 See win2\_6275 on genome browser  
 win2\_6279 69958988 69958995 seq\_3734 285207 285213 See win2\_6279 on genome browser  
 win2\_6283 70135504 70135511 seq\_3737 120509 120515 See win2\_6283 on genome browser  
 win1\_6287 70371449 70371456 seq\_3674 15704 15710 See win1\_6287 on genome browser  
 win2\_6291 70374793 70374800 seq\_3674 19048 19054 See win2\_6291 on genome browser  
 win2\_6295 70452631 70452638 seq\_3674 96886 96892 See win2\_6295 on genome browser  
 win3\_6299 70455466 70455473 seq\_3674 99721 99727 See win3\_6299 on genome browser  
 win3\_6303 70472219 70472226 seq\_3674 116474 116480 See win3\_6303 on genome browser  
 win3\_6307 70476246 70476253 seq\_3674 120501 120507 See win3\_6307 on genome browser  
 win3\_6311 70527067 70527074 seq\_3674 171322 171328 See win3\_6311 on genome browser  
 win1\_6315 70527239 70527246 seq\_3674 171494 171500 See win1\_6315 on genome browser  
 win3\_6319 70528619 70528626 seq\_3674 172874 172880 See win3\_6319 on genome browser  
 win3\_6323 70686903 70686910 seq\_3753 23 29 See win3\_6323 on genome browser  
 win2\_6327 70687190 70687197 seq\_3753 310 316 See win2\_6327 on genome browser  
 win2\_6331 70821345 70821352 seq\_3753 134465 134471 See win2\_6331 on genome browser  
 win3\_6335 71024920 71024927 seq\_3818 13790 13796 See win3\_6335 on genome browser  
 win2\_6339 71097816 71097823 seq\_3818 86686 86692 See win2\_6339 on genome browser  
 win3\_6343 71173778 71173785 seq\_3818 162648 162654 See win3\_6343 on genome browser  
 win3\_6347 71180060 71180067 seq\_3818 168930 168936 See win3\_6347 on genome browser  
 win3\_6351 71184396 71184403 seq\_3818 173266 173272 See win3\_6351 on genome browser  
 win2\_6355 71202081 71202088 seq\_3818 190951 190957 See win2\_6355 on genome browser  
 win2\_6359 71295911 71295918 seq\_3818 284781 284787 See win2\_6359 on genome browser  
 win3\_6363 71342725 71342732 seq\_109 8728 8734 See win3\_6363 on genome browser  
 win2\_6367 71342884 71342891 seq\_109 8887 8893 See win2\_6367 on genome browser  
 win3\_6371 71344941 71344948 seq\_109 10944 10950 See win3\_6371 on genome browser  
 win3\_6375 71366345 71366352 seq\_109 32348 32354 See win3\_6375 on genome browser  
 win2\_6379 71382439 71382446 seq\_109 48442 48448 See win2\_6379 on genome browser  
 win2\_6383 71409680 71409687 seq\_109 75683 75689 See win2\_6383 on genome browser  
 win3\_6387 71412938 71412945 seq\_109 78941 78947 See win3\_6387 on genome browser  
 win2\_6391 71419034 71419041 seq\_109 85037 85043 See win2\_6391 on genome browser  
 win3\_6395 71431408 71431415 seq\_109 97411 97417 See win3\_6395 on genome browser  
 win2\_6399 71444154 71444161 seq\_109 110157 110163 See win2\_6399 on genome browser  
 win3\_6403 71459224 71459231 seq\_109 125227 125233 See win3\_6403 on genome browser  
 win1\_6407 71466012 71466017 seq\_109 132015 132019 See win1\_6407 on genome browser  
 win2\_6409 71471089 71471096 seq\_109 137092 137098 See win2\_6409 on genome browser  
 win3\_6413 71481645 71481652 seq\_109 147648 147654 See win3\_6413 on genome browser  
 win2\_6417 71493325 71493332 seq\_109 159328 159334 See win2\_6417 on genome browser  
 win3\_6421 71508759 71508766 seq\_109 174762 174768 See win3\_6421 on genome browser  
 win1\_6425 71525480 71525486 seq\_109 191483 191488 See win1\_6425 on genome browser  
 win2\_6428 71527799 71527806 seq\_109 193802 193808 See win2\_6428 on genome browser  
 win3\_6432 71542344 71542351 seq\_109 208347 208353 See win3\_6432 on genome browser  
 win2\_6436 71594806 71594813 seq\_109 260809 260815 See win2\_6436 on genome browser  
 win3\_6440 71594906 71594913 seq\_109 260909 260915 See win3\_6440 on genome browser  
 win3\_6444 71626034 71626041 seq\_109 292037 292043 See win3\_6444 on genome browser  
 win3\_6448 71639033 71639040 seq\_109 305036 305042 See win3\_6448 on genome browser  
 win2\_6452 71741945 71741952 seq\_3744 87409 87415 See win2\_6452 on genome browser  
 win3\_6456 71859106 71859113 seq\_3744 204570 204576 See win3\_6456 on genome browser  
 win2\_6460 71860116 71860123 seq\_3744 205580 205586 See win2\_6460 on genome browser  
 win2\_6464 71865105 71865112 seq\_3744 210569 210575 See win2\_6464 on genome browser  
 win2\_6468 71954517 71954524 seq\_3744 299981 299987 See win2\_6468 on genome browser  
 win3\_6472 72039157 72039164 seq\_110 64732 64738 See win3\_6472 on genome browser  
 win2\_6476 72132716 72132723 seq\_110 158291 158297 See win2\_6476 on genome browser  
 win1\_6480 72247226 72247233 seq\_110 272801 272807 See win1\_6480 on genome browser  
 win3\_6484 72458566 72458573 seq\_3834 165442 165448 See win3\_6484 on genome browser  
 win2\_6488 72511005 72511012 seq\_3834 217881 217887 See win2\_6488 on genome browser  
 win2\_6492 72748585 72748592 seq\_3673 142359 142365 See win2\_6492 on genome browser  
 win3\_6496 73025062 73025069 seq\_112 109567 109573 See win3\_6496 on genome browser  
 win2\_6500 73081731 73081738 seq\_112 166236 166242 See win2\_6500 on genome browser  
 win2\_6504 73107542 73107549 seq\_112 192047 192053 See win2\_6504 on genome browser  
 win3\_6508 73117830 73117837 seq\_112 202335 202341 See win3\_6508 on genome browser  
 win1\_6512 73125309 73125316 seq\_112 209814 209820 See win1\_6512 on genome browser  
 win1\_6516 73132095 73132101 seq\_112 216600 216605 See win1\_6516 on genome browser  
 win3\_6519 73179034 73179041 seq\_112 263539 263545 See win3\_6519 on genome browser  
 win3\_6523 73185744 73185751 seq\_112 270249 270255 See win3\_6523 on genome browser  
 win2\_6527 73187127 73187134 seq\_112 271632 271638 See win2\_6527 on genome browser  
 win3\_6531 73205087 73205094 seq\_112 289592 289598 See win3\_6531 on genome browser  
 win2\_6535 73206453 73206460 seq\_112 290958 290964 See win2\_6535 on genome browser  
 win3\_6539 73224693 73224700 seq\_3671 251 257 See win3\_6539 on genome browser  
 win2\_6543 73266132 73266139 seq\_3671 41690 41696 See win2\_6543 on genome browser  
 win3\_6547 73306838 73306845 seq\_3671 82396 82402 See win3\_6547 on genome browser  
 win3\_6551 73310206 73310213 seq\_3671 85764 85770 See win3\_6551 on genome browser  
 win3\_6555 73335903 73335910 seq\_3671 111461 111467 See win3\_6555 on genome browser  
 win2\_6559 73405994 73406001 seq\_3671 181552 181558 See win2\_6559 on genome browser  
 win2\_6563 73501819 73501826 seq\_3671 277377 277383 See win2\_6563 on genome browser  
 win3\_6567 73554981 73554988 seq\_115 43072 43078 See win3\_6567 on genome browser  
 win3\_6571 73586814 73586821 seq\_115 74905 74911 See win3\_6571 on genome browser  
 win3\_6575 73601825 73601832 seq\_115 89916 89922 See win3\_6575 on genome browser  
 win2\_6579 73605538 73605545 seq\_115 93629 93635 See win2\_6579 on genome browser  
 win2\_6583 73679162 73679169 seq\_115 167253 167259 See win2\_6583 on genome browser  
 win2\_6587 73712245 73712252 seq\_115 200336 200342 See win2\_6587 on genome browser  
 win3\_6591 73718236 73718243 seq\_115 206327 206333 See win3\_6591 on genome browser  
 win3\_6595 73718456 73718463 seq\_115 206547 206553 See win3\_6595 on genome browser  
 win3\_6599 73785059 73785066 seq\_115 273150 273156 See win3\_6599 on genome browser  
 win3\_6603 73996296 73996303 seq\_3739 198136 198142 See win3\_6603 on genome browser  
 win2\_6607 74073542 74073549 seq\_3739 275382 275388 See win2\_6607 on genome browser  
 win2\_6611 74074016 74074023 seq\_3739 275856 275862 See win2\_6611 on genome browser  
 win2\_6615 74130116 74130123 seq\_117 46039 46045 See win2\_6615 on genome browser  
 win3\_6619 74130215 74130222 seq\_117 46138 46144 See win3\_6619 on genome browser  
 win3\_6623 74160657 74160664 seq\_117 76580 76586 See win3\_6623 on genome browser  
 win3\_6627 74187653 74187660 seq\_117 103576 103582 See win3\_6627 on genome browser  
 win2\_6631 74280950 74280957 seq\_117 196873 196879 See win2\_6631 on genome browser  
 win3\_6635 74286667 74286674 seq\_117 202590 202596 See win3\_6635 on genome browser  
 win3\_6639 74381209 74381216 seq\_119 12303 12309 See win3\_6639 on genome browser  
 win2\_6643 74385382 74385389 seq\_119 16476 16482 See win2\_6643 on genome browser  
 win2\_6647 74409661 74409668 seq\_119 40755 40761 See win2\_6647 on genome browser  
 win3\_6651 74638597 74638604 seq\_119 269691 269697 See win3\_6651 on genome browser  
 win3\_6655 74655951 74655958 seq\_120 4873 4879 See win3\_6655 on genome browser  
 win3\_6659 74669760 74669767 seq\_120 18682 18688 See win3\_6659 on genome browser  
 win3\_6663 74673257 74673264 seq\_120 22179 22185 See win3\_6663 on genome browser  
 win2\_6667 74691792 74691799 seq\_120 40714 40720 See win2\_6667 on genome browser  
 win2\_6671 74697171 74697178 seq\_120 46093 46099 See win2\_6671 on genome browser  
 win3\_6675 74713853 74713860 seq\_120 62775 62781 See win3\_6675 on genome browser  
 win2\_6679 74715853 74715860 seq\_120 64775 64781 See win2\_6679 on genome browser  
 win2\_6683 74727162 74727169 seq\_120 76084 76090 See win2\_6683 on genome browser  
 win3\_6687 74732163 74732170 seq\_120 81085 81091 See win3\_6687 on genome browser  
 win3\_6691 74753362 74753369 seq\_120 102284 102290 See win3\_6691 on genome browser  
 win1\_6695 74762596 74762601 seq\_120 111518 111522 See win1\_6695 on genome browser  
 win1\_6697 74771801 74771805 seq\_120 120723 120726 See win1\_6697 on genome browser  
 win3\_6698 74778675 74778682 seq\_120 127597 127603 See win3\_6698 on genome browser  
 win2\_6702 74801456 74801463 seq\_120 150378 150384 See win2\_6702 on genome browser  
 win3\_6706 74804160 74804167 seq\_120 153082 153088 See win3\_6706 on genome browser  
 win2\_6710 74811471 74811478 seq\_120 160393 160399 See win2\_6710 on genome browser  
 win3\_6714 74822241 74822248 seq\_120 171163 171169 See win3\_6714 on genome browser  
 win3\_6718 74824194 74824201 seq\_120 173116 173122 See win3\_6718 on genome browser  
 win2\_6722 74825450 74825457 seq\_120 174372 174378 See win2\_6722 on genome browser  
 win3\_6726 74829064 74829071 seq\_120 177986 177992 See win3\_6726 on genome browser  
 win1\_6730 74846872 74846877 seq\_120 195794 195798 See win1\_6730 on genome browser  
 win3\_6732 74847970 74847977 seq\_120 196892 196898 See win3\_6732 on genome browser  
 win2\_6736 74857561 74857568 seq\_120 206483 206489 See win2\_6736 on genome browser  
 win3\_6740 74862096 74862103 seq\_120 211018 211024 See win3\_6740 on genome browser  
 win2\_6744 74890754 74890761 seq\_120 239676 239682 See win2\_6744 on genome browser  
 win2\_6748 74899954 74899961 seq\_120 248876 248882 See win2\_6748 on genome browser  
 win3\_6752 74911963 74911970 seq\_120 260885 260891 See win3\_6752 on genome browser  
 win3\_6756 74914245 74914252 seq\_120 263167 263173 See win3\_6756 on genome browser  
 win2\_6760 74941499 74941506 seq\_121 8333 8339 See win2\_6760 on genome browser  
 win1\_6764 74952351 74952355 seq\_121 19185 19188 See win1\_6764 on genome browser  
 win2\_6765 74954289 74954296 seq\_121 21123 21129 See win2\_6765 on genome browser  
 win2\_6769 74991912 74991919 seq\_121 58746 58752 See win2\_6769 on genome browser  
 win1\_6773 74994089 74994093 seq\_121 60923 60926 See win1\_6773 on genome browser  
 win3\_6774 74995256 74995263 seq\_121 62090 62096 See win3\_6774 on genome browser  
 win3\_6778 75010658 75010665 seq\_121 77492 77498 See win3\_6778 on genome browser  
 win2\_6782 75010758 75010765 seq\_121 77592 77598 See win2\_6782 on genome browser  
 win1\_6786 75026552 75026559 seq\_121 93386 93392 See win1\_6786 on genome browser  
 win3\_6790 75032746 75032753 seq\_121 99580 99586 See win3\_6790 on genome browser  
 win3\_6794 75034034 75034041 seq\_121 100868 100874 See win3\_6794 on genome browser  
 win2\_6798 75038052 75038059 seq\_121 104886 104892 See win2\_6798 on genome browser  
 win2\_6802 75046197 75046204 seq\_121 113031 113037 See win2\_6802 on genome browser  
 win3\_6806 75055015 75055022 seq\_121 121849 121855 See win3\_6806 on genome browser  
 win2\_6810 75063526 75063533 seq\_121 130360 130366 See win2\_6810 on genome browser  
 win3\_6814 75108230 75108237 seq\_121 175064 175070 See win3\_6814 on genome browser  
 win3\_6818 75121593 75121600 seq\_121 188427 188433 See win3\_6818 on genome browser  
 win2\_6822 75123024 75123031 seq\_121 189858 189864 See win2\_6822 on genome browser  
 win1\_6826 75124323 75124329 seq\_121 191157 191162 See win1\_6826 on genome browser  
 win2\_6829 75125869 75125876 seq\_121 192703 192709 See win2\_6829 on genome browser  
 win3\_6833 75126565 75126572 seq\_121 193399 193405 See win3\_6833 on genome browser  
 win3\_6837 75131697 75131704 seq\_121 198531 198537 See win3\_6837 on genome browser  
 win3\_6841 75135891 75135898 seq\_121 202725 202731 See win3\_6841 on genome browser  
 win1\_6845 75136758 75136765 seq\_121 203592 203598 See win1\_6845 on genome browser  
 win2\_6849 75138191 75138198 seq\_121 205025 205031 See win2\_6849 on genome browser  
 win3\_6853 75151411 75151418 seq\_121 218245 218251 See win3\_6853 on genome browser  
 win3\_6857 75183731 75183738 seq\_121 250565 250571 See win3\_6857 on genome browser  
 win2\_6861 75186207 75186214 seq\_121 253041 253047 See win2\_6861 on genome browser  
 win3\_6865 75223546 75223553 seq\_122 11600 11606 See win3\_6865 on genome browser  
 win3\_6869 75227505 75227512 seq\_122 15559 15565 See win3\_6869 on genome browser  
 win2\_6873 75249246 75249253 seq\_122 37300 37306 See win2\_6873 on genome browser  
 win1\_6877 75253283 75253287 seq\_122 41337 41340 See win1\_6877 on genome browser  
 win3\_6878 75263664 75263671 seq\_122 51718 51724 See win3\_6878 on genome browser  
 win1\_6882 75270522 75270529 seq\_122 58576 58582 See win1\_6882 on genome browser  
 win1\_6886 75328756 75328761 seq\_122 116810 116814 See win1\_6886 on genome browser  
 win2\_6888 75330942 75330949 seq\_122 118996 119002 See win2\_6888 on genome browser  
 win2\_6892 75332475 75332482 seq\_122 120529 120535 See win2\_6892 on genome browser  
 win3\_6896 75332582 75332589 seq\_122 120636 120642 See win3\_6896 on genome browser  
 win2\_6900 75333006 75333013 seq\_122 121060 121066 See win2\_6900 on genome browser  
 win3\_6904 75334754 75334761 seq\_122 122808 122814 See win3\_6904 on genome browser  
 win2\_6908 75334851 75334858 seq\_122 122905 122911 See win2\_6908 on genome browser  
 win3\_6912 75363734 75363741 seq\_122 151788 151794 See win3\_6912 on genome browser  
 win3\_6916 75369575 75369582 seq\_122 157629 157635 See win3\_6916 on genome browser  
 win3\_6920 75376125 75376132 seq\_122 164179 164185 See win3\_6920 on genome browser  
 win2\_6924 75377534 75377541 seq\_122 165588 165594 See win2\_6924 on genome browser  
 win3\_6928 75379048 75379055 seq\_122 167102 167108 See win3\_6928 on genome browser  
 win1\_6932 75393700 75393706 seq\_122 181754 181759 See win1\_6932 on genome browser  
 win2\_6935 75424734 75424741 seq\_122 212788 212794 See win2\_6935 on genome browser  
 win2\_6939 75452848 75452855 seq\_122 240902 240908 See win2\_6939 on genome browser  
 win3\_6943 75465944 75465951 seq\_122 253998 254004 See win3\_6943 on genome browser  
 win2\_6947 75579577 75579584 seq\_3742 89704 89710 See win2\_6947 on genome browser  
 win3\_6951 75618844 75618851 seq\_3742 128971 128977 See win3\_6951 on genome browser  
 win1\_6955 75678982 75678989 seq\_3742 189109 189115 See win1\_6955 on genome browser  
 win2\_6959 75704136 75704143 seq\_3742 214263 214269 See win2\_6959 on genome browser  
 win3\_6963 75925777 75925784 seq\_3672 161866 161872 See win3\_6963 on genome browser  
 win3\_6967 76012390 76012397 seq\_3672 248479 248485 See win3\_6967 on genome browser  
 win1\_6971 76013895 76013899 seq\_3672 249984 249987 See win1\_6971 on genome browser  
 win2\_6972 76346758 76346765 seq\_3676 47555 47561 See win2\_6972 on genome browser  
 win3\_6976 76547354 76547361 seq\_3676 248151 248157 See win3\_6976 on genome browser  
 win2\_6980 76553399 76553406 seq\_3676 254196 254202 See win2\_6980 on genome browser  
 win3\_6984 76568271 76568278 seq\_127 4534 4540 See win3\_6984 on genome browser  
 win2\_6988 76614680 76614687 seq\_127 50943 50949 See win2\_6988 on genome browser  
 win2\_6992 76675847 76675854 seq\_127 112110 112116 See win2\_6992 on genome browser  
 win2\_6996 76689668 76689675 seq\_127 125931 125937 See win2\_6996 on genome browser  
 win3\_7000 76696794 76696801 seq\_127 133057 133063 See win3\_7000 on genome browser  
 win1\_7004 76699153 76699157 seq\_127 135416 135419 See win1\_7004 on genome browser  
 win3\_7005 76728226 76728233 seq\_127 164489 164495 See win3\_7005 on genome browser  
 win3\_7009 76731633 76731640 seq\_127 167896 167902 See win3\_7009 on genome browser  
 win2\_7013 76786920 76786927 seq\_127 223183 223189 See win2\_7013 on genome browser  
 win2\_7017 77017892 77017899 seq\_3738 189864 189870 See win2\_7017 on genome browser  
 win3\_7021 77146172 77146179 seq\_3755 56392 56398 See win3\_7021 on genome browser  
 win2\_7025 77247054 77247061 seq\_3755 157274 157280 See win2\_7025 on genome browser  
 win3\_7029 77351107 77351114 seq\_129 -2 4 See win3\_7029 on genome browser  
 win3\_7033 77378398 77378405 seq\_129 27289 27295 See win3\_7033 on genome browser  
 win2\_7037 77384489 77384496 seq\_129 33380 33386 See win2\_7037 on genome browser  
 win2\_7041 77389767 77389774 seq\_129 38658 38664 See win2\_7041 on genome browser  
 win1\_7045 77393056 77393062 seq\_129 41947 41952 See win1\_7045 on genome browser  
 win1\_7048 77394244 77394250 seq\_129 43135 43140 See win1\_7048 on genome browser  
 win2\_7051 77395903 77395910 seq\_129 44794 44800 See win2\_7051 on genome browser  
 win3\_7055 77429766 77429773 seq\_129 78657 78663 See win3\_7055 on genome browser  
 win3\_7059 77441522 77441529 seq\_129 90413 90419 See win3\_7059 on genome browser  
 win1\_7063 77455552 77455559 seq\_129 104443 104449 See win1\_7063 on genome browser  
 win1\_7067 77491061 77491068 seq\_129 139952 139958 See win1\_7067 on genome browser  
 win2\_7071 77504767 77504774 seq\_129 153658 153664 See win2\_7071 on genome browser  
 win2\_7075 77526177 77526184 seq\_129 175068 175074 See win2\_7075 on genome browser  
 win2\_7079 77556233 77556240 seq\_129 205124 205130 See win2\_7079 on genome browser  
 win3\_7083 77576787 77576794 seq\_129 225678 225684 See win3\_7083 on genome browser  
 win2\_7087 77579145 77579152 seq\_129 228036 228042 See win2\_7087 on genome browser  
 win1\_7091 77580755 77580761 seq\_129 229646 229651 See win1\_7091 on genome browser  
 win3\_7094 77586829 77586836 seq\_129 235720 235726 See win3\_7094 on genome browser  
 win2\_7098 77644107 77644114 seq\_133 32071 32077 See win2\_7098 on genome browser  
 win1\_7102 77707365 77707371 seq\_133 95329 95334 See win1\_7102 on genome browser  
 win2\_7105 77722370 77722377 seq\_133 110334 110340 See win2\_7105 on genome browser  
 win1\_7109 77729352 77729359 seq\_133 117316 117322 See win1\_7109 on genome browser  
 win2\_7113 77752938 77752945 seq\_133 140902 140908 See win2\_7113 on genome browser  
 win2\_7117 77778906 77778913 seq\_133 166870 166876 See win2\_7117 on genome browser  
 win3\_7121 77787377 77787384 seq\_133 175341 175347 See win3\_7121 on genome browser  
 win3\_7125 77791930 77791937 seq\_133 179894 179900 See win3\_7125 on genome browser  
 win2\_7129 77813201 77813208 seq\_133 201165 201171 See win2\_7129 on genome browser  
 win3\_7133 77947009 77947016 seq\_134 78516 78522 See win3\_7133 on genome browser  
 win2\_7137 78125449 78125456 seq\_135 1959 1965 See win2\_7137 on genome browser  
 win2\_7141 78126479 78126486 seq\_135 2989 2995 See win2\_7141 on genome browser  
 win2\_7145 78131332 78131339 seq\_135 7842 7848 See win2\_7145 on genome browser  
 win3\_7149 78134899 78134906 seq\_135 11409 11415 See win3\_7149 on genome browser  
 win2\_7153 78147609 78147616 seq\_135 24119 24125 See win2\_7153 on genome browser  
 win2\_7157 78150299 78150306 seq\_135 26809 26815 See win2\_7157 on genome browser  
 win3\_7161 78154081 78154088 seq\_135 30591 30597 See win3\_7161 on genome browser  
 win2\_7165 78157355 78157362 seq\_135 33865 33871 See win2\_7165 on genome browser  
 win2\_7169 78159971 78159978 seq\_135 36481 36487 See win2\_7169 on genome browser  
 win2\_7173 78161048 78161055 seq\_135 37558 37564 See win2\_7173 on genome browser  
 win3\_7177 78176010 78176017 seq\_135 52520 52526 See win3\_7177 on genome browser  
 win3\_7181 78187132 78187139 seq\_135 63642 63648 See win3\_7181 on genome browser  
 win3\_7185 78188408 78188415 seq\_135 64918 64924 See win3\_7185 on genome browser  
 win2\_7189 78191171 78191178 seq\_135 67681 67687 See win2\_7189 on genome browser  
 win3\_7193 78219791 78219798 seq\_135 96301 96307 See win3\_7193 on genome browser  
 win3\_7197 78244761 78244768 seq\_135 121271 121277 See win3\_7197 on genome browser  
 win3\_7201 78247314 78247321 seq\_135 123824 123830 See win3\_7201 on genome browser  
 win2\_7205 78261710 78261717 seq\_135 138220 138226 See win2\_7205 on genome browser  
 win1\_7209 78270628 78270634 seq\_135 147138 147143 See win1\_7209 on genome browser  
 win1\_7212 78273493 78273500 seq\_135 150003 150009 See win1\_7212 on genome browser  
 win2\_7216 78310758 78310765 seq\_135 187268 187274 See win2\_7216 on genome browser  
 win1\_7220 78318158 78318165 seq\_135 194668 194674 See win1\_7220 on genome browser  
 win3\_7224 78322092 78322099 seq\_135 198602 198608 See win3\_7224 on genome browser  
 win3\_7228 78323413 78323420 seq\_135 199923 199929 See win3\_7228 on genome browser  
 win3\_7232 78324723 78324730 seq\_135 201233 201239 See win3\_7232 on genome browser  
 win3\_7236 78338989 78338996 seq\_135 215499 215505 See win3\_7236 on genome browser  
 win3\_7240 78341952 78341959 seq\_135 218462 218468 See win3\_7240 on genome browser  
 win3\_7244 78345270 78345277 seq\_135 221780 221786 See win3\_7244 on genome browser  
 win3\_7248 78446414 78446421 seq\_3808 69449 69455 See win3\_7248 on genome browser  
 win3\_7252 78471478 78471485 seq\_3808 94513 94519 See win3\_7252 on genome browser  
 win2\_7256 78515612 78515619 seq\_3808 138647 138653 See win2\_7256 on genome browser  
 win3\_7260 78571285 78571292 seq\_3808 194320 194326 See win3\_7260 on genome browser  
 win3\_7264 78628573 78628580 seq\_137 -3 3 See win3\_7264 on genome browser  
 win3\_7268 78631719 78631726 seq\_137 3143 3149 See win3\_7268 on genome browser  
 win2\_7272 78631893 78631900 seq\_137 3317 3323 See win2\_7272 on genome browser  
 win1\_7276 78633883 78633888 seq\_137 5307 5311 See win1\_7276 on genome browser  
 win3\_7278 78654880 78654887 seq\_137 26304 26310 See win3\_7278 on genome browser  
 win2\_7282 78655019 78655026 seq\_137 26443 26449 See win2\_7282 on genome browser  
 win3\_7286 78665696 78665703 seq\_137 37120 37126 See win3\_7286 on genome browser  
 win2\_7290 78666167 78666174 seq\_137 37591 37597 See win2\_7290 on genome browser  
 win2\_7294 78667301 78667308 seq\_137 38725 38731 See win2\_7294 on genome browser  
 win3\_7298 78669783 78669790 seq\_137 41207 41213 See win3\_7298 on genome browser  
 win2\_7302 78681995 78682002 seq\_137 53419 53425 See win2\_7302 on genome browser  
 win2\_7306 78795420 78795427 seq\_137 166844 166850 See win2\_7306 on genome browser  
 win2\_7310 78799414 78799421 seq\_137 170838 170844 See win2\_7310 on genome browser  
 win2\_7314 78802769 78802776 seq\_137 174193 174199 See win2\_7314 on genome browser  
 win3\_7318 78820342 78820349 seq\_137 191766 191772 See win3\_7318 on genome browser  
 win2\_7322 78858086 78858093 seq\_137 229510 229516 See win2\_7322 on genome browser  
 win2\_7326 78860309 78860316 seq\_137 231733 231739 See win2\_7326 on genome browser  
 win2\_7330 78904051 78904058 seq\_138 29964 29970 See win2\_7330 on genome browser  
 win3\_7334 78904150 78904157 seq\_138 30063 30069 See win3\_7334 on genome browser  
 win3\_7338 78941813 78941820 seq\_138 67726 67732 See win3\_7338 on genome browser  
 win3\_7342 78990958 78990965 seq\_138 116871 116877 See win3\_7342 on genome browser  
 win2\_7346 79077642 79077649 seq\_138 203555 203561 See win2\_7346 on genome browser  
 win3\_7350 79094888 79094895 seq\_138 220801 220807 See win3\_7350 on genome browser  
 win2\_7354 79139865 79139872 seq\_3759 22257 22263 See win2\_7354 on genome browser  
 win1\_7358 79165152 79165157 seq\_3759 47544 47548 See win1\_7358 on genome browser  
 win2\_7360 79242785 79242792 seq\_3759 125177 125183 See win2\_7360 on genome browser  
 win2\_7364 79244095 79244102 seq\_3759 126487 126493 See win2\_7364 on genome browser  
 win2\_7368 79246616 79246623 seq\_3759 129008 129014 See win2\_7368 on genome browser  
 win2\_7372 79252438 79252445 seq\_3759 134830 134836 See win2\_7372 on genome browser  
 win2\_7376 79328905 79328912 seq\_3759 211297 211303 See win2\_7376 on genome browser  
 win2\_7380 79334006 79334013 seq\_3759 216398 216404 See win2\_7380 on genome browser  
 win3\_7384 79393229 79393236 seq\_139 32477 32483 See win3\_7384 on genome browser  
 win2\_7388 79442519 79442526 seq\_139 81767 81773 See win2\_7388 on genome browser  
 win3\_7392 79442642 79442649 seq\_139 81890 81896 See win3\_7392 on genome browser  
 win3\_7396 79464491 79464498 seq\_139 103739 103745 See win3\_7396 on genome browser  
 win1\_7400 79466733 79466740 seq\_139 105981 105987 See win1\_7400 on genome browser  
 win3\_7404 79471439 79471446 seq\_139 110687 110693 See win3\_7404 on genome browser  
 win2\_7408 79476151 79476158 seq\_139 115399 115405 See win2\_7408 on genome browser  
 win3\_7412 79512064 79512071 seq\_139 151312 151318 See win3\_7412 on genome browser  
 win1\_7416 79518498 79518504 seq\_139 157746 157751 See win1\_7416 on genome browser  
 win3\_7419 79520716 79520723 seq\_139 159964 159970 See win3\_7419 on genome browser  
 win2\_7423 79520861 79520866 seq\_139 160109 160113 See win2\_7423 on genome browser  
 win3\_7425 79583561 79583568 seq\_139 222809 222815 See win3\_7425 on genome browser  
 win2\_7429 79696256 79696263 seq\_3682 93376 93382 See win2\_7429 on genome browser  
 win3\_7433 79833284 79833291 seq\_3682 230404 230410 See win3\_7433 on genome browser  
 win2\_7437 79987830 79987837 seq\_3683 144011 144017 See win2\_7437 on genome browser  
 win2\_7441 80095376 80095383 seq\_143 10626 10632 See win2\_7441 on genome browser  
 win3\_7445 80099732 80099739 seq\_143 14982 14988 See win3\_7445 on genome browser  
 win2\_7449 80099899 80099906 seq\_143 15149 15155 See win2\_7449 on genome browser  
 win3\_7453 80111058 80111065 seq\_143 26308 26314 See win3\_7453 on genome browser  
 win2\_7457 80202723 80202730 seq\_143 117973 117979 See win2\_7457 on genome browser  
 win3\_7461 80296180 80296187 seq\_143 211430 211436 See win3\_7461 on genome browser  
 win1\_7465 80324566 80324570 seq\_144 3791 3794 See win1\_7465 on genome browser  
 win2\_7466 80325877 80325884 seq\_144 5102 5108 See win2\_7466 on genome browser  
 win3\_7470 80360328 80360335 seq\_144 39553 39559 See win3\_7470 on genome browser  
 win2\_7474 80383122 80383129 seq\_144 62347 62353 See win2\_7474 on genome browser  
 win3\_7478 80417118 80417125 seq\_144 96343 96349 See win3\_7478 on genome browser  
 win3\_7482 80418191 80418198 seq\_144 97416 97422 See win3\_7482 on genome browser  
 win3\_7486 80429315 80429322 seq\_144 108540 108546 See win3\_7486 on genome browser  
 win1\_7490 80430152 80430157 seq\_144 109377 109381 See win1\_7490 on genome browser  
 win3\_7492 80432510 80432517 seq\_144 111735 111741 See win3\_7492 on genome browser  
 win2\_7496 80456412 80456419 seq\_144 135637 135643 See win2\_7496 on genome browser  
 win3\_7500 80493357 80493364 seq\_144 172582 172588 See win3\_7500 on genome browser  
 win1\_7504 80493424 80493430 seq\_144 172649 172654 See win1\_7504 on genome browser  
 win2\_7507 80500352 80500359 seq\_144 179577 179583 See win2\_7507 on genome browser  
 win1\_7511 80528276 80528280 seq\_144 207501 207504 See win1\_7511 on genome browser  
 win1\_7512 80541141 80541147 seq\_144 220366 220371 See win1\_7512 on genome browser  
 win3\_7515 80542212 80542219 seq\_144 221437 221443 See win3\_7515 on genome browser  
 win1\_7519 80575322 80575328 seq\_3743 18678 18683 See win1\_7519 on genome browser  
 win3\_7522 80579452 80579459 seq\_3743 22808 22814 See win3\_7522 on genome browser  
 win2\_7526 80647297 80647303 seq\_3743 90653 90658 See win2\_7526 on genome browser  
 win2\_7529 80717572 80717579 seq\_3743 160928 160934 See win2\_7529 on genome browser  
 win3\_7533 80807189 80807196 seq\_145 15237 15243 See win3\_7533 on genome browser  
 win2\_7537 80808172 80808179 seq\_145 16220 16226 See win2\_7537 on genome browser  
 win3\_7541 80853597 80853604 seq\_145 61645 61651 See win3\_7541 on genome browser  
 win3\_7545 80898572 80898579 seq\_145 106620 106626 See win3\_7545 on genome browser  
 win2\_7549 80905017 80905024 seq\_145 113065 113071 See win2\_7549 on genome browser  
 win2\_7553 80907928 80907935 seq\_145 115976 115982 See win2\_7553 on genome browser  
 win3\_7557 80931436 80931443 seq\_145 139484 139490 See win3\_7557 on genome browser  
 win3\_7561 80944295 80944302 seq\_145 152343 152349 See win3\_7561 on genome browser  
 win3\_7565 80949352 80949359 seq\_145 157400 157406 See win3\_7565 on genome browser  
 win2\_7569 80954261 80954268 seq\_145 162309 162315 See win2\_7569 on genome browser  
 win3\_7573 80958257 80958264 seq\_145 166305 166311 See win3\_7573 on genome browser  
 win1\_7577 80959576 80959582 seq\_145 167624 167629 See win1\_7577 on genome browser  
 win2\_7580 80961408 80961415 seq\_145 169456 169462 See win2\_7580 on genome browser  
 win2\_7584 80971010 80971017 seq\_145 179058 179064 See win2\_7584 on genome browser  
 win3\_7588 80999542 80999549 seq\_145 207590 207596 See win3\_7588 on genome browser  
 win2\_7592 81012218 81012225 seq\_145 220266 220272 See win2\_7592 on genome browser  
 win2\_7596 81097797 81097804 seq\_3741 71101 71107 See win2\_7596 on genome browser  
 win3\_7600 81103309 81103316 seq\_3741 76613 76619 See win3\_7600 on genome browser  
 win2\_7604 81433116 81433123 seq\_3750 179438 179444 See win2\_7604 on genome browser  
 win2\_7608 81467116 81467123 seq\_3750 213438 213444 See win2\_7608 on genome browser  
 win2\_7612 81574745 81574752 seq\_148 97277 97283 See win2\_7612 on genome browser  
 win3\_7616 81602996 81603001 seq\_148 125528 125532 See win3\_7616 on genome browser  
 win2\_7618 81603066 81603073 seq\_148 125598 125604 See win2\_7618 on genome browser  
 win3\_7622 81606827 81606834 seq\_148 129359 129365 See win3\_7622 on genome browser  
 win1\_7626 81668728 81668735 seq\_148 191260 191266 See win1\_7626 on genome browser  
 win1\_7630 81706740 81706745 seq\_151 6726 6730 See win1\_7630 on genome browser  
 win3\_7632 81708041 81708048 seq\_151 8027 8033 See win3\_7632 on genome browser  
 win3\_7636 81760862 81760869 seq\_151 60848 60854 See win3\_7636 on genome browser  
 win2\_7640 81773318 81773325 seq\_151 73304 73310 See win2\_7640 on genome browser  
 win2\_7644 81785585 81785592 seq\_151 85571 85577 See win2\_7644 on genome browser  
 win3\_7648 81818890 81818897 seq\_151 118876 118882 See win3\_7648 on genome browser  
 win3\_7652 81851325 81851332 seq\_151 151311 151317 See win3\_7652 on genome browser  
 win2\_7656 81852501 81852508 seq\_151 152487 152493 See win2\_7656 on genome browser  
 win1\_7660 81858600 81858605 seq\_151 158586 158590 See win1\_7660 on genome browser  
 win3\_7662 81863099 81863106 seq\_151 163085 163091 See win3\_7662 on genome browser  
 win3\_7666 81878893 81878900 seq\_151 178879 178885 See win3\_7666 on genome browser  
 win2\_7670 81891291 81891298 seq\_151 191277 191283 See win2\_7670 on genome browser  
 win3\_7674 81897378 81897385 seq\_151 197364 197370 See win3\_7674 on genome browser  
 win3\_7678 81904633 81904640 seq\_151 204619 204625 See win3\_7678 on genome browser  
 win2\_7682 81908274 81908281 seq\_151 208260 208266 See win2\_7682 on genome browser  
 win2\_7686 81957831 81957838 seq\_152 37454 37460 See win2\_7686 on genome browser  
 win2\_7690 82032039 82032046 seq\_152 111662 111668 See win2\_7690 on genome browser  
 win1\_7694 82149495 82149500 seq\_3751 9491 9495 See win1\_7694 on genome browser  
 win3\_7696 82221775 82221782 seq\_3751 81771 81777 See win3\_7696 on genome browser  
 win2\_7700 82222392 82222399 seq\_3751 82388 82394 See win2\_7700 on genome browser  
 win3\_7704 82301044 82301051 seq\_3751 161040 161046 See win3\_7704 on genome browser  
 win2\_7708 82306403 82306410 seq\_3751 166399 166405 See win2\_7708 on genome browser  
 win3\_7712 82380739 82380746 seq\_153 23896 23902 See win3\_7712 on genome browser  
 win2\_7716 82382723 82382730 seq\_153 25880 25886 See win2\_7716 on genome browser  
 win2\_7720 82382828 82382835 seq\_153 25985 25991 See win2\_7720 on genome browser  
 win2\_7724 82396975 82396982 seq\_153 40132 40138 See win2\_7724 on genome browser  
 win2\_7728 82397167 82397174 seq\_153 40324 40330 See win2\_7728 on genome browser  
 win3\_7732 82404214 82404221 seq\_153 47371 47377 See win3\_7732 on genome browser  
 win2\_7736 82407445 82407452 seq\_153 50602 50608 See win2\_7736 on genome browser  
 win3\_7740 82436235 82436242 seq\_153 79392 79398 See win3\_7740 on genome browser  
 win2\_7744 82438306 82438313 seq\_153 81463 81469 See win2\_7744 on genome browser  
 win3\_7748 82455641 82455648 seq\_153 98798 98804 See win3\_7748 on genome browser  
 win3\_7752 82464980 82464987 seq\_153 108137 108143 See win3\_7752 on genome browser  
 win2\_7756 82467354 82467362 seq\_153 110511 110518 See win2\_7756 on genome browser  
 win2\_7761 82469740 82469747 seq\_153 112897 112903 See win2\_7761 on genome browser  
 win3\_7765 82474456 82474463 seq\_153 117613 117619 See win3\_7765 on genome browser  
 win3\_7769 82507338 82507345 seq\_153 150495 150501 See win3\_7769 on genome browser  
 win2\_7773 82514588 82514595 seq\_153 157745 157751 See win2\_7773 on genome browser  
 win2\_7777 82544214 82544221 seq\_153 187371 187377 See win2\_7777 on genome browser  
 win3\_7781 82559412 82559419 seq\_153 202569 202575 See win3\_7781 on genome browser  
 win2\_7785 82559611 82559618 seq\_153 202768 202774 See win2\_7785 on genome browser  
 win2\_7789 82575788 82575795 seq\_155 2212 2218 See win2\_7789 on genome browser  
 win2\_7793 82579484 82579491 seq\_155 5908 5914 See win2\_7793 on genome browser  
 win2\_7797 82580575 82580582 seq\_155 6999 7005 See win2\_7797 on genome browser  
 win1\_7801 82582286 82582292 seq\_155 8710 8715 See win1\_7801 on genome browser  
 win3\_7804 82586604 82586611 seq\_155 13028 13034 See win3\_7804 on genome browser  
 win2\_7808 82594861 82594868 seq\_155 21285 21291 See win2\_7808 on genome browser  
 win2\_7812 82602707 82602714 seq\_155 29131 29137 See win2\_7812 on genome browser  
 win2\_7816 82606619 82606626 seq\_155 33043 33049 See win2\_7816 on genome browser  
 win2\_7820 82643850 82643857 seq\_155 70274 70280 See win2\_7820 on genome browser  
 win3\_7824 82643957 82643964 seq\_155 70381 70387 See win3\_7824 on genome browser  
 win3\_7828 82644595 82644602 seq\_155 71019 71025 See win3\_7828 on genome browser  
 win2\_7832 82646793 82646800 seq\_155 73217 73223 See win2\_7832 on genome browser  
 win3\_7836 82648592 82648599 seq\_155 75016 75022 See win3\_7836 on genome browser  
 win3\_7840 82658319 82658326 seq\_155 84743 84749 See win3\_7840 on genome browser  
 win3\_7844 82658457 82658464 seq\_155 84881 84887 See win3\_7844 on genome browser  
 win3\_7848 82660627 82660634 seq\_155 87051 87057 See win3\_7848 on genome browser  
 win1\_7852 82681879 82681885 seq\_155 108303 108308 See win1\_7852 on genome browser  
 win2\_7855 82689548 82689555 seq\_155 115972 115978 See win2\_7855 on genome browser  
 win1\_7859 82722544 82722551 seq\_155 148968 148974 See win1\_7859 on genome browser  
 win3\_7863 82729746 82729753 seq\_155 156170 156176 See win3\_7863 on genome browser  
 win2\_7867 82732306 82732313 seq\_155 158730 158736 See win2\_7867 on genome browser  
 win2\_7871 82752408 82752415 seq\_155 178832 178838 See win2\_7871 on genome browser  
 win3\_7875 82764734 82764741 seq\_155 191158 191164 See win3\_7875 on genome browser  
 win2\_7879 82766075 82766082 seq\_155 192499 192505 See win2\_7879 on genome browser  
 win3\_7883 82774805 82774812 seq\_155 201229 201235 See win3\_7883 on genome browser  
 win3\_7887 82774909 82774916 seq\_155 201333 201339 See win3\_7887 on genome browser  
 win3\_7891 82864553 82864560 seq\_156 77461 77467 See win3\_7891 on genome browser  
 win3\_7895 82867887 82867894 seq\_156 80795 80801 See win3\_7895 on genome browser  
 win3\_7899 82879976 82879983 seq\_156 92884 92890 See win3\_7899 on genome browser  
 win3\_7903 82902674 82902681 seq\_156 115582 115588 See win3\_7903 on genome browser  
 win1\_7907 82904168 82904175 seq\_156 117076 117082 See win1\_7907 on genome browser  
 win2\_7911 82907373 82907380 seq\_156 120281 120287 See win2\_7911 on genome browser  
 win1\_7915 82917039 82917046 seq\_156 129947 129953 See win1\_7915 on genome browser  
 win2\_7919 82919660 82919667 seq\_156 132568 132574 See win2\_7919 on genome browser  
 win3\_7923 82932887 82932894 seq\_156 145795 145801 See win3\_7923 on genome browser  
 win1\_7927 82933109 82933116 seq\_156 146017 146023 See win1\_7927 on genome browser  
 win2\_7931 82933209 82933216 seq\_156 146117 146123 See win2\_7931 on genome browser  
 win1\_7935 82938876 82938880 seq\_156 151784 151787 See win1\_7935 on genome browser  
 win1\_7936 82943978 82943983 seq\_156 156886 156890 See win1\_7936 on genome browser  
 win3\_7938 82954103 82954110 seq\_156 167011 167017 See win3\_7938 on genome browser  
 win3\_7942 82978973 82978980 seq\_156 191881 191887 See win3\_7942 on genome browser  
 win3\_7946 83015658 83015665 seq\_158 16081 16087 See win3\_7946 on genome browser  
 win3\_7950 83020549 83020556 seq\_158 20972 20978 See win3\_7950 on genome browser  
 win3\_7954 83031805 83031812 seq\_158 32228 32234 See win3\_7954 on genome browser  
 win3\_7958 83033898 83033905 seq\_158 34321 34327 See win3\_7958 on genome browser  
 win3\_7962 83058277 83058284 seq\_158 58700 58706 See win3\_7962 on genome browser  
 win3\_7966 83080566 83080573 seq\_158 80989 80995 See win3\_7966 on genome browser  
 win3\_7970 83141021 83141028 seq\_158 141444 141450 See win3\_7970 on genome browser  
 win3\_7974 83182473 83182480 seq\_158 182896 182902 See win3\_7974 on genome browser  
 win2\_7978 83187231 83187238 seq\_158 187654 187660 See win2\_7978 on genome browser  
 win3\_7982 83196608 83196615 seq\_158 197031 197037 See win3\_7982 on genome browser  
 win2\_7986 83197471 83197478 seq\_158 197894 197900 See win2\_7986 on genome browser  
 win2\_7990 83353825 83353832 seq\_159 146083 146089 See win2\_7990 on genome browser  
 win2\_7994 83414516 83414523 seq\_160 378 384 See win2\_7994 on genome browser  
 win2\_7998 83417602 83417609 seq\_160 3464 3470 See win2\_7998 on genome browser  
 win3\_8002 83417701 83417708 seq\_160 3563 3569 See win3\_8002 on genome browser  
 win2\_8006 83421748 83421755 seq\_160 7610 7616 See win2\_8006 on genome browser  
 win3\_8010 83434069 83434076 seq\_160 19931 19937 See win3\_8010 on genome browser  
 win1\_8014 83436172 83436178 seq\_160 22034 22039 See win1\_8014 on genome browser  
 win3\_8017 83454452 83454459 seq\_160 40314 40320 See win3\_8017 on genome browser  
 win1\_8021 83464298 83464305 seq\_160 50160 50166 See win1\_8021 on genome browser  
 win3\_8026 83486056 83486063 seq\_160 71918 71924 See win3\_8026 on genome browser  
 win3\_8030 83497992 83497999 seq\_160 83854 83860 See win3\_8030 on genome browser  
 win3\_8034 83505989 83505996 seq\_160 91851 91857 See win3\_8034 on genome browser  
 win3\_8038 83575980 83575987 seq\_160 161842 161848 See win3\_8038 on genome browser  
 win3\_8042 83580936 83580943 seq\_160 166798 166804 See win3\_8042 on genome browser  
 win3\_8046 83582204 83582209 seq\_160 168066 168070 See win3\_8046 on genome browser  
 win2\_8048 83582366 83582373 seq\_160 168228 168234 See win2\_8048 on genome browser  
 win3\_8052 83582388 83582393 seq\_160 168250 168254 See win3\_8052 on genome browser  
 win2\_8054 83582595 83582602 seq\_160 168457 168463 See win2\_8054 on genome browser  
 win2\_8058 83609138 83609145 seq\_160 195000 195006 See win2\_8058 on genome browser  
 win3\_8062 83640355 83640362 seq\_161 20231 20237 See win3\_8062 on genome browser  
 win2\_8066 83649378 83649385 seq\_161 29254 29260 See win2\_8066 on genome browser  
 win1\_8070 83659165 83659172 seq\_161 39041 39047 See win1\_8070 on genome browser  
 win3\_8074 83661395 83661402 seq\_161 41271 41277 See win3\_8074 on genome browser  
 win2\_8078 83711015 83711022 seq\_161 90891 90897 See win2\_8078 on genome browser  
 win2\_8082 83711114 83711121 seq\_161 90990 90996 See win2\_8082 on genome browser  
 win2\_8086 83712756 83712763 seq\_161 92632 92638 See win2\_8086 on genome browser  
 win3\_8090 83717710 83717717 seq\_161 97586 97592 See win3\_8090 on genome browser  
 win3\_8094 83751163 83751170 seq\_161 131039 131045 See win3\_8094 on genome browser  
 win3\_8098 83752111 83752118 seq\_161 131987 131993 See win3\_8098 on genome browser  
 win1\_8102 83757370 83757377 seq\_161 137246 137252 See win1\_8102 on genome browser  
 win3\_8106 83766291 83766298 seq\_161 146167 146173 See win3\_8106 on genome browser  
 win2\_8110 83768707 83768714 seq\_161 148583 148589 See win2\_8110 on genome browser  
 win2\_8114 83785387 83785394 seq\_161 165263 165269 See win2\_8114 on genome browser  
 win3\_8118 83789818 83789825 seq\_161 169694 169700 See win3\_8118 on genome browser  
 win2\_8122 83790391 83790398 seq\_161 170267 170273 See win2\_8122 on genome browser  
 win3\_8126 83790824 83790831 seq\_161 170700 170706 See win3\_8126 on genome browser  
 win3\_8130 83791482 83791489 seq\_161 171358 171364 See win3\_8130 on genome browser  
 win3\_8134 83795707 83795714 seq\_161 175583 175589 See win3\_8134 on genome browser  
 win2\_8138 83796691 83796698 seq\_161 176567 176573 See win2\_8138 on genome browser  
 win1\_8142 83799937 83799943 seq\_161 179813 179818 See win1\_8142 on genome browser  
 win3\_8145 83843059 83843066 seq\_162 17828 17834 See win3\_8145 on genome browser  
 win3\_8149 83866716 83866723 seq\_162 41485 41491 See win3\_8149 on genome browser  
 win2\_8153 83882800 83882807 seq\_162 57569 57575 See win2\_8153 on genome browser  
 win3\_8157 83918742 83918749 seq\_162 93511 93517 See win3\_8157 on genome browser  
 win1\_8161 83982789 83982796 seq\_162 157558 157564 See win1\_8161 on genome browser  
 win3\_8165 83991005 83991012 seq\_162 165774 165780 See win3\_8165 on genome browser  
 win3\_8169 83997517 83997524 seq\_162 172286 172292 See win3\_8169 on genome browser  
 win2\_8173 83997773 83997780 seq\_162 172542 172548 See win2\_8173 on genome browser  
 win2\_8177 84014446 84014453 seq\_162 189215 189221 See win2\_8177 on genome browser  
 win3\_8181 84056004 84056011 seq\_3754 25809 25815 See win3\_8181 on genome browser  
 win3\_8185 84056090 84056095 seq\_3754 25895 25899 See win3\_8185 on genome browser  
 win1\_8188 84093927 84093934 seq\_3754 63732 63738 See win1\_8188 on genome browser  
 win2\_8192 84171730 84171737 seq\_3754 141535 141541 See win2\_8192 on genome browser  
 win2\_8196 84213368 84213375 seq\_3754 183173 183179 See win2\_8196 on genome browser  
 win3\_8200 84325384 84325391 seq\_164 91268 91274 See win3\_8200 on genome browser  
 win3\_8204 84393513 84393520 seq\_164 159397 159403 See win3\_8204 on genome browser  
 win3\_8208 84405128 84405135 seq\_164 171012 171018 See win3\_8208 on genome browser  
 win1\_8212 84512231 84512238 seq\_3747 75852 75858 See win1\_8212 on genome browser  
 win2\_8216 84521327 84521334 seq\_3747 84948 84954 See win2\_8216 on genome browser  
 win3\_8220 84639594 84639601 seq\_166 1724 1730 See win3\_8220 on genome browser  
 win2\_8224 84657476 84657483 seq\_166 19606 19612 See win2\_8224 on genome browser  
 win2\_8228 84701247 84701254 seq\_166 63377 63383 See win2\_8228 on genome browser  
 win2\_8232 84721077 84721084 seq\_166 83207 83213 See win2\_8232 on genome browser  
 win3\_8236 84764736 84764743 seq\_166 126866 126872 See win3\_8236 on genome browser  
 win2\_8240 84805707 84805714 seq\_166 167837 167843 See win2\_8240 on genome browser  
 win1\_8244 84822201 84822208 seq\_166 184331 184337 See win1\_8244 on genome browser  
 win2\_8248 84824947 84824954 seq\_166 187077 187083 See win2\_8248 on genome browser  
 win3\_8252 84835210 84835217 seq\_3752 262 268 See win3\_8252 on genome browser  
 win3\_8256 84899821 84899828 seq\_3752 64873 64879 See win3\_8256 on genome browser  
 win3\_8260 84968718 84968725 seq\_3752 133770 133776 See win3\_8260 on genome browser  
 win2\_8264 84995870 84995877 seq\_3752 160922 160928 See win2\_8264 on genome browser  
 win2\_8268 85018168 85018175 seq\_3752 183220 183226 See win2\_8268 on genome browser  
 win3\_8272 85031040 85031047 seq\_3745 351 357 See win3\_8272 on genome browser  
 win3\_8276 85037841 85037848 seq\_3745 7152 7158 See win3\_8276 on genome browser  
 win3\_8280 85232337 85232344 seq\_169 8533 8539 See win3\_8280 on genome browser  
 win2\_8284 85232477 85232484 seq\_169 8673 8679 See win2\_8284 on genome browser  
 win3\_8288 85241647 85241654 seq\_169 17843 17849 See win3\_8288 on genome browser  
 win2\_8292 85242376 85242383 seq\_169 18572 18578 See win2\_8292 on genome browser  
 win1\_8296 85246801 85246808 seq\_169 22997 23003 See win1\_8296 on genome browser  
 win2\_8300 85249721 85249728 seq\_169 25917 25923 See win2\_8300 on genome browser  
 win1\_8304 85250865 85250872 seq\_169 27061 27067 See win1\_8304 on genome browser  
 win3\_8308 85255706 85255713 seq\_169 31902 31908 See win3\_8308 on genome browser  
 win2\_8312 85278554 85278561 seq\_169 54750 54756 See win2\_8312 on genome browser  
 win2\_8316 85284754 85284761 seq\_169 60950 60956 See win2\_8316 on genome browser  
 win3\_8320 85288560 85288567 seq\_169 64756 64762 See win3\_8320 on genome browser  
 win2\_8324 85318697 85318704 seq\_169 94893 94899 See win2\_8324 on genome browser  
 win2\_8328 85322165 85322172 seq\_169 98361 98367 See win2\_8328 on genome browser  
 win3\_8332 85338003 85338010 seq\_169 114199 114205 See win3\_8332 on genome browser  
 win2\_8336 85344197 85344204 seq\_169 120393 120399 See win2\_8336 on genome browser  
 win3\_8340 85363307 85363314 seq\_169 139503 139509 See win3\_8340 on genome browser  
 win3\_8344 85371408 85371415 seq\_169 147604 147610 See win3\_8344 on genome browser  
 win3\_8348 85372748 85372755 seq\_169 148944 148950 See win3\_8348 on genome browser  
 win1\_8352 85400125 85400132 seq\_169 176321 176327 See win1\_8352 on genome browser  
 win2\_8356 85591031 85591038 seq\_170 177115 177121 See win2\_8356 on genome browser  
 win3\_8360 85685923 85685930 seq\_171 84087 84093 See win3\_8360 on genome browser  
 win3\_8364 85710827 85710834 seq\_171 108991 108997 See win3\_8364 on genome browser  
 win3\_8368 85727679 85727686 seq\_171 125843 125849 See win3\_8368 on genome browser  
 win2\_8372 85737285 85737292 seq\_171 135449 135455 See win2\_8372 on genome browser  
 win3\_8376 85768002 85768009 seq\_171 166166 166172 See win3\_8376 on genome browser  
 win3\_8380 85822501 85822508 seq\_172 36931 36937 See win3\_8380 on genome browser  
 win2\_8384 85844513 85844520 seq\_172 58943 58949 See win2\_8384 on genome browser  
 win2\_8388 85859285 85859292 seq\_172 73715 73721 See win2\_8388 on genome browser  
 win3\_8392 85906435 85906442 seq\_172 120865 120871 See win3\_8392 on genome browser  
 win3\_8396 85952038 85952045 seq\_172 166468 166474 See win3\_8396 on genome browser  
 win3\_8400 85952169 85952176 seq\_172 166599 166605 See win3\_8400 on genome browser  
 win3\_8404 85955737 85955744 seq\_172 170167 170173 See win3\_8404 on genome browser  
 win3\_8408 85957871 85957878 seq\_172 172301 172307 See win3\_8408 on genome browser  
 win2\_8412 85992945 85992952 seq\_173 23954 23960 See win2\_8412 on genome browser  
 win2\_8416 86041335 86041342 seq\_173 72344 72350 See win2\_8416 on genome browser  
 win2\_8420 86111328 86111335 seq\_173 142337 142343 See win2\_8420 on genome browser  
 win2\_8424 86128551 86128558 seq\_173 159560 159566 See win2\_8424 on genome browser  
 win2\_8428 86304608 86304615 seq\_3678 155798 155804 See win2\_8428 on genome browser  
 win3\_8432 86344610 86344617 seq\_175 17466 17472 See win3\_8432 on genome browser  
 win2\_8436 86349196 86349203 seq\_175 22052 22058 See win2\_8436 on genome browser  
 win2\_8440 86374077 86374084 seq\_175 46933 46939 See win2\_8440 on genome browser  
 win3\_8444 86378422 86378429 seq\_175 51278 51284 See win3\_8444 on genome browser  
 win3\_8448 86386559 86386566 seq\_175 59415 59421 See win3\_8448 on genome browser  
 win2\_8452 86388781 86388788 seq\_175 61637 61643 See win2\_8452 on genome browser  
 win3\_8456 86401964 86401971 seq\_175 74820 74826 See win3\_8456 on genome browser  
 win3\_8460 86415304 86415311 seq\_175 88160 88166 See win3\_8460 on genome browser  
 win2\_8464 86420659 86420666 seq\_175 93515 93521 See win2\_8464 on genome browser  
 win2\_8468 86424261 86424268 seq\_175 97117 97123 See win2\_8468 on genome browser  
 win3\_8472 86463097 86463104 seq\_175 135953 135959 See win3\_8472 on genome browser  
 win3\_8476 86523448 86523455 seq\_176 21957 21963 See win3\_8476 on genome browser  
 win3\_8480 86562817 86562824 seq\_176 61326 61332 See win3\_8480 on genome browser  
 win3\_8484 86567325 86567332 seq\_176 65834 65840 See win3\_8484 on genome browser  
 win3\_8488 86567536 86567543 seq\_176 66045 66051 See win3\_8488 on genome browser  
 win3\_8492 86575420 86575427 seq\_176 73929 73935 See win3\_8492 on genome browser  
 win2\_8496 86609549 86609556 seq\_176 108058 108064 See win2\_8496 on genome browser  
 win3\_8500 86645705 86645712 seq\_176 144214 144220 See win3\_8500 on genome browser  
 win2\_8504 86661200 86661207 seq\_176 159709 159715 See win2\_8504 on genome browser  
 win2\_8508 86663223 86663230 seq\_176 161732 161738 See win2\_8508 on genome browser  
 win1\_8512 86683906 86683912 seq\_177 8118 8123 See win1\_8512 on genome browser  
 win3\_8515 86694819 86694826 seq\_177 19031 19037 See win3\_8515 on genome browser  
 win3\_8519 86696170 86696177 seq\_177 20382 20388 See win3\_8519 on genome browser  
 win2\_8523 86705825 86705832 seq\_177 30037 30043 See win2\_8523 on genome browser  
 win3\_8527 86706038 86706045 seq\_177 30250 30256 See win3\_8527 on genome browser  
 win3\_8531 86723840 86723847 seq\_177 48052 48058 See win3\_8531 on genome browser  
 win2\_8535 86724439 86724446 seq\_177 48651 48657 See win2\_8535 on genome browser  
 win2\_8539 86734590 86734597 seq\_177 58802 58808 See win2\_8539 on genome browser  
 win3\_8543 86736409 86736416 seq\_177 60621 60627 See win3\_8543 on genome browser  
 win3\_8547 86739638 86739645 seq\_177 63850 63856 See win3\_8547 on genome browser  
 win3\_8551 86746176 86746183 seq\_177 70388 70394 See win3\_8551 on genome browser  
 win3\_8555 86775087 86775094 seq\_177 99299 99305 See win3\_8555 on genome browser  
 win2\_8559 86776554 86776561 seq\_177 100766 100772 See win2\_8559 on genome browser  
 win2\_8563 86779903 86779910 seq\_177 104115 104121 See win2\_8563 on genome browser  
 win2\_8567 86784860 86784867 seq\_177 109072 109078 See win2\_8567 on genome browser  
 win3\_8571 86791634 86791641 seq\_177 115846 115852 See win3\_8571 on genome browser  
 win3\_8575 86809606 86809613 seq\_177 133818 133824 See win3\_8575 on genome browser  
 win3\_8579 86812829 86812836 seq\_177 137041 137047 See win3\_8579 on genome browser  
 win3\_8583 86870665 86870672 seq\_178 20714 20720 See win3\_8583 on genome browser  
 win3\_8587 86874143 86874150 seq\_178 24192 24198 See win3\_8587 on genome browser  
 win3\_8591 86875171 86875178 seq\_178 25220 25226 See win3\_8591 on genome browser  
 win3\_8595 86876911 86876918 seq\_178 26960 26966 See win3\_8595 on genome browser  
 win3\_8599 86879089 86879096 seq\_178 29138 29144 See win3\_8599 on genome browser  
 win3\_8603 86903897 86903904 seq\_178 53946 53952 See win3\_8603 on genome browser  
 win3\_8607 86932931 86932938 seq\_178 82980 82986 See win3\_8607 on genome browser  
 win2\_8611 86966899 86966906 seq\_178 116948 116954 See win2\_8611 on genome browser  
 win2\_8615 86974093 86974100 seq\_178 124142 124148 See win2\_8615 on genome browser  
 win3\_8619 86987025 86987032 seq\_178 137074 137080 See win3\_8619 on genome browser  
 win2\_8623 87024587 87024594 seq\_179 1488 1494 See win2\_8623 on genome browser  
 win2\_8627 87042802 87042809 seq\_179 19703 19709 See win2\_8627 on genome browser  
 win3\_8631 87043268 87043275 seq\_179 20169 20175 See win3\_8631 on genome browser  
 win2\_8635 87043408 87043415 seq\_179 20309 20315 See win2\_8635 on genome browser  
 win3\_8639 87046888 87046895 seq\_179 23789 23795 See win3\_8639 on genome browser  
 win2\_8643 87047029 87047036 seq\_179 23930 23936 See win2\_8643 on genome browser  
 win3\_8647 87049417 87049424 seq\_179 26318 26324 See win3\_8647 on genome browser  
 win1\_8651 87051604 87051609 seq\_179 28505 28509 See win1\_8651 on genome browser  
 win1\_8653 87053494 87053498 seq\_179 30395 30398 See win1\_8653 on genome browser  
 win3\_8654 87060350 87060357 seq\_179 37251 37257 See win3\_8654 on genome browser  
 win3\_8658 87064170 87064177 seq\_179 41071 41077 See win3\_8658 on genome browser  
 win2\_8662 87065324 87065331 seq\_179 42225 42231 See win2\_8662 on genome browser  
 win3\_8666 87090946 87090953 seq\_179 67847 67853 See win3\_8666 on genome browser  
 win2\_8670 87097444 87097451 seq\_179 74345 74351 See win2\_8670 on genome browser  
 win3\_8674 87100594 87100601 seq\_179 77495 77501 See win3\_8674 on genome browser  
 win1\_8678 87103185 87103191 seq\_179 80086 80091 See win1\_8678 on genome browser  
 win1\_8681 87118686 87118690 seq\_179 95587 95590 See win1\_8681 on genome browser  
 win3\_8682 87127268 87127275 seq\_179 104169 104175 See win3\_8682 on genome browser  
 win2\_8686 87143497 87143504 seq\_179 120398 120404 See win2\_8686 on genome browser  
 win2\_8690 87149307 87149314 seq\_179 126208 126214 See win2\_8690 on genome browser  
 win3\_8694 87161645 87161652 seq\_179 138546 138552 See win3\_8694 on genome browser  
 win3\_8698 87168731 87168738 seq\_179 145632 145638 See win3\_8698 on genome browser  
 win2\_8702 87171125 87171132 seq\_179 148026 148032 See win2\_8702 on genome browser  
 win2\_8706 87175523 87175530 seq\_179 152424 152430 See win2\_8706 on genome browser  
 win3\_8710 87177724 87177731 seq\_179 154625 154631 See win3\_8710 on genome browser  
 win3\_8714 87263014 87263021 seq\_181 67352 67358 See win3\_8714 on genome browser  
 win3\_8718 87265788 87265795 seq\_181 70126 70132 See win3\_8718 on genome browser  
 win2\_8722 87282642 87282649 seq\_181 86980 86986 See win2\_8722 on genome browser  
 win3\_8726 87312233 87312240 seq\_181 116571 116577 See win3\_8726 on genome browser  
 win2\_8730 87319659 87319666 seq\_181 123997 124003 See win2\_8730 on genome browser  
 win2\_8734 87379760 87379767 seq\_182 11959 11965 See win2\_8734 on genome browser  
 win3\_8738 87398640 87398647 seq\_182 30839 30845 See win3\_8738 on genome browser  
 win2\_8742 87524008 87524015 seq\_182 156207 156213 See win2\_8742 on genome browser  
 win2\_8746 87528180 87528187 seq\_182 160379 160385 See win2\_8746 on genome browser  
 win2\_8750 87540876 87540883 seq\_183 1554 1560 See win2\_8750 on genome browser  
 win3\_8754 87541006 87541013 seq\_183 1684 1690 See win3\_8754 on genome browser  
 win3\_8758 87648036 87648043 seq\_183 108714 108720 See win3\_8758 on genome browser  
 win1\_8762 87673455 87673459 seq\_183 134133 134136 See win1\_8762 on genome browser  
 win3\_8763 87675111 87675118 seq\_183 135789 135795 See win3\_8763 on genome browser  
 win3\_8767 87675283 87675290 seq\_183 135961 135967 See win3\_8767 on genome browser  
 win3\_8771 87937292 87937299 seq\_184 55512 55518 See win3\_8771 on genome browser  
 win3\_8775 87939322 87939329 seq\_184 57542 57548 See win3\_8775 on genome browser  
 win2\_8779 87949165 87949172 seq\_184 67385 67391 See win2\_8779 on genome browser  
 win2\_8783 87956758 87956765 seq\_184 74978 74984 See win2\_8783 on genome browser  
 win3\_8787 88037278 88037285 seq\_184 155498 155504 See win3\_8787 on genome browser  
 win2\_8791 88037352 88037359 seq\_184 155572 155578 See win2\_8791 on genome browser  
 win2\_8795 88037453 88037460 seq\_184 155673 155679 See win2\_8795 on genome browser  
 win3\_8799 88053837 88053844 seq\_185 1288 1294 See win3\_8799 on genome browser  
 win2\_8803 88055098 88055105 seq\_185 2549 2555 See win2\_8803 on genome browser  
 win3\_8807 88056199 88056206 seq\_185 3650 3656 See win3\_8807 on genome browser  
 win2\_8811 88101919 88101926 seq\_185 49370 49376 See win2\_8811 on genome browser  
 win3\_8815 88153589 88153596 seq\_185 101040 101046 See win3\_8815 on genome browser  
 win2\_8819 88154382 88154389 seq\_185 101833 101839 See win2\_8819 on genome browser  
 win3\_8823 88162020 88162027 seq\_185 109471 109477 See win3\_8823 on genome browser  
 win2\_8827 88163525 88163532 seq\_185 110976 110982 See win2\_8827 on genome browser  
 win3\_8831 88167899 88167906 seq\_185 115350 115356 See win3\_8831 on genome browser  
 win3\_8835 88191518 88191525 seq\_185 138969 138975 See win3\_8835 on genome browser  
 win1\_8839 88193107 88193114 seq\_185 140558 140564 See win1\_8839 on genome browser  
 win2\_8843 88195301 88195308 seq\_185 142752 142758 See win2\_8843 on genome browser  
 win3\_8847 88205170 88205177 seq\_185 152621 152627 See win3\_8847 on genome browser  
 win1\_8851 88206552 88206559 seq\_185 154003 154009 See win1\_8851 on genome browser  
 win2\_8855 88407621 88407628 seq\_3819 18372 18378 See win2\_8855 on genome browser  
 win1\_8859 88419812 88419819 seq\_3819 30563 30569 See win1\_8859 on genome browser  
 win2\_8863 88526759 88526766 seq\_3819 137510 137516 See win2\_8863 on genome browser  
 win3\_8867 88528393 88528400 seq\_3819 139144 139150 See win3\_8867 on genome browser  
 win2\_8871 88544426 88544433 seq\_3819 155177 155183 See win2\_8871 on genome browser  
 win1\_8875 88607987 88607994 seq\_187 53522 53528 See win1\_8875 on genome browser  
 win2\_8879 88647251 88647258 seq\_187 92786 92792 See win2\_8879 on genome browser  
 win2\_8883 88689268 88689275 seq\_187 134803 134809 See win2\_8883 on genome browser  
 win3\_8887 88820860 88820867 seq\_188 102989 102995 See win3\_8887 on genome browser  
 win2\_8891 88887989 88887996 seq\_3761 6930 6936 See win2\_8891 on genome browser  
 win2\_8895 88890076 88890083 seq\_3761 9017 9023 See win2\_8895 on genome browser  
 win3\_8899 88903210 88903217 seq\_3761 22151 22157 See win3\_8899 on genome browser  
 win1\_8903 88958952 88958956 seq\_3761 77893 77896 See win1\_8903 on genome browser  
 win2\_8904 89026170 89026177 seq\_3761 145111 145117 See win2\_8904 on genome browser  
 win3\_8908 89117510 89117517 seq\_3820 75100 75106 See win3\_8908 on genome browser  
 win2\_8912 89117678 89117685 seq\_3820 75268 75274 See win2\_8912 on genome browser  
 win2\_8916 89192681 89192688 seq\_3820 150271 150277 See win2\_8916 on genome browser  
 win1\_8921 89396571 89396578 seq\_193 35232 35238 See win1\_8921 on genome browser  
 win1\_8925 89409050 89409057 seq\_193 47711 47717 See win1\_8925 on genome browser  
 win3\_8929 89411817 89411824 seq\_193 50478 50484 See win3\_8929 on genome browser  
 win3\_8933 89420876 89420883 seq\_193 59537 59543 See win3\_8933 on genome browser  
 win2\_8937 89427284 89427291 seq\_193 65945 65951 See win2\_8937 on genome browser  
 win3\_8941 89435053 89435060 seq\_193 73714 73720 See win3\_8941 on genome browser  
 win2\_8945 89450156 89450163 seq\_193 88817 88823 See win2\_8945 on genome browser  
 win3\_8949 89456941 89456948 seq\_193 95602 95608 See win3\_8949 on genome browser  
 win3\_8953 89492852 89492859 seq\_193 131513 131519 See win3\_8953 on genome browser  
 win2\_8957 89502474 89502481 seq\_193 141135 141141 See win2\_8957 on genome browser  
 win3\_8961 89526442 89526449 seq\_194 13327 13333 See win3\_8961 on genome browser  
 win2\_8965 89533108 89533115 seq\_194 19993 19999 See win2\_8965 on genome browser  
 win2\_8969 89544750 89544757 seq\_194 31635 31641 See win2\_8969 on genome browser  
 win3\_8973 89589851 89589858 seq\_194 76736 76742 See win3\_8973 on genome browser  
 win3\_8977 89598300 89598307 seq\_194 85185 85191 See win3\_8977 on genome browser  
 win2\_8981 89612027 89612034 seq\_194 98912 98918 See win2\_8981 on genome browser  
 win3\_8985 89644227 89644234 seq\_194 131112 131118 See win3\_8985 on genome browser  
 win3\_8989 89668359 89668366 seq\_195 3974 3980 See win3\_8989 on genome browser  
 win2\_8993 89671714 89671721 seq\_195 7329 7335 See win2\_8993 on genome browser  
 win3\_8997 89680345 89680352 seq\_195 15960 15966 See win3\_8997 on genome browser  
 win3\_9001 89691044 89691051 seq\_195 26659 26665 See win3\_9001 on genome browser  
 win3\_9005 89702009 89702016 seq\_195 37624 37630 See win3\_9005 on genome browser  
 win2\_9009 89702206 89702213 seq\_195 37821 37827 See win2\_9009 on genome browser  
 win3\_9013 89743395 89743402 seq\_195 79010 79016 See win3\_9013 on genome browser  
 win3\_9017 89772354 89772361 seq\_195 107969 107975 See win3\_9017 on genome browser  
 win3\_9021 89782575 89782582 seq\_195 118190 118196 See win3\_9021 on genome browser  
 win3\_9025 89790773 89790780 seq\_195 126388 126394 See win3\_9025 on genome browser  
 win2\_9029 89887676 89887683 seq\_3746 72965 72971 See win2\_9029 on genome browser  
 win3\_9033 90122664 90122671 seq\_196 11463 11469 See win3\_9033 on genome browser  
 win1\_9037 90195232 90195239 seq\_196 84031 84037 See win1\_9037 on genome browser  
 win3\_9041 90239428 90239435 seq\_196 128227 128233 See win3\_9041 on genome browser  
 win2\_9045 90240030 90240037 seq\_196 128829 128835 See win2\_9045 on genome browser  
 win3\_9049 90240974 90240981 seq\_196 129773 129779 See win3\_9049 on genome browser  
 win3\_9053 90283215 90283222 seq\_199 24852 24858 See win3\_9053 on genome browser  
 win2\_9057 90287570 90287577 seq\_199 29207 29213 See win2\_9057 on genome browser  
 win3\_9061 90288909 90288916 seq\_199 30546 30552 See win3\_9061 on genome browser  
 win2\_9065 90291945 90291952 seq\_199 33582 33588 See win2\_9065 on genome browser  
 win3\_9069 90301941 90301948 seq\_199 43578 43584 See win3\_9069 on genome browser  
 win2\_9073 90303196 90303203 seq\_199 44833 44839 See win2\_9073 on genome browser  
 win2\_9077 90343543 90343550 seq\_199 85180 85186 See win2\_9077 on genome browser  
 win2\_9081 90351203 90351210 seq\_199 92840 92846 See win2\_9081 on genome browser  
 win2\_9085 90357940 90357947 seq\_199 99577 99583 See win2\_9085 on genome browser  
 win2\_9089 90362314 90362321 seq\_199 103951 103957 See win2\_9089 on genome browser  
 win3\_9093 90369020 90369027 seq\_199 110657 110663 See win3\_9093 on genome browser  
 win2\_9097 90375814 90375821 seq\_199 117451 117457 See win2\_9097 on genome browser  
 win1\_9101 90378147 90378152 seq\_199 119784 119788 See win1\_9101 on genome browser  
 win1\_9103 90381155 90381159 seq\_199 122792 122795 See win1\_9103 on genome browser  
 win2\_9104 90383616 90383623 seq\_199 125253 125259 See win2\_9104 on genome browser  
 win2\_9108 90391523 90391530 seq\_199 133160 133166 See win2\_9108 on genome browser  
 win3\_9112 90391976 90391983 seq\_199 133613 133619 See win3\_9112 on genome browser  
 win2\_9116 90393195 90393202 seq\_199 134832 134838 See win2\_9116 on genome browser  
 win3\_9120 90592633 90592640 seq\_201 44004 44010 See win3\_9120 on genome browser  
 win3\_9124 90598930 90598937 seq\_201 50301 50307 See win3\_9124 on genome browser  
 win1\_9128 90637045 90637051 seq\_201 88416 88421 See win1\_9128 on genome browser  
 win1\_9131 90644162 90644169 seq\_201 95533 95539 See win1\_9131 on genome browser  
 win3\_9135 90650508 90650513 seq\_201 101879 101883 See win3\_9135 on genome browser  
 win2\_9137 90651855 90651862 seq\_201 103226 103232 See win2\_9137 on genome browser  
 win3\_9141 90656981 90656988 seq\_201 108352 108358 See win3\_9141 on genome browser  
 win2\_9145 90676929 90676936 seq\_201 128300 128306 See win2\_9145 on genome browser  
 win2\_9149 90679102 90679109 seq\_201 130473 130479 See win2\_9149 on genome browser  
 win1\_9153 90692756 90692760 seq\_202 810 813 See win1\_9153 on genome browser  
 win2\_9154 90698469 90698476 seq\_202 6523 6529 See win2\_9154 on genome browser  
 win1\_9158 90701652 90701657 seq\_202 9706 9710 See win1\_9158 on genome browser  
 win3\_9160 90732201 90732208 seq\_202 40255 40261 See win3\_9160 on genome browser  
 win3\_9164 90762041 90762048 seq\_202 70095 70101 See win3\_9164 on genome browser  
 win3\_9168 90820305 90820312 seq\_202 128359 128365 See win3\_9168 on genome browser  
 win3\_9172 90851575 90851582 seq\_3803 17645 17651 See win3\_9172 on genome browser  
 win3\_9176 91148733 91148740 seq\_204 34070 34076 See win3\_9176 on genome browser  
 win3\_9180 91150675 91150682 seq\_204 36012 36018 See win3\_9180 on genome browser  
 win3\_9184 91157320 91157327 seq\_204 42657 42663 See win3\_9184 on genome browser  
 win3\_9188 91223274 91223281 seq\_204 108611 108617 See win3\_9188 on genome browser  
 win2\_9192 91235256 91235263 seq\_204 120593 120599 See win2\_9192 on genome browser  
 win2\_9196 91236231 91236238 seq\_204 121568 121574 See win2\_9196 on genome browser  
 win3\_9200 91237209 91237216 seq\_204 122546 122552 See win3\_9200 on genome browser  
 win1\_9204 91267418 91267425 seq\_205 13482 13488 See win1\_9204 on genome browser  
 win2\_9208 91288048 91288055 seq\_205 34112 34118 See win2\_9208 on genome browser  
 win3\_9212 91318614 91318621 seq\_205 64678 64684 See win3\_9212 on genome browser  
 win3\_9216 91333470 91333477 seq\_205 79534 79540 See win3\_9216 on genome browser  
 win3\_9220 91339821 91339828 seq\_205 85885 85891 See win3\_9220 on genome browser  
 win3\_9224 91373169 91373176 seq\_205 119233 119239 See win3\_9224 on genome browser  
 win3\_9228 91381267 91381274 seq\_205 127331 127337 See win3\_9228 on genome browser  
 win2\_9232 91382497 91382504 seq\_205 128561 128567 See win2\_9232 on genome browser  
 win2\_9236 91499737 91499744 seq\_206 107049 107055 See win2\_9236 on genome browser  
 win3\_9240 91513744 91513751 seq\_206 121056 121062 See win3\_9240 on genome browser  
 win3\_9244 91536084 91536091 seq\_207 4749 4755 See win3\_9244 on genome browser  
 win2\_9248 91537522 91537529 seq\_207 6187 6193 See win2\_9248 on genome browser  
 win2\_9252 91539423 91539430 seq\_207 8088 8094 See win2\_9252 on genome browser  
 win3\_9256 91540429 91540436 seq\_207 9094 9100 See win3\_9256 on genome browser  
 win2\_9260 91542447 91542454 seq\_207 11112 11118 See win2\_9260 on genome browser  
 win2\_9264 91547423 91547430 seq\_207 16088 16094 See win2\_9264 on genome browser  
 win1\_9268 91548848 91548853 seq\_207 17513 17517 See win1\_9268 on genome browser  
 win3\_9270 91553597 91553604 seq\_207 22262 22268 See win3\_9270 on genome browser  
 win2\_9274 91566215 91566222 seq\_207 34880 34886 See win2\_9274 on genome browser  
 win2\_9278 91568982 91568989 seq\_207 37647 37653 See win2\_9278 on genome browser  
 win2\_9282 91596651 91596658 seq\_207 65316 65322 See win2\_9282 on genome browser  
 win3\_9286 91677051 91677058 seq\_3760 7251 7257 See win3\_9286 on genome browser  
 win3\_9290 91682592 91682599 seq\_3760 12792 12798 See win3\_9290 on genome browser  
 win3\_9294 91753507 91753514 seq\_3760 83707 83713 See win3\_9294 on genome browser  
 win1\_9298 91790259 91790263 seq\_3760 120459 120462 See win1\_9298 on genome browser  
 win3\_9299 91807437 91807444 seq\_3758 128 134 See win3\_9299 on genome browser  
 win3\_9303 91951782 91951789 seq\_210 7938 7944 See win3\_9303 on genome browser  
 win2\_9307 91994406 91994413 seq\_210 50562 50568 See win2\_9307 on genome browser  
 win1\_9311 92054413 92054418 seq\_210 110569 110573 See win1\_9311 on genome browser  
 win2\_9313 92064568 92064575 seq\_210 120724 120730 See win2\_9313 on genome browser  
 win1\_9317 92066732 92066738 seq\_210 122888 122893 See win1\_9317 on genome browser  
 win2\_9320 92088088 92088095 seq\_212 8073 8079 See win2\_9320 on genome browser  
 win3\_9324 92155229 92155234 seq\_212 75214 75218 See win3\_9324 on genome browser  
 win2\_9326 92155297 92155304 seq\_212 75282 75288 See win2\_9326 on genome browser  
 win3\_9330 92155304 92155311 seq\_212 75289 75295 See win3\_9330 on genome browser  
 win2\_9334 92175200 92175207 seq\_212 95185 95191 See win2\_9334 on genome browser  
 win3\_9338 92175236 92175243 seq\_212 95221 95227 See win3\_9338 on genome browser  
 win2\_9342 92203442 92203449 seq\_212 123427 123433 See win2\_9342 on genome browser  
 win3\_9346 92218219 92218226 seq\_213 2482 2488 See win3\_9346 on genome browser  
 win3\_9350 92239061 92239068 seq\_213 23324 23330 See win3\_9350 on genome browser  
 win3\_9354 92248842 92248849 seq\_213 33105 33111 See win3\_9354 on genome browser  
 win3\_9358 92255581 92255588 seq\_213 39844 39850 See win3\_9358 on genome browser  
 win1\_9362 92338038 92338045 seq\_213 122301 122307 See win1\_9362 on genome browser  
 win3\_9366 92339476 92339483 seq\_213 123739 123745 See win3\_9366 on genome browser  
 win2\_9370 92339570 92339577 seq\_213 123833 123839 See win2\_9370 on genome browser  
 win2\_9374 92386121 92386128 seq\_214 35447 35453 See win2\_9374 on genome browser  
 win3\_9378 92387801 92387808 seq\_214 37127 37133 See win3\_9378 on genome browser  
 win3\_9382 92412512 92412519 seq\_214 61838 61844 See win3\_9382 on genome browser  
 win2\_9386 92425168 92425175 seq\_214 74494 74500 See win2\_9386 on genome browser  
 win3\_9390 92428219 92428226 seq\_214 77545 77551 See win3\_9390 on genome browser  
 win2\_9394 92459354 92459361 seq\_214 108680 108686 See win2\_9394 on genome browser  
 win3\_9398 92469441 92469446 seq\_214 118767 118771 See win3\_9398 on genome browser  
 win2\_9400 92472064 92472071 seq\_214 121390 121396 See win2\_9400 on genome browser  
 win3\_9404 92496341 92496348 seq\_3809 12356 12362 See win3\_9404 on genome browser  
 win1\_9408 92544808 92544815 seq\_3809 60823 60829 See win1\_9408 on genome browser  
 win3\_9412 92646552 92646559 seq\_216 31287 31293 See win3\_9412 on genome browser  
 win2\_9416 92648872 92648879 seq\_216 33607 33613 See win2\_9416 on genome browser  
 win2\_9420 92670748 92670755 seq\_216 55483 55489 See win2\_9420 on genome browser  
 win2\_9424 92670888 92670895 seq\_216 55623 55629 See win2\_9424 on genome browser  
 win3\_9428 92684036 92684043 seq\_216 68771 68777 See win3\_9428 on genome browser  
 win1\_9432 92699110 92699116 seq\_216 83845 83850 See win1\_9432 on genome browser  
 win2\_9435 92701175 92701182 seq\_216 85910 85916 See win2\_9435 on genome browser  
 win2\_9439 92709347 92709354 seq\_216 94082 94088 See win2\_9439 on genome browser  
 win3\_9443 92715126 92715133 seq\_216 99861 99867 See win3\_9443 on genome browser  
 win3\_9447 92749512 92749519 seq\_217 3061 3067 See win3\_9447 on genome browser  
 win3\_9451 92762585 92762592 seq\_217 16134 16140 See win3\_9451 on genome browser  
 win2\_9455 92790249 92790256 seq\_217 43798 43804 See win2\_9455 on genome browser  
 win1\_9459 92799590 92799597 seq\_217 53139 53145 See win1\_9459 on genome browser  
 win3\_9463 92877027 92877034 seq\_218 393 399 See win3\_9463 on genome browser  
 win3\_9467 92878348 92878355 seq\_218 1714 1720 See win3\_9467 on genome browser  
 win1\_9471 92898809 92898813 seq\_218 22175 22178 See win1\_9471 on genome browser  
 win2\_9472 92905263 92905270 seq\_218 28629 28635 See win2\_9472 on genome browser  
 win3\_9476 92911733 92911740 seq\_218 35099 35105 See win3\_9476 on genome browser  
 win2\_9480 92912952 92912959 seq\_218 36318 36324 See win2\_9480 on genome browser  
 win1\_9484 92914542 92914549 seq\_218 37908 37914 See win1\_9484 on genome browser  
 win2\_9488 92914624 92914631 seq\_218 37990 37996 See win2\_9488 on genome browser  
 win3\_9492 92925041 92925048 seq\_218 48407 48413 See win3\_9492 on genome browser  
 win3\_9496 92987954 92987961 seq\_218 111320 111326 See win3\_9496 on genome browser  
 win2\_9500 92989475 92989482 seq\_218 112841 112847 See win2\_9500 on genome browser  
 win3\_9504 92990570 92990577 seq\_218 113936 113942 See win3\_9504 on genome browser  
 win2\_9508 92991559 92991566 seq\_218 114925 114931 See win2\_9508 on genome browser  
 win3\_9512 93260735 93260742 seq\_3798 393 399 See win3\_9512 on genome browser  
 win3\_9516 93262705 93262712 seq\_3798 2363 2369 See win3\_9516 on genome browser  
 win3\_9520 93264818 93264825 seq\_3798 4476 4482 See win3\_9520 on genome browser  
 win3\_9524 93267486 93267493 seq\_3798 7144 7150 See win3\_9524 on genome browser  
 win2\_9528 93272179 93272186 seq\_3798 11837 11843 See win2\_9528 on genome browser  
 win3\_9532 93455161 93455168 seq\_3799 69179 69185 See win3\_9532 on genome browser  
 win2\_9536 93486821 93486828 seq\_3799 100839 100845 See win2\_9536 on genome browser  
 win3\_9540 93486899 93486906 seq\_3799 100917 100923 See win3\_9540 on genome browser  
 win2\_9544 93515204 93515211 seq\_220 4395 4401 See win2\_9544 on genome browser  
 win1\_9548 93541211 93541218 seq\_220 30402 30408 See win1\_9548 on genome browser  
 win2\_9552 93620197 93620204 seq\_220 109388 109394 See win2\_9552 on genome browser  
 win2\_9556 93638357 93638364 seq\_223 3038 3044 See win2\_9556 on genome browser  
 win2\_9560 93644036 93644043 seq\_223 8717 8723 See win2\_9560 on genome browser  
 win3\_9564 93670415 93670422 seq\_223 35096 35102 See win3\_9564 on genome browser  
 win3\_9568 93675939 93675946 seq\_223 40620 40626 See win3\_9568 on genome browser  
 win2\_9572 93681636 93681643 seq\_223 46317 46323 See win2\_9572 on genome browser  
 win3\_9576 93715501 93715508 seq\_223 80182 80188 See win3\_9576 on genome browser  
 win2\_9580 93715605 93715612 seq\_223 80286 80292 See win2\_9580 on genome browser  
 win2\_9584 93715785 93715792 seq\_223 80466 80472 See win2\_9584 on genome browser  
 win3\_9588 93765745 93765752 seq\_224 7326 7332 See win3\_9588 on genome browser  
 win3\_9592 93771413 93771420 seq\_224 12994 13000 See win3\_9592 on genome browser  
 win2\_9596 93776354 93776361 seq\_224 17935 17941 See win2\_9596 on genome browser  
 win3\_9600 93881131 93881138 seq\_3801 16 22 See win3\_9600 on genome browser  
 win3\_9604 93939974 93939981 seq\_3801 58859 58865 See win3\_9604 on genome browser  
 win3\_9608 94005657 94005664 seq\_227 2247 2253 See win3\_9608 on genome browser  
 win2\_9612 94023551 94023558 seq\_227 20141 20147 See win2\_9612 on genome browser  
 win3\_9616 94027092 94027099 seq\_227 23682 23688 See win3\_9616 on genome browser  
 win2\_9620 94027987 94027994 seq\_227 24577 24583 See win2\_9620 on genome browser  
 win3\_9624 94037220 94037227 seq\_227 33810 33816 See win3\_9624 on genome browser  
 win2\_9628 94047509 94047516 seq\_227 44099 44105 See win2\_9628 on genome browser  
 win3\_9632 94055787 94055794 seq\_227 52377 52383 See win3\_9632 on genome browser  
 win3\_9636 94060972 94060979 seq\_227 57562 57568 See win3\_9636 on genome browser  
 win3\_9640 94064544 94064551 seq\_227 61134 61140 See win3\_9640 on genome browser  
 win2\_9644 94067614 94067621 seq\_227 64204 64210 See win2\_9644 on genome browser  
 win3\_9648 94067646 94067653 seq\_227 64236 64242 See win3\_9648 on genome browser  
 win3\_9652 94068113 94068120 seq\_227 64703 64709 See win3\_9652 on genome browser  
 win3\_9656 94102725 94102732 seq\_227 99315 99321 See win3\_9656 on genome browser  
 win3\_9660 94102844 94102851 seq\_227 99434 99440 See win3\_9660 on genome browser  
 win2\_9664 94103366 94103373 seq\_227 99956 99962 See win2\_9664 on genome browser  
 win1\_9668 94104408 94104414 seq\_227 100998 101003 See win1\_9668 on genome browser  
 win3\_9671 94174616 94174623 seq\_3815 49826 49832 See win3\_9671 on genome browser  
 win3\_9675 94253489 94253496 seq\_3806 9830 9836 See win3\_9675 on genome browser  
 win3\_9679 94362523 94362530 seq\_3816 28 34 See win3\_9679 on genome browser  
 win1\_9683 94424259 94424264 seq\_3816 61764 61768 See win1\_9683 on genome browser  
 win3\_9685 94432385 94432392 seq\_3816 69890 69896 See win3\_9685 on genome browser  
 win2\_9689 94719749 94719756 seq\_3797 17165 17171 See win2\_9689 on genome browser  
 win2\_9693 94738755 94738762 seq\_3797 36171 36177 See win2\_9693 on genome browser  
 win1\_9697 94824481 94824486 seq\_233 10501 10505 See win1\_9697 on genome browser  
 win2\_9699 94827690 94827697 seq\_233 13710 13716 See win2\_9699 on genome browser  
 win3\_9703 94832252 94832259 seq\_233 18272 18278 See win3\_9703 on genome browser  
 win2\_9707 94837100 94837109 seq\_233 23120 23128 See win2\_9707 on genome browser  
 win2\_9713 94840759 94840766 seq\_233 26779 26785 See win2\_9713 on genome browser  
 win3\_9717 94855881 94855888 seq\_233 41901 41907 See win3\_9717 on genome browser  
 win3\_9721 94862279 94862286 seq\_233 48299 48305 See win3\_9721 on genome browser  
 win3\_9725 94870873 94870880 seq\_233 56893 56899 See win3\_9725 on genome browser  
 win2\_9729 94874944 94874951 seq\_233 60964 60970 See win2\_9729 on genome browser  
 win3\_9733 94894334 94894341 seq\_233 80354 80360 See win3\_9733 on genome browser  
 win3\_9737 94897077 94897084 seq\_233 83097 83103 See win3\_9737 on genome browser  
 win3\_9741 94927974 94927981 seq\_234 3116 3122 See win3\_9741 on genome browser  
 win2\_9745 94931140 94931147 seq\_234 6282 6288 See win2\_9745 on genome browser  
 win1\_9749 94944568 94944572 seq\_234 19710 19713 See win1\_9749 on genome browser  
 win2\_9750 94945278 94945285 seq\_234 20420 20426 See win2\_9750 on genome browser  
 win2\_9754 94949612 94949619 seq\_234 24754 24760 See win2\_9754 on genome browser  
 win3\_9758 94951021 94951028 seq\_234 26163 26169 See win3\_9758 on genome browser  
 win2\_9762 94953003 94953010 seq\_234 28145 28151 See win2\_9762 on genome browser  
 win3\_9766 94957703 94957710 seq\_234 32845 32851 See win3\_9766 on genome browser  
 win2\_9770 94964813 94964820 seq\_234 39955 39961 See win2\_9770 on genome browser  
 win2\_9774 94966884 94966891 seq\_234 42026 42032 See win2\_9774 on genome browser  
 win3\_9778 94975601 94975608 seq\_234 50743 50749 See win3\_9778 on genome browser  
 win1\_9782 94979452 94979459 seq\_234 54594 54600 See win1\_9782 on genome browser  
 win3\_9786 95010074 95010081 seq\_234 85216 85222 See win3\_9786 on genome browser  
 win2\_9790 95017964 95017971 seq\_234 93106 93112 See win2\_9790 on genome browser  
 win3\_9794 95022505 95022512 seq\_234 97647 97653 See win3\_9794 on genome browser  
 win2\_9798 95148334 95148341 seq\_235 2092 2098 See win2\_9798 on genome browser  
 win3\_9802 95228410 95228417 seq\_235 82168 82174 See win3\_9802 on genome browser  
 win2\_9806 95437850 95437857 seq\_240 71896 71902 See win2\_9806 on genome browser  
 win3\_9810 95455734 95455741 seq\_240 89780 89786 See win3\_9810 on genome browser  
 win2\_9814 95455926 95455933 seq\_240 89972 89978 See win2\_9814 on genome browser  
 win3\_9818 95471253 95471260 seq\_241 0 6 See win3\_9818 on genome browser  
 win2\_9822 95473592 95473599 seq\_241 2339 2345 See win2\_9822 on genome browser  
 win3\_9826 95483124 95483131 seq\_241 11871 11877 See win3\_9826 on genome browser  
 win2\_9830 95485635 95485642 seq\_241 14382 14388 See win2\_9830 on genome browser  
 win3\_9834 95489771 95489778 seq\_241 18518 18524 See win3\_9834 on genome browser  
 win2\_9838 95493961 95493968 seq\_241 22708 22714 See win2\_9838 on genome browser  
 win3\_9842 95494062 95494069 seq\_241 22809 22815 See win3\_9842 on genome browser  
 win2\_9846 95501526 95501533 seq\_241 30273 30279 See win2\_9846 on genome browser  
 win3\_9850 95505876 95505883 seq\_241 34623 34629 See win3\_9850 on genome browser  
 win3\_9854 95517486 95517493 seq\_241 46233 46239 See win3\_9854 on genome browser  
 win2\_9858 95537055 95537062 seq\_241 65802 65808 See win2\_9858 on genome browser  
 win3\_9862 95542516 95542523 seq\_241 71263 71269 See win3\_9862 on genome browser  
 win2\_9866 95546300 95546307 seq\_241 75047 75053 See win2\_9866 on genome browser  
 win3\_9870 95552378 95552385 seq\_241 81125 81131 See win3\_9870 on genome browser  
 win3\_9874 95563803 95563810 seq\_241 92550 92556 See win3\_9874 on genome browser  
 win1\_9878 95586237 95586242 seq\_242 10143 10147 See win1\_9878 on genome browser  
 win3\_9880 95600779 95600786 seq\_242 24685 24691 See win3\_9880 on genome browser  
 win2\_9884 95600974 95600981 seq\_242 24880 24886 See win2\_9884 on genome browser  
 win2\_9888 95605627 95605634 seq\_242 29533 29539 See win2\_9888 on genome browser  
 win2\_9892 95637982 95637989 seq\_242 61888 61894 See win2\_9892 on genome browser  
 win2\_9896 95686555 95686562 seq\_243 6324 6330 See win2\_9896 on genome browser  
 win2\_9900 95697112 95697119 seq\_243 16881 16887 See win2\_9900 on genome browser  
 win3\_9904 95697230 95697237 seq\_243 16999 17005 See win3\_9904 on genome browser  
 win3\_9908 95701930 95701937 seq\_243 21699 21705 See win3\_9908 on genome browser  
 win3\_9912 95763081 95763088 seq\_243 82850 82856 See win3\_9912 on genome browser  
 win3\_9916 95766406 95766413 seq\_243 86175 86181 See win3\_9916 on genome browser  
 win3\_9920 95831539 95831546 seq\_3817 48207 48213 See win3\_9920 on genome browser  
 win2\_9924 95831744 95831751 seq\_3817 48412 48418 See win2\_9924 on genome browser  
 win2\_9928 95869653 95869660 seq\_3817 86321 86327 See win2\_9928 on genome browser  
 win2\_9932 95873523 95873530 seq\_3817 90191 90197 See win2\_9932 on genome browser  
 win2\_9936 95885091 95885098 seq\_245 1441 1447 See win2\_9936 on genome browser  
 win3\_9940 95895705 95895712 seq\_245 12055 12061 See win3\_9940 on genome browser  
 win2\_9944 95914956 95914963 seq\_245 31306 31312 See win2\_9944 on genome browser  
 win2\_9948 95939569 95939576 seq\_245 55919 55925 See win2\_9948 on genome browser  
 win3\_9952 95942601 95942608 seq\_245 58951 58957 See win3\_9952 on genome browser  
 win2\_9956 95942953 95942960 seq\_245 59303 59309 See win2\_9956 on genome browser  
 win2\_9960 95984403 95984410 seq\_246 1440 1446 See win2\_9960 on genome browser  
 win2\_9964 96021769 96021776 seq\_246 38806 38812 See win2\_9964 on genome browser  
 win3\_9968 96021869 96021876 seq\_246 38906 38912 See win3\_9968 on genome browser  
 win2\_9972 96055918 96055925 seq\_246 72955 72961 See win2\_9972 on genome browser  
 win3\_9976 96081383 96081390 seq\_248 -4 2 See win3\_9976 on genome browser  
 win2\_9980 96086384 96086391 seq\_248 4997 5003 See win2\_9980 on genome browser  
 win3\_9984 96097128 96097135 seq\_248 15741 15747 See win3\_9984 on genome browser  
 win2\_9988 96146109 96146116 seq\_248 64722 64728 See win2\_9988 on genome browser  
 win3\_9992 96167606 96167613 seq\_248 86219 86225 See win3\_9992 on genome browser  
 win1\_9996 96183042 96183047 seq\_249 3627 3631 See win1\_9996 on genome browser  
 win2\_9998 96186105 96186112 seq\_249 6690 6696 See win2\_9998 on genome browser  
 win1\_10002 96192992 96192998 seq\_249 13577 13582 See win1\_10002 on genome browser  
 win2\_10005 96205655 96205662 seq\_249 26240 26246 See win2\_10005 on genome browser  
 win3\_10009 96209122 96209129 seq\_249 29707 29713 See win3\_10009 on genome browser  
 win3\_10013 96217492 96217499 seq\_249 38077 38083 See win3\_10013 on genome browser  
 win3\_10017 96219662 96219669 seq\_249 40247 40253 See win3\_10017 on genome browser  
 win2\_10021 96220713 96220720 seq\_249 41298 41304 See win2\_10021 on genome browser  
 win2\_10025 96223842 96223849 seq\_249 44427 44433 See win2\_10025 on genome browser  
 win3\_10029 96233013 96233020 seq\_249 53598 53604 See win3\_10029 on genome browser  
 win3\_10033 96233232 96233239 seq\_249 53817 53823 See win3\_10033 on genome browser  
 win2\_10037 96233401 96233408 seq\_249 53986 53992 See win2\_10037 on genome browser  
 win2\_10041 96237597 96237604 seq\_249 58182 58188 See win2\_10041 on genome browser  
 win2\_10045 96246680 96246687 seq\_249 67265 67271 See win2\_10045 on genome browser  
 win2\_10049 96248264 96248271 seq\_249 68849 68855 See win2\_10049 on genome browser  
 win3\_10053 96283284 96283291 seq\_250 5885 5891 See win3\_10053 on genome browser  
 win3\_10057 96285121 96285128 seq\_250 7722 7728 See win3\_10057 on genome browser  
 win3\_10061 96299335 96299342 seq\_250 21936 21942 See win3\_10061 on genome browser  
 win1\_10065 96305263 96305269 seq\_250 27864 27869 See win1\_10065 on genome browser  
 win3\_10068 96313493 96313500 seq\_250 36094 36100 See win3\_10068 on genome browser  
 win2\_10072 96341990 96341997 seq\_250 64591 64597 See win2\_10072 on genome browser  
 win3\_10076 96363824 96363831 seq\_250 86425 86431 See win3\_10076 on genome browser  
 win2\_10080 96363980 96363987 seq\_250 86581 86587 See win2\_10080 on genome browser  
 win3\_10084 96391955 96391962 seq\_253 17285 17291 See win3\_10084 on genome browser  
 win2\_10088 96407652 96407659 seq\_253 32982 32988 See win2\_10088 on genome browser  
 win3\_10092 96428688 96428695 seq\_253 54018 54024 See win3\_10092 on genome browser  
 win3\_10096 96440427 96440434 seq\_253 65757 65763 See win3\_10096 on genome browser  
 win2\_10100 96461335 96461342 seq\_253 86665 86671 See win2\_10100 on genome browser  
 win3\_10104 96482137 96482143 seq\_254 10800 10805 See win3\_10104 on genome browser  
 win3\_10107 96515457 96515464 seq\_254 44120 44126 See win3\_10107 on genome browser  
 win2\_10111 96525271 96525278 seq\_254 53934 53940 See win2\_10111 on genome browser  
 win3\_10115 96542015 96542022 seq\_254 70678 70684 See win3\_10115 on genome browser  
 win3\_10119 96568065 96568072 seq\_255 2660 2666 See win3\_10119 on genome browser  
 win3\_10123 96570405 96570412 seq\_255 5000 5006 See win3\_10123 on genome browser  
 win3\_10127 96572726 96572733 seq\_255 7321 7327 See win3\_10127 on genome browser  
 win3\_10131 96588628 96588635 seq\_255 23223 23229 See win3\_10131 on genome browser  
 win2\_10135 96592746 96592753 seq\_255 27341 27347 See win2\_10135 on genome browser  
 win3\_10139 96599900 96599907 seq\_255 34495 34501 See win3\_10139 on genome browser  
 win2\_10143 96633631 96633638 seq\_255 68226 68232 See win2\_10143 on genome browser  
 win3\_10147 96646724 96646731 seq\_255 81319 81325 See win3\_10147 on genome browser  
 win2\_10151 96697817 96697824 seq\_3826 39068 39074 See win2\_10151 on genome browser  
 win3\_10155 96779454 96779461 seq\_3796 27531 27537 See win3\_10155 on genome browser  
 win2\_10159 96807264 96807271 seq\_3796 55341 55347 See win2\_10159 on genome browser  
 win2\_10163 96832812 96832819 seq\_3796 80889 80895 See win2\_10163 on genome browser  
 win2\_10167 96846115 96846122 seq\_257 3009 3015 See win2\_10167 on genome browser  
 win2\_10171 96848832 96848839 seq\_257 5726 5732 See win2\_10171 on genome browser  
 win3\_10175 96889417 96889424 seq\_257 46311 46317 See win3\_10175 on genome browser  
 win2\_10179 96913403 96913410 seq\_257 70297 70303 See win2\_10179 on genome browser  
 win3\_10183 96915348 96915355 seq\_257 72242 72248 See win3\_10183 on genome browser  
 win2\_10187 96934741 96934748 seq\_258 942 948 See win2\_10187 on genome browser  
 win2\_10191 96944472 96944479 seq\_258 10673 10679 See win2\_10191 on genome browser  
 win3\_10195 96953088 96953095 seq\_258 19289 19295 See win3\_10195 on genome browser  
 win3\_10199 96987695 96987702 seq\_258 53896 53902 See win3\_10199 on genome browser  
 win2\_10203 96991428 96991435 seq\_258 57629 57635 See win2\_10203 on genome browser  
 win2\_10207 97012757 97012764 seq\_258 78958 78964 See win2\_10207 on genome browser  
 win3\_10211 97042008 97042015 seq\_3804 17579 17585 See win3\_10211 on genome browser  
 win2\_10215 97051580 97051587 seq\_3804 27151 27157 See win2\_10215 on genome browser  
 win2\_10219 97077617 97077624 seq\_3804 53188 53194 See win2\_10219 on genome browser  
 win3\_10223 97126971 97126978 seq\_261 13174 13180 See win3\_10223 on genome browser  
 win2\_10227 97143658 97143665 seq\_261 29861 29867 See win2\_10227 on genome browser  
 win3\_10231 97179377 97179384 seq\_261 65580 65586 See win3\_10231 on genome browser  
 win2\_10235 97191627 97191634 seq\_261 77830 77836 See win2\_10235 on genome browser  
 win3\_10239 97219546 97219553 seq\_263 16793 16799 See win3\_10239 on genome browser  
 win3\_10243 97224823 97224830 seq\_263 22070 22076 See win3\_10243 on genome browser  
 win2\_10247 97245928 97245935 seq\_263 43175 43181 See win2\_10247 on genome browser  
 win3\_10251 97289088 97289095 seq\_265 254 260 See win3\_10251 on genome browser  
 win2\_10255 97378256 97378263 seq\_266 3501 3507 See win2\_10255 on genome browser  
 win2\_10259 97412535 97412542 seq\_266 37780 37786 See win2\_10259 on genome browser  
 win2\_10263 97421178 97421185 seq\_266 46423 46429 See win2\_10263 on genome browser  
 win2\_10267 97426186 97426193 seq\_266 51431 51437 See win2\_10267 on genome browser  
 win2\_10271 97426312 97426319 seq\_266 51557 51563 See win2\_10271 on genome browser  
 win3\_10275 97427567 97427574 seq\_266 52812 52818 See win3\_10275 on genome browser  
 win2\_10279 97432843 97432850 seq\_266 58088 58094 See win2\_10279 on genome browser  
 win3\_10283 97447032 97447039 seq\_266 72277 72283 See win3\_10283 on genome browser  
 win1\_10287 97448977 97448983 seq\_266 74222 74227 See win1\_10287 on genome browser  
 win2\_10290 97467576 97467583 seq\_267 7252 7258 See win2\_10290 on genome browser  
 win3\_10294 97470496 97470503 seq\_267 10172 10178 See win3\_10294 on genome browser  
 win2\_10298 97470776 97470783 seq\_267 10452 10458 See win2\_10298 on genome browser  
 win3\_10302 97470810 97470817 seq\_267 10486 10492 See win3\_10302 on genome browser  
 win2\_10306 97472435 97472442 seq\_267 12111 12117 See win2\_10306 on genome browser  
 win3\_10310 97473512 97473519 seq\_267 13188 13194 See win3\_10310 on genome browser  
 win3\_10314 97475662 97475669 seq\_267 15338 15344 See win3\_10314 on genome browser  
 win3\_10318 97496342 97496349 seq\_267 36018 36024 See win3\_10318 on genome browser  
 win2\_10322 97499867 97499874 seq\_267 39543 39549 See win2\_10322 on genome browser  
 win3\_10326 97532378 97532385 seq\_267 72054 72060 See win3\_10326 on genome browser  
 win1\_10330 97545068 97545073 seq\_268 634 638 See win1\_10330 on genome browser  
 win2\_10332 97545206 97545213 seq\_268 772 778 See win2\_10332 on genome browser  
 win2\_10336 97571683 97571690 seq\_268 27249 27255 See win2\_10336 on genome browser  
 win2\_10340 97576739 97576746 seq\_268 32305 32311 See win2\_10340 on genome browser  
 win3\_10344 97580592 97580599 seq\_268 36158 36164 See win3\_10344 on genome browser  
 win2\_10348 97584678 97584685 seq\_268 40244 40250 See win2\_10348 on genome browser  
 win2\_10352 97591048 97591055 seq\_268 46614 46620 See win2\_10352 on genome browser  
 win2\_10356 97602091 97602098 seq\_268 57657 57663 See win2\_10356 on genome browser  
 win3\_10360 97602192 97602199 seq\_268 57758 57764 See win3\_10360 on genome browser  
 win2\_10364 97605756 97605763 seq\_268 61322 61328 See win2\_10364 on genome browser  
 win3\_10368 97605856 97605863 seq\_268 61422 61428 See win3\_10368 on genome browser  
 win3\_10372 97606730 97606737 seq\_268 62296 62302 See win3\_10372 on genome browser  
 win2\_10376 97608790 97608797 seq\_268 64356 64362 See win2\_10376 on genome browser  
 win2\_10380 97616484 97616491 seq\_268 72050 72056 See win2\_10380 on genome browser  
 win2\_10384 97669928 97669935 seq\_269 41502 41508 See win2\_10384 on genome browser  
 win3\_10388 97751828 97751835 seq\_270 39844 39850 See win3\_10388 on genome browser  
 win3\_10392 97751888 97751895 seq\_270 39904 39910 See win3\_10392 on genome browser  
 win2\_10396 97901200 97901207 seq\_272 23847 23853 See win2\_10396 on genome browser  
 win2\_10400 97909414 97909421 seq\_272 32061 32067 See win2\_10400 on genome browser  
 win2\_10404 97943313 97943320 seq\_272 65960 65966 See win2\_10404 on genome browser  
 win3\_10408 98055518 98055525 seq\_274 15102 15108 See win3\_10408 on genome browser  
 win3\_10412 98092953 98092960 seq\_274 52537 52543 See win3\_10412 on genome browser  
 win2\_10416 98095883 98095890 seq\_274 55467 55473 See win2\_10416 on genome browser  
 win3\_10420 98162485 98162492 seq\_275 42497 42503 See win3\_10420 on genome browser  
 win3\_10424 98174632 98174639 seq\_275 54644 54650 See win3\_10424 on genome browser  
 win1\_10428 98176436 98176441 seq\_275 56448 56452 See win1\_10428 on genome browser  
 win2\_10430 98229870 98229877 seq\_278 30730 30736 See win2\_10430 on genome browser  
 win2\_10434 98231108 98231115 seq\_278 31968 31974 See win2\_10434 on genome browser  
 win2\_10438 98231326 98231333 seq\_278 32186 32192 See win2\_10438 on genome browser  
 win3\_10442 98253567 98253574 seq\_278 54427 54433 See win3\_10442 on genome browser  
 win2\_10446 98277467 98277474 seq\_279 1849 1855 See win2\_10446 on genome browser  
 win3\_10450 98281646 98281653 seq\_279 6028 6034 See win3\_10450 on genome browser  
 win2\_10454 98285748 98285755 seq\_279 10130 10136 See win2\_10454 on genome browser  
 win3\_10458 98323768 98323775 seq\_279 48150 48156 See win3\_10458 on genome browser  
 win2\_10462 98352248 98352255 seq\_280 848 854 See win2\_10462 on genome browser  
 win2\_10466 98371622 98371629 seq\_280 20222 20228 See win2\_10466 on genome browser  
 win3\_10470 98375420 98375427 seq\_280 24020 24026 See win3\_10470 on genome browser  
 win3\_10474 98377096 98377103 seq\_280 25696 25702 See win3\_10474 on genome browser  
 win2\_10478 98389696 98389703 seq\_280 38296 38302 See win2\_10478 on genome browser  
 win3\_10482 98409475 98409482 seq\_280 58075 58081 See win3\_10482 on genome browser  
 win3\_10486 98412637 98412644 seq\_280 61237 61243 See win3\_10486 on genome browser  
 win2\_10490 98433758 98433765 seq\_281 7014 7020 See win2\_10490 on genome browser  
 win1\_10494 98466465 98466471 seq\_281 39721 39726 See win1\_10494 on genome browser  
 win2\_10497 98482574 98482581 seq\_281 55830 55836 See win2\_10497 on genome browser  
 win3\_10501 98483538 98483545 seq\_281 56794 56800 See win3\_10501 on genome browser  
 win2\_10505 98557106 98557113 seq\_282 55057 55063 See win2\_10505 on genome browser  
 win2\_10509 98579902 98579909 seq\_283 2832 2838 See win2\_10509 on genome browser  
 win1\_10513 98600929 98600933 seq\_283 23859 23862 See win1\_10513 on genome browser  
 win1\_10514 98602487 98602494 seq\_283 25417 25423 See win1\_10514 on genome browser  
 win2\_10518 98608458 98608465 seq\_283 31388 31394 See win2\_10518 on genome browser  
 win3\_10522 98610128 98610135 seq\_283 33058 33064 See win3\_10522 on genome browser  
 win3\_10526 98627982 98627989 seq\_283 50912 50918 See win3\_10526 on genome browser  
 win1\_10530 98629540 98629545 seq\_283 52470 52474 See win1\_10530 on genome browser  
 win2\_10532 98632116 98632123 seq\_283 55046 55052 See win2\_10532 on genome browser  
 win2\_10536 98641721 98641728 seq\_283 64651 64657 See win2\_10536 on genome browser  
 win2\_10540 98669658 98669665 seq\_284 17935 17941 See win2\_10540 on genome browser  
 win1\_10544 98670713 98670718 seq\_284 18990 18994 See win1\_10544 on genome browser  
 win2\_10546 98681159 98681166 seq\_284 29436 29442 See win2\_10546 on genome browser  
 win3\_10550 98684650 98684657 seq\_284 32927 32933 See win3\_10550 on genome browser  
 win2\_10554 98696460 98696467 seq\_284 44737 44743 See win2\_10554 on genome browser  
 win2\_10558 98697409 98697416 seq\_284 45686 45692 See win2\_10558 on genome browser  
 win3\_10562 98705967 98705974 seq\_284 54244 54250 See win3\_10562 on genome browser  
 win3\_10566 98735439 98735446 seq\_285 9178 9184 See win3\_10566 on genome browser  
 win3\_10570 98775569 98775576 seq\_285 49308 49314 See win3\_10570 on genome browser  
 win2\_10574 98777417 98777424 seq\_285 51156 51162 See win2\_10574 on genome browser  
 win2\_10578 98803186 98803193 seq\_286 2642 2648 See win2\_10578 on genome browser  
 win1\_10582 98804148 98804155 seq\_286 3604 3610 See win1\_10582 on genome browser  
 win3\_10586 98809767 98809774 seq\_286 9223 9229 See win3\_10586 on genome browser  
 win3\_10590 98812607 98812614 seq\_286 12063 12069 See win3\_10590 on genome browser  
 win2\_10594 98813396 98813403 seq\_286 12852 12858 See win2\_10594 on genome browser  
 win2\_10598 98820139 98820146 seq\_286 19595 19601 See win2\_10598 on genome browser  
 win3\_10602 98825020 98825027 seq\_286 24476 24482 See win3\_10602 on genome browser  
 win3\_10606 98833149 98833156 seq\_286 32605 32611 See win3\_10606 on genome browser  
 win1\_10610 98849477 98849484 seq\_286 48933 48939 See win1\_10610 on genome browser  
 win3\_10614 98927097 98927104 seq\_287 52716 52722 See win3\_10614 on genome browser  
 win3\_10618 98952191 98952198 seq\_288 4195 4201 See win3\_10618 on genome browser  
 win3\_10622 98953114 98953121 seq\_288 5118 5124 See win3\_10622 on genome browser  
 win3\_10626 98953262 98953269 seq\_288 5266 5272 See win3\_10626 on genome browser  
 win3\_10630 98961227 98961234 seq\_288 13231 13237 See win3\_10630 on genome browser  
 win3\_10634 98962563 98962570 seq\_288 14567 14573 See win3\_10634 on genome browser  
 win2\_10638 98962786 98962793 seq\_288 14790 14796 See win2\_10638 on genome browser  
 win3\_10642 99009892 99009899 seq\_288 61896 61902 See win3\_10642 on genome browser  
 win3\_10646 99055701 99055708 seq\_289 35100 35106 See win3\_10646 on genome browser  
 win2\_10650 99061258 99061265 seq\_289 40657 40663 See win2\_10650 on genome browser  
 win3\_10654 99068784 99068791 seq\_289 48183 48189 See win3\_10654 on genome browser  
 win3\_10658 99080368 99080375 seq\_289 59767 59773 See win3\_10658 on genome browser  
 win3\_10662 99174166 99174173 seq\_291 9553 9559 See win3\_10662 on genome browser  
 win2\_10666 99178738 99178745 seq\_291 14125 14131 See win2\_10666 on genome browser  
 win1\_10670 99180654 99180659 seq\_291 16041 16045 See win1\_10670 on genome browser  
 win3\_10672 99208213 99208220 seq\_291 43600 43606 See win3\_10672 on genome browser  
 win2\_10676 99210469 99210476 seq\_291 45856 45862 See win2\_10676 on genome browser  
 win3\_10680 99244632 99244639 seq\_292 8605 8611 See win3\_10680 on genome browser  
 win2\_10684 99280071 99280078 seq\_292 44044 44050 See win2\_10684 on genome browser  
 win1\_10688 99289184 99289191 seq\_292 53157 53163 See win1\_10688 on genome browser  
 win2\_10692 99292277 99292284 seq\_292 56250 56256 See win2\_10692 on genome browser  
 win1\_10696 99337526 99337533 seq\_293 31704 31710 See win1\_10696 on genome browser  
 win3\_10700 99353101 99353108 seq\_293 47279 47285 See win3\_10700 on genome browser  
 win3\_10704 99358838 99358845 seq\_293 53016 53022 See win3\_10704 on genome browser  
 win3\_10708 99364448 99364455 seq\_293 58626 58632 See win3\_10708 on genome browser  
 win1\_10712 99377447 99377454 seq\_294 2610 2616 See win1\_10712 on genome browser  
 win2\_10716 99446706 99446713 seq\_295 2933 2939 See win2\_10716 on genome browser  
 win1\_10720 99448114 99448119 seq\_295 4341 4345 See win1\_10720 on genome browser  
 win3\_10722 99449913 99449920 seq\_295 6140 6146 See win3\_10722 on genome browser  
 win3\_10726 99459728 99459735 seq\_295 15955 15961 See win3\_10726 on genome browser  
 win3\_10730 99473664 99473671 seq\_295 29891 29897 See win3\_10730 on genome browser  
 win2\_10734 99480999 99481006 seq\_295 37226 37232 See win2\_10734 on genome browser  
 win3\_10738 99486118 99486125 seq\_295 42345 42351 See win3\_10738 on genome browser  
 win2\_10742 99492303 99492310 seq\_295 48530 48536 See win2\_10742 on genome browser  
 win2\_10746 99498792 99498799 seq\_295 55019 55025 See win2\_10746 on genome browser  
 win3\_10750 99499521 99499528 seq\_295 55748 55754 See win3\_10750 on genome browser  
 win2\_10754 99499790 99499797 seq\_295 56017 56023 See win2\_10754 on genome browser  
 win2\_10758 99500213 99500220 seq\_295 56440 56446 See win2\_10758 on genome browser  
 win1\_10762 99514429 99514436 seq\_296 2391 2397 See win1\_10762 on genome browser  
 win1\_10766 99551497 99551502 seq\_296 39459 39463 See win1\_10766 on genome browser  
 win2\_10768 99553437 99553444 seq\_296 41399 41405 See win2\_10768 on genome browser  
 win3\_10772 99593638 99593645 seq\_297 13991 13997 See win3\_10772 on genome browser  
 win3\_10776 99622244 99622251 seq\_297 42597 42603 See win3\_10776 on genome browser  
 win3\_10780 99715504 99715511 seq\_299 1545 1551 See win3\_10780 on genome browser  
 win3\_10784 99716661 99716668 seq\_299 2702 2708 See win3\_10784 on genome browser  
 win3\_10788 99739041 99739048 seq\_299 25082 25088 See win3\_10788 on genome browser  
 win1\_10792 99741441 99741445 seq\_299 27482 27485 See win1\_10792 on genome browser  
 win3\_10793 99752462 99752469 seq\_299 38503 38509 See win3\_10793 on genome browser  
 win2\_10797 99815212 99815219 seq\_301 34846 34852 See win2\_10797 on genome browser  
 win2\_10801 99821357 99821364 seq\_301 40991 40997 See win2\_10801 on genome browser  
 win3\_10805 99828377 99828384 seq\_301 48011 48017 See win3\_10805 on genome browser  
 win3\_10809 99833766 99833773 seq\_301 53400 53406 See win3\_10809 on genome browser  
 win3\_10813 99895590 99895597 seq\_302 49671 49677 See win3\_10813 on genome browser  
 win3\_10817 99897819 99897826 seq\_302 51900 51906 See win3\_10817 on genome browser  
 win3\_10821 99932737 99932744 seq\_304 21425 21431 See win3\_10821 on genome browser  
 win3\_10825 99978395 99978402 seq\_305 2055 2061 See win3\_10825 on genome browser  
 win3\_10829 100023334 100023341 seq\_305 46994 47000 See win3\_10829 on genome browser  
 win3\_10833 100047332 100047339 seq\_306 6157 6163 See win3\_10833 on genome browser  
 win3\_10837 100053659 100053666 seq\_306 12484 12490 See win3\_10837 on genome browser  
 win1\_10841 100054932 100054937 seq\_306 13757 13761 See win1\_10841 on genome browser  
 win3\_10843 100086060 100086067 seq\_306 44885 44891 See win3\_10843 on genome browser  
 win3\_10847 100087238 100087245 seq\_306 46063 46069 See win3\_10847 on genome browser  
 win3\_10851 100110280 100110287 seq\_307 4301 4307 See win3\_10851 on genome browser  
 win2\_10855 100112043 100112050 seq\_307 6064 6070 See win2\_10855 on genome browser  
 win1\_10859 100119631 100119638 seq\_307 13652 13658 See win1\_10859 on genome browser  
 win3\_10863 100131850 100131857 seq\_307 25871 25877 See win3\_10863 on genome browser  
 win3\_10867 100132604 100132611 seq\_307 26625 26631 See win3\_10867 on genome browser  
 win3\_10871 100140319 100140326 seq\_307 34340 34346 See win3\_10871 on genome browser  
 win3\_10875 100142230 100142237 seq\_307 36251 36257 See win3\_10875 on genome browser  
 win3\_10879 100171399 100171406 seq\_308 633 639 See win3\_10879 on genome browser  
 win2\_10883 100173913 100173920 seq\_308 3147 3153 See win2\_10883 on genome browser  
 win2\_10887 100186305 100186312 seq\_308 15539 15545 See win2\_10887 on genome browser  
 win1\_10891 100200769 100200773 seq\_308 30003 30006 See win1\_10891 on genome browser  
 win3\_10892 100204869 100204876 seq\_308 34103 34109 See win3\_10892 on genome browser  
 win2\_10896 100237142 100237149 seq\_309 1981 1987 See win2\_10896 on genome browser  
 win2\_10900 100246550 100246557 seq\_309 11389 11395 See win2\_10900 on genome browser  
 win2\_10904 100257172 100257179 seq\_309 22011 22017 See win2\_10904 on genome browser  
 win1\_10908 100272464 100272471 seq\_309 37303 37309 See win1\_10908 on genome browser  
 win1\_10912 100300705 100300710 seq\_310 2193 2197 See win1\_10912 on genome browser  
 win2\_10914 100302704 100302711 seq\_310 4192 4198 See win2\_10914 on genome browser  
 win3\_10918 100425359 100425366 seq\_312 2064 2070 See win3\_10918 on genome browser  
 win3\_10922 100495136 100495143 seq\_313 10172 10178 See win3\_10922 on genome browser  
 win2\_10926 100498546 100498553 seq\_313 13582 13588 See win2\_10926 on genome browser  
 win3\_10930 100502530 100502537 seq\_313 17566 17572 See win3\_10930 on genome browser  
 win2\_10934 100507336 100507343 seq\_313 22372 22378 See win2\_10934 on genome browser  
 win3\_10938 100532135 100532142 seq\_313 47171 47177 See win3\_10938 on genome browser  
 win2\_10942 100532693 100532700 seq\_313 47729 47735 See win2\_10942 on genome browser  
 win3\_10946 100532793 100532800 seq\_313 47829 47835 See win3\_10946 on genome browser  
 win3\_10950 100535380 100535387 seq\_313 50416 50422 See win3\_10950 on genome browser  
 win2\_10954 100670310 100670317 seq\_316 1912 1918 See win2\_10954 on genome browser  
 win2\_10958 100671993 100672000 seq\_316 3595 3601 See win2\_10958 on genome browser  
 win2\_10962 100674872 100674879 seq\_316 6474 6480 See win2\_10962 on genome browser  
 win2\_10966 100679007 100679014 seq\_316 10609 10615 See win2\_10966 on genome browser  
 win3\_10970 100686684 100686691 seq\_316 18286 18292 See win3\_10970 on genome browser  
 win2\_10974 100698866 100698873 seq\_316 30468 30474 See win2\_10974 on genome browser  
 win3\_10978 100708188 100708195 seq\_316 39790 39796 See win3\_10978 on genome browser  
 win3\_10982 100716120 100716127 seq\_316 47722 47728 See win3\_10982 on genome browser  
 win3\_10986 100729314 100729321 seq\_317 134 140 See win3\_10986 on genome browser  
 win2\_10990 100731030 100731037 seq\_317 1850 1856 See win2\_10990 on genome browser
